# Functional Specialization of the Medial Temporal Lobes in Human Recognition Memory: Dissociating Effects of Hippocampal versus Parahippocampal Damage

**DOI:** 10.1093/cercor/bhab290

**Published:** 2021-09-17

**Authors:** Georgios P D Argyropoulos, Carola Dell’Acqua, Emily Butler, Clare Loane, Adriana Roca-Fernandez, Azhaar Almozel, Nikolas Drummond, Carmen Lage-Martinez, Elisa Cooper, Richard N Henson, Christopher R Butler

**Affiliations:** Memory Research Group, Nuffield Department of Clinical Neurosciences, University of Oxford, Oxford OX3 9DU, UK; Division of Psychology, Faculty of Natural Sciences, University of Stirling, Stirling FK9 4LA, UK; Memory Research Group, Nuffield Department of Clinical Neurosciences, University of Oxford, Oxford OX3 9DU, UK; Department of General Psychology and Padova Neuroscience Center, University of Padova, 35131 Padova, Italy; Memory Research Group, Nuffield Department of Clinical Neurosciences, University of Oxford, Oxford OX3 9DU, UK; Memory Research Group, Nuffield Department of Clinical Neurosciences, University of Oxford, Oxford OX3 9DU, UK; Basic and Clinical Neuroscience Department, Maurice Wohl Clinical Neuroscience Institute, King’s College London, 5 Cutcombe Rd, London SE5 9RT, UK; Memory Research Group, Nuffield Department of Clinical Neurosciences, University of Oxford, Oxford OX3 9DU, UK; Memory Research Group, Nuffield Department of Clinical Neurosciences, University of Oxford, Oxford OX3 9DU, UK; School of Biosciences, Cardiff University, Cardiff CF10 3AX, UK; Memory Research Group, Nuffield Department of Clinical Neurosciences, University of Oxford, Oxford OX3 9DU, UK; Department of Zoology, University of Cambridge, Cambridge CB2 3EJ, UK; Memory Research Group, Nuffield Department of Clinical Neurosciences, University of Oxford, Oxford OX3 9DU, UK; Valdecilla Biomedical Research Institute, University Hospital Marqués de Valdecilla, 39011 Santander, Spain; MRC Cognition and Brain Sciences Unit and Department of Psychiatry, University of Cambridge, Cambridge CB2 7EF, UK; MRC Cognition and Brain Sciences Unit and Department of Psychiatry, University of Cambridge, Cambridge CB2 7EF, UK; Memory Research Group, Nuffield Department of Clinical Neurosciences, University of Oxford, Oxford OX3 9DU, UK; Department of Brain Sciences, Imperial College London, London W12 0NN, UK; Departamento de Neurología, Pontificia Universidad Católica de Chile, Avda. Libertador Bernando O'Higgins 340, Santiago, Chile

**Keywords:** amnesia, familiarity, memory, MRI, recollection

## Abstract

A central debate in the systems neuroscience of memory concerns whether different medial temporal lobe (MTL) structures support different processes in recognition memory. Using two recognition memory paradigms, we tested a rare patient (MH) with a perirhinal lesion that appeared to spare the hippocampus. Consistent with a similar previous case, MH showed impaired familiarity and preserved recollection. When compared with patients with hippocampal lesions appearing to spare perirhinal cortex, MH showed greater impairment on familiarity and less on recollection. Nevertheless, the hippocampal patients also showed impaired familiarity compared with healthy controls. However, when replacing this traditional categorization of patients with analyses relating memory performance to continuous measures of damage across patients, hippocampal volume uniquely predicted recollection, whereas parahippocampal, rather than perirhinal, volume uniquely predicted familiarity. We consider whether the familiarity impairment in MH and our patients with hippocampal lesions arises from “subthreshold” damage to parahippocampal cortex (PHC). Our data provide the most compelling neuropsychological support yet for dual-process models of recognition memory, whereby recollection and familiarity depend on different MTL structures, and may support a role for PHC in familiarity. Our study highlights the value of supplementing single-case studies with examinations of continuous brain–behavior relationships across larger patient groups.

## Introduction

Ever since the first descriptions of the famous patient HM (Scoville and Milner 1957), individuals with medial temporal lobe (MTL) damage have been fundamental in delineating the brain regions supporting human memory. Patient studies offer crucial insights into causal brain–behavior relationships, beyond the correlational information afforded by functional imaging in healthy participants. Nevertheless, many questions about the neural basis of human memory remain unresolved (Squire and Wixted 2011).

In particular, competing accounts have been offered to explain the impact of MTL damage on recognition memory, that is, the capacity to discriminate whether or not stimuli have been encountered recently. A central question relates to “process-specificity”—whether distinct MTL structures support different processes underlying recognition memory, such as “recollection” (remembering the context in which a stimulus occurred; fundamental for recall) versus “familiarity” (a feeling that a stimulus was encountered, without retrieval of contextual information). According to prominent “dual-process” frameworks (Aggleton and Brown 1999; Montaldi and Mayes 2010), recollection relies on the hippocampus (HPC), whereas familiarity relies on regions within the parahippocampal gyrus, principally the perirhinal cortex [PRC; though there is also evidence implicating the parahippocampal cortex (PHC) in familiarity processes, e.g., O’Kane et al. 2005; Martin et al. 2013, 2018; Gimbel et al. 2017]. This framework predicts a double dissociation between memory processes, with impaired recollection but not familiarity following selective HPC lesions, and impaired familiarity but not recollection following selective PRC lesions. The main opposing “unitary account” (Wixted and Squire 2011a), however, posits that recollection and familiarity do not dissociate within the MTL, such that both recollection and familiarity are supported by both the HPC and the PRC.

However, studies assessing these competing predictions face several challenges: 1) standardized neuropsychometric assessment does not suffice to dissociate recollection from familiarity (Argyropoulos and Butler 2020); 2) there is no universally accepted method of separating recollection and familiarity estimates, so convergent evidence from multiple methods is recommended; 3) selective HPC lesions are rare (Bird and Burgess 2008), since the conditions associated with these, for example, ischemia/anoxia, often also cause extra-MTL damage (Huang and Castillo 2008); 4) selective PRC lesions are even rarer still, with only two cases having been reported: patient IR (Inhoff et al. 2019) and patient NB (Köhler and Martin 2020). Patient IR had a right PRC lesion and showed perceptual deficits in the absence of memory deficits, though no MTL volumetry was reported, and the memory tasks were not designed to assess familiarity and recollection separately. More importantly, Patient NB did show memory impairments, with impaired familiarity but intact recollection, which is the opposite to the pattern normally reported for HPC lesions, where familiarity is less impaired than recollection. Patient NB’s PRC lesion is therefore vital in providing, in combination with HPC lesions, the double dissociation that favors dual-process theories over single-process theories (Montaldi and Mayes 2010).

A further challenge for distinguishing dual- versus single-process theories of MTL function concerns 5) potential interactions with material-type (Robin et al. 2019). It has been suggested that PRC is important for recognizing objects and faces, while the PHC, in the posterior parts of parahippocampal gyrus (Pruessner et al. 2002), may be important specifically for scene recognition (Montaldi and Mayes 2010). Moreover, the entorhinal cortex (ERC) may have specialized routes for object versus scene information, since input from the PRC and the PHC is conveyed into the HPC via different ERC subregions (Van Strien et al. 2009; Reagh and Yassa 2014; Maass et al. 2015). The HPC has also been claimed to be important for processing scenes (Zeidman et al. 2015), though others propose that its role in memory is independent of material-type (Kim et al. 2015). Material-specific theories provide complementary or even alternative accounts to dual/single-process theories (Lacot et al. 2017), and dissociations previously reported between processes (e.g., recollection vs. familiarity) may be specific to certain material types. Thus, it is important to examine recognition across multiple material types.

A final challenge for neuropsychological studies concerns 6) the number and definition of patients. Despite the historical influence of single-case studies such as HM and NB (Shallice 2019), testing theories on the basis of single individuals requires consideration of individual differences and measurement noise (Lambon Ralph et al. 2011). Furthermore, defining distinct groups of patients in terms of “selective” lesions to certain brain regions can be misleading (Cipolotti et al. 2006); these groupings are often based on structural brain imaging, from which regions are binarized into “lesioned” or “intact” according to some threshold relative to matched, healthy brains. This categorical approach may miss more subtle, “continuous” relationships between the degree of regional damage and the degree of memory impairment. This latter approach of studying brain–behavior correlations requires larger patient groups and leverages on individual variability in the distribution of brain damage and memory performance (Argyropoulos et al. 2019).

Here, we started by examining an exceptionally rare case of a patient with a lesion in the right PRC (Patient “MH”), which appeared to spare the HPC, and who appeared to have memory deficits similar to those reported by Köhler and Martin (2020), that is, impaired familiarity but intact recollection. In addition to standard neuropsychological tests, we tested him on two paradigms designed to isolate recollection and familiarity in different ways, with the potential to provide convergent evidence. To examine the alternative or moderating factor of material type, we compared performance on each paradigm when using unfamiliar faces versus unfamiliar scenes. Then, to see how he compared to other patients with MTL damage, we tested another 7 patients, who had MRI-confirmed HPC lesions, on the same paradigms. By comparing these two groups of patients (binarized as either “PRC-lesioned/HPC-intact” or “PRC-intact/HPC-lesioned”), we could test for a double dissociation in behavior (as a function of recollection and familiarity, and/or material type). However, damage within the MTL rarely respects anatomical boundaries (e.g., lesions isolated to the HPC are very rare, Bird and Burgess 2008), with multiple structures being affected in tandem with, or as knock-on effects of, relatively focal pathology (e.g., in the HPC, Argyropoulos et al. 2019; Loane et al. 2019). We thus also performed a continuous analysis across all patients between the volumes of the aforementioned regions of interest (ROIs)—that is, the HPC, PRC, ERC, and PHC—and performance on each paradigm. By avoiding the need for an arbitrary threshold of damage for group membership, and capturing potential effects of sub-threshold damage, this continuous approach can reveal insights beyond the categorical and, in particular, single-case approaches.

## Materials and Methods

### Participants

All participants provided written informed consent according to the Declaration of Helsinki. Ethical approval was received from South Central Oxford Research Ethics Committee (REC no: 08/H0606/133).

### Healthy Controls

For the MRI analyses below, patients were compared against a group of 48 healthy controls (CTRs; reported in Argyropoulos et al. 2019; age: median = 64.85; IQR = 15.56 years; sex: 23 M:25 F). Overall, the patients did not differ from the group of 48 CTRs in terms of M:F ratio (7 M:2 F; χ^2^ = 2.71, *P* = 0.100) or age at research scan (median = 56.93; IQR = 11.78; U = 148, *P* = 0.142). For the behavioral paradigms, 14 CTRs were recruited through local advertisement (only 6 had available MRI data), 8 M:6 F, with a mean age at behavioral assessment of 62.11 (SD = 6.20) years, and mean years in education of 13.00 (SD = 1.75). They were all native speakers of English, with no known psychiatric or neurological disorders. Due to scheduling conflicts and technical errors, 12/14 CTR datasets were available for the first paradigm and 9/14 for the second paradigm.

### Patients

All 9 patients (7 M:2 F; age at behavioral assessment: mean = 60.40; SD = 6.26 years; vs. CTRs: *t*(21) = 0.66, *P* = 0.520; education: mean = 12.22, SD = 1.09 years; vs. CTRs: *t*(21) = 1.19, *P* = 0.249) were recruited within the context of the Memory and Amnesia Project (https://www.ndcn.ox.ac.uk/research/memory-research-group/projects-1/memory-and-amnesia-project).

#### PRC Patient (MH)

**Figure 1:**
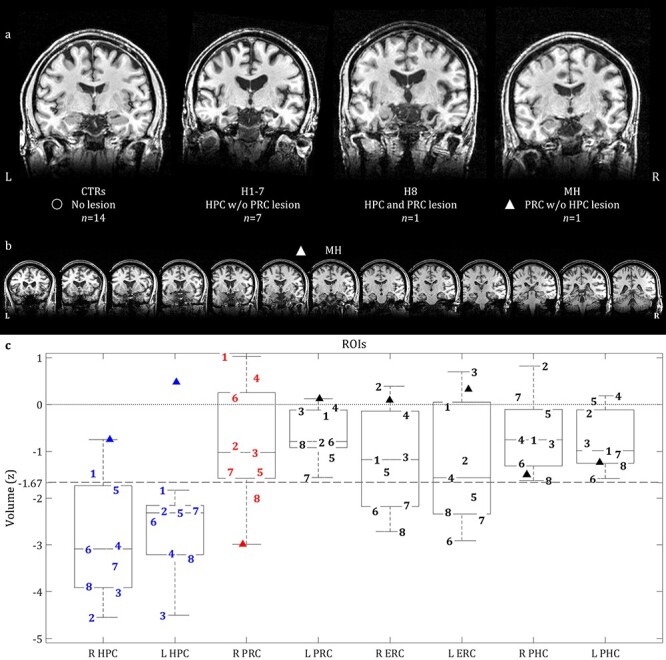
(*a*) Coronal slices of structural MR images of CTRs and patients; (*b*) a series of coronal slices for MH, highlighting his lesioned right PRC and spared left PRC, along with the rest of his spared MTL structures; (*c*) ROI volumes for all 9 patients; boxplots pertain to all 9 patients; line within each boxplot = median value; bottom of box = 25th %ile; top of box = 75th %ile; upper and lower whiskers = scores outside the middle 50; whiskers = 1.5 ^*^ interquartile range. **Key:** ▲, MH (patient with right PRC lesion); **1–7,** patients with HPC but no PRC lesion; **8,** patient with both HPC and right PRC lesions; **L, R,** left, right hemisphere; **PHC,** parahippocampal cortex; **ROI,** region of interest (L/R HPC, ERC, PRC, PHC); **Z,** volumes are corrected for TIV and then expressed as Z-scores, based on the mean and standard deviation of the volumes of 48 CTRs whose MTL structures were manually delineated (see Argyropoulos et al. 2019, for details); lesion defined as a Z < −1.67; horizontal lines: Z = 0 (dotted line) and Z = −1.67 (dashed line).

This man was 51 years of age at the time of study participation [which did not differ significantly from the mean of CTRs (*t*(13) = 1.69, *P* = 0.115) or from the mean of the rest of the patients (*t*(7) = 1.70, *P* = 0.132)] and had 12 years of education [which again did not differ significantly from the mean of CTRs (*t*(13) = 0.55, *P* = 0.591) or from the mean of the rest of the patients (*t*(7) = 0.20, *P* = 0.845)]. At the age of 21, while working, he collapsed on the floor and was hospitalized, where a clinical MRI showed a cerebral abscess in his right PRC, sparing the HPC and other MTL structures. The lesion is illustrated in Figure 1*a* as a hypointensity in the structural *T*_1_-weighted MRI that he underwent at the age of 48 as part of our research study. Volumetric analysis of this MRI (Fig. 1*b*) confirmed that the volume of his right PRC (*Z* = −2.99) was below a conventional cut-off of *Z* = −1.67 (i.e., 5th %ile) relative to CTRs. This was not true of any of the other MTL ROIs examined, that is, HPC, ERC, PRC, and PHC. No damage was seen in the left or right amygdala or temporal pole either (all *Z*s, *Z* > −1.19). A few years after the incident, he was diagnosed with focal epilepsy, and an EEG disclosed epileptiform activity in the right anterior temporal region. He was treated with the antiepileptic drug carbamazepine and the seizures remitted completely. Neuropsychological assessment (conducted at the age of 48) demonstrated normal levels of intelligence, language, executive function, visuospatial perception, visual and verbal recall, as well as verbal and visual recognition memory (all test scores: *Z* > −1.67), with the striking exception of recognition memory for faces (*Z* = −2.33) (Supplementary Table 1).

#### HPC Patients (H1–H8)

We also tested 8 further patients (“H1–H8”) who all had HPC lesions due to autoimmune limbic encephalitis, which was diagnosed according to consensus criteria (Graus et al. 2016). Volumetric analysis of their *T*_1_-weighted MRIs confirmed that all 8 of them had *Z*-scores below −1.67 relative to CTRs in either left, right, or both HPC (Fig. 1*b*). We call this group the “HPC group.” In some of these patients, their ERC volume was also below this cut-off, and for one of the patients (H8), the right PRC was below the cut-off (as well as the left amygdala). We excluded the latter patient for categorical analyses below, but included him in the continuous analysis. None of the remaining MTL ROIs showed volumes below this cut-off.

These patients were representative of the clinical and neuropsychological group-level profile of the autoimmune limbic encephalitis cohort presented in Argyropoulos et al. (2019): 1) they were all native speakers of English; 2) they were all recruited after the acute phase of the disease had resolved and were clinically stable (delay from symptom onset range: 1.77–14.92 years); 3) in their acute clinical *T*_2_-weighted MRI scans, all 8/8 patients had shown abnormalities in the HPC with respect to volume, *T*_2_ signal intensity, and/or diffusion (in one patient, there was also high *T*_2_-signal and swelling noted in the right amygdala); in 7/8 patients (H1–H7), these clinically defined abnormalities did not extend to the parahippocampal gyrus, whereas in the case of one patient (H8), abnormalities had been also noted in both the HPC and the parahippocampal gyrus, and he was the only HPC patient with a lesion in the (right) PRC; 4) no abnormalities were detected beyond the MTL in the research scan that patients underwent acutely or postacutely (delay from symptom onset range: 1.72–12.93 years); 5) in their postacute neuropsychological assessment (delay from symptom onset range: 1.69–12.93 years), they all showed average to above-average premorbid intelligence (National Adult Reading Test; Nelson and Willison 1991), along with 6) preserved (postmorbid) intelligence, semantic memory and language [Wechsler Abbreviated Scales of Intelligence: Vocabulary, Similarities, Matrices (Wechsler 2011); Graded Naming Test (Mckenna and Warrington 1980); Camel and Cactus test (Bozeat et al. 2000)]; 7) executive function [Delis–Kaplan Executive Function System—Trails: Number-Letter Switching (Delis et al. 2001)] including working memory [Wechsler Memory Scale: Digit Span forward and backward (Wechsler 1997)] (individual impairment on a test was defined as an age-scaled standardized score of ≤ −1.67, corresponding to the 5th %ile, in line with standard neuropsychological practice, e.g., Butler et al. 2014), and 8) visuospatial perception [Visual Object and Space Perception battery: cube analysis, dot counting, position discrimination (Warrington and James 1991)] (all scores above the 5th %ile cut-off point employed in this test); 9) none of the patients had a history of premorbid psychiatric or neurological disorder that could have resulted in cognitive impairment, and 10) none had any contraindication to MRI at the time of entry into the study. Importantly, 7/8 of the HPC patients showed impaired performance in at least one test of anterograde memory [Wechsler Memory Scale (Wechsler 1997); Rey-Osterrieth Complex Figure Test (Rey 1959); the Warrington Recognition Memory Tests for faces and words (Warrington 1984) and the Warrington Topographical Memory test for scenes (Warrington 1996); the Doors and People test (Baddeley et al. 1994)] (Supplementary Table 1). Interestingly, only patient H8 (the HPC patient who also had right a PRC lesion) showed impaired face recognition memory (like MH above).

### Scanning Procedures

We acquired 3D *T*_1_-weighted MRIs for all 9 patients (Siemens 3 T Trio system; 32-channel head coil; University of Oxford Centre for Clinical Magnetic Resonance Research) using a Magnetization Prepared Rapid Gradient Echo sequence (echo time = 4.7 ms; repetition time = 2040 ms; flip angle = 8°; field of view = 192 mm; voxel size = 1 × 1 × 1 mm) for all patients.

### Manual Volumetry

Manual segmentation of HPC and parahippocampal ROIs (left/right ERC, PRC, and PHC) was conducted in native space (using ITK-SNAP; Yushkevich et al. 2006) by a trained researcher (A.R.F.) according to segmentation procedures based on published atlases and protocols (Insausti et al. 1998; Pruessner et al. 2002), described in Loane et al. (2019). The volumes of all structures were corrected for (divided by) total intra-cranial volume (TIV), calculated from the unified segmentation procedure in SPM12.

### Whole-Brain Voxel-Based Morphometry (Modulated Gray Matter)

In order to ensure that our group of HPC patients (*n* = 8; H1–8) did not present with GM volume reduction beyond the MTL, we conducted a VBM analysis contrasting the whole-brain modulated GM tissue maps (reflecting GM volume) of the HPC patients against those of 67 datasets of CTRs (previously presented in Argyropoulos et al. 2019). VBM was conducted using the Statistical Parametric Mapping software (SPM12 version 7219; http://www.fil.ion.ucl.ac.uk/spm/software/spm12) in MATLAB R2020a. Images were examined for scanner artifacts and reoriented to have the same point of origin (anterior commissure) and spatial orientation. They were then bias-corrected to remove intensity nonuniformities and segmented into GM, white matter (WM), and cerebrospinal fluid (CSF) with the unified segmentation procedure. The diffeomorphic anatomical registration through the exponentiated lie algebra (DARTEL) toolbox was applied to participants’ GM, WM, and CSF to refine intersubject registration, and study-specific GM templates were generated (Ashburner 2007). After affine registration of the GM DARTEL templates to the tissue probability maps in MNI (Montreal Neurological Institute) space, nonlinear warping of GM images was performed to this template in MNI space. Voxel values in the tissue maps were modulated by the Jacobian determinant that was calculated during spatial normalization, with modulated GM images reflecting tissue volume. These images (voxel size: 1-mm^3^ isotropic) were smoothed using a Gaussian filter of 4-mm FWHM. We then compared GM volume between the group of 8 HPC patients and that of 67 CTRs [contrast: “CTRs > HPC patients”; second-level between-subject covariates: age, sex, TIV, study (see Argyropoulos et al. 2019 for details)]. We examined peaks surviving whole-brain FWE-correction (*P* < 0.05). Volume reduction was exclusively noted within the MTL (Supplementary Fig. 1). Given the effects of registration and smoothing in VBM, we relied on gold-standard manual volumetry to quantify MTL volumes and examine structure–behavior relationships.

### Behavioral Paradigms

Two recognition memory paradigms were employed to dissociate recollection from familiarity, and each paradigm repeated for unfamiliar human faces, unfamiliar natural scenes, and visually presented, high-frequency words. Given a technical error in the design of the task (Supplementary section 1), participants’ performance with word stimuli is not presented in the main text. Structure–behavior relationships including all three stimulus types are presented in Supplementary Tables 11–19.

### ROC

The first paradigm used receiver operating characteristics (ROCs), derived from the distribution of confidence responses across studied and unstudied items, for independent estimation of recollection and familiarity (see Yonelinas 1994; Yonelinas and Parks 2007 for methods; Supplementary Fig. 2). This method has been employed in several studies that examine the impact of MTL lesions on recognition memory (e.g., Yonelinas et al. 2002; Aggleton et al. 2005; Bowles et al. 2007).

#### Stimulus Materials

##### Faces

160 photos (targets: *n* = 80; foils: *n* = 80) of faces (front view) of unknown Caucasian individuals with a broad age range (18–91 years of age) were taken from the Face Database (Minear and Park 2004). All photos were taken under natural lighting and had a neutral gray background provided by a portable projection screen. The target and foil faces were matched for age (targets: M = 61.50, IQR = 48.00; foils: M = 58.00, IQR = 45.50) and for M:F ratio (targets: 24:56; foils: 26:54). They were presented in the center of the display.

##### Scenes

160 pictures of unfamiliar natural landscapes (targets: *n* = 80; foils: *n* = 80) were chosen from the royalty-free platform Shutterstock (https://www.shutterstock.com), to include no sign of manmade features (buildings, objects), or of people or animals. The scenes used as targets were selected to resemble those used for the foils with respect to their general theme (Autumn: 4:4; Beach: 6:5; Clouds: 4:4; Desert: 7:7; Forest: 5:6; Hills: 7:6; Lake: 5:6; Mountains: 8:7; River: 6:4; Rocks: 10:11; Sea: 4:6; Waterfalls: 4:6; Winter: 10:8). They were presented in the center of the display (17-cm wide, 11-cm tall).

##### Procedure

The experiment was written in MATLAB, using the Psychophysics Toolbox (v.3) extensions (Brainard 1997; Pelli 1997; Kleiner et al. 2007). Each participant was tested in a quiet room. The session lasted ~45 min. The order of trial blocks is illustrated in Supplementary Figure 2. Each stimulus was first presented to participants in the study phase, before testing recognition memory in the test phase. In both the study and test phases, each trial started with a fixation cross for 0.5 s at the center of the display, replaced by a stimulus. In the study phase, participants were asked to judge if each stimulus was “pleasant,” “neutral,” or “unpleasant.” Face and scene stimuli were presented for 4.5 s, irrespective of participants’ response latencies. In the test phase, participants were asked to judge whether the stimulus presented had been previously encountered in the study phase on a 6-point confidence scale (1 = definitely new; 2 = probably new; 3 = maybe new; 4 = maybe old; 5 = probably old; 6 = definitely old), in a self-paced fashion. This phase included all of the stimuli that had been previously presented in the study phase (targets), along with an equal number of novel stimuli (foils). Participants were asked to make full use of the confidence scale. The order of blocks and assignment of stimuli to conditions was kept constant across participants, in order to enable the comparison of individual patients with other patients/CTRs.

A filler task was also introduced in a series of blocks interspersed within the session, in order to minimize the influence of working memory, as well as to amplify forgetting between study and test phases. In each trial, two numbers were presented side-by-side at the center of the screen. Participants were required to answer a question below those two numbers, asking participants to decide which of the two numbers was higher or lower. Participants selected “1” for the number on the left, “2” for the number on the right, or “3” if the two numbers were equal. Participants were given 3 s to respond, before the new trial started.

#### Behavioral Data Analysis

The confidence ratings (ROCs) were analyzed with a dual-process model that assumed recollection and familiarity are independent processes (Yonelinas et al. 1996), using an algorithm available at https://dl.dropboxusercontent.com/s/csignrwvlqwr5it/DPSDSSE.xls?dl=1), implemented in MATLAB code (http://www.ruhr-unibochum.de/neuropsy/tests/memorysolve.zip), and reported in Pustina et al. (2012).

### RDP

The second paradigm that was used to provide estimates for recollection and familiarity was based on a response deadline procedure (RDP), which is predicated on the selective reliance of recognition memory on familiarity at short response deadlines, in contrast with long response deadlines (Supplementary Fig. 3) (Bowles et al. 2007). The paradigm was administered in two separate sessions, one with a short response deadline (800 ms), and the other with a long response deadline (2400 ms). The session including the long response deadline was administered first, with a minimum of a 5 days’ delay between the two sessions, so as to prevent interference from the first session in the second session. Patients and CTRs did not differ in the delay between the two sessions (Patients: M = 14; IQR = 227 days; CTRs: M = 14; IQR = 122.50 days; U = 38, *P* = 0.861). Moreover, we ensured that the first session of the RDP was administered on a different day from the ROC, with a minimum of a 1-day delay across participants. CTRs and patients did not differ on the length of the delay between the ROC and the first RDP session (Patients: M = 30; IQR = 196 days; CTRs: M = 30; IQR = 141.50 days; U = 36, *P* = 0.712).

#### Stimulus Materials

##### Faces

The 120 faces (*n* = 30 targets and *n* = 30 foils in each deadline condition) were front views of unknown Caucasian people, from the same Face Database as the first paradigm (Minear and Park 2004), but from different people (see above for more details). The faces used in the short deadline session did not differ from those in the long deadline session in either age (Short Deadline Session: M = 63.00; IQR = 45.75 years of age; Long Deadline Session: M = 61.00; IQR = 47.75 years of age; Short vs. Long Deadline Session: U = 1747.50, *P* = 0.785) or M:F ratio (Short Deadline Session: 20 M:40F; Long Deadline Session: 19 M:41F; Short vs. Long Deadline Session: χ^2^ = 0.038, *P* = 0.845). Targets and foils did not differ with respect to either age or M:F ratio in either the Short (age: targets vs. foils: U = 444.5, *P* = 0.939; *M*:*F* ratio: targets vs. foils: χ^2^ < 0.001, *P* > 0.999) or in the Long Deadline Session (age: targets vs. foils: U = 429, *P* = 0.761; M:F ratio: targets vs. foils: χ^2^ = 0.077, *P* = 0.781).

##### Scenes

The 120 scenes were pictures of natural landscapes (*n* = 30 targets and *n* = 30 foils per deadline condition), taken from the same source as the first paradigm (https://www.shutterstock.com), but different exemplars (see above for details). The Short deadline session did not differ from the Long deadline session with respect to the composition of the different themes across the scenes presented [Short deadline session: autumn (*n* = 3), beach (*n* = 4), clouds (*n* = 3), desert (*n* = 5), forest (*n* = 5), hills (*n* = 5), lakes (*n* = 4), mountains (*n* = 5), rocks (*n* = 7), rivers (*n* = 4), sea (*n* = 5), winter (*n* = 7), and waterfalls (*n* = 3); Long deadline session: autumn (*n* = 3), beach (*n* = 5), clouds (*n* = 2), desert (*n* = 5), forest (*n* = 4), hills (*n* = 5), lakes (*n* = 5), mountains (*n* = 4), rocks (*n* = 8), rivers (*n* = 4), sea (*n* = 5), winter (*n* = 7), and waterfalls (*n* = 3); Short vs. Long Deadline session: χ^2^ = 0.711, *P* > 0.999]. Likewise, no such differences were noted between target and foil items in either the Short (χ^2^ = 2.286, *P* > 0.999) or the Long Deadline session (χ^2^ = 1.810, *P* > 0.999).

##### Procedure

Stimulus presentation and data logging were programmed using the Psychophysics Toolbox (v.3) extensions (Brainard 1997; Pelli 1997; Kleiner et al. 2007). The session structure is presented in Supplementary Figure 3. The study phase of the paradigm involved 1 block per Material-Type (30 trials each). Participants were asked to rate each stimulus according to pleasantness (“Unpleasant,” “Neutral,” or “Pleasant”). They had 4.5 s to rate pleasantness of faces and scenes. In the test phase, participants were required to judge if the item presented on the screen was previously encountered in the study phase (pressing “1” for “Old”) or not (pressing “9” for “New”). The items were presented over 60 trials, broken down into 6 blocks of 10 trials with breaks after each block.

In each trial, a fixation cross was first presented, followed by the item, which was presented for either 400 ms (short response deadline) or 2000 ms (long response deadline). The participant was required to observe the item without responding. The item was then bordered in a blue square for 400 ms, during which time the participant was required to provide their response by pressing the “OLD” or the “NEW” button. An error noise was triggered for responses generated before the onset or after the offset of the response window.

For the same reasons as those described for the first paradigm, a series of blocks of filler trials were interspersed within the session, comprising 20 trials each, with a response window of 3 s per trial. Participants were presented with two numbers on the screen and were asked to select which number was the highest or the lowest. They pressed the “left” arrow to select the number presented on the left side of the display, and the “right” arrow for the number presented on the right side of the display. Participants pressed the “down” arrow to respond that the numbers were equal.

#### Behavioral Data Analysis

Signal detection theory was used to estimate the d’ measure of discriminability for each deadline, which was assumed to reflect pure familiarity in the short deadline, but a combination of recollection and familiarity in the long deadline, such that recollection can be estimated by subtracting the short deadline d’ from the long deadline d’ (Bowles et al. 2007).

### Statistical Analysis

Statistical analysis was performed using R (version 3.5.0) (R Core Team 2018). The scripts and raw data are available here: https://osf.io/a82ht/.

Of the 14 CTRs in total, two had missing values on the first paradigm and five had missing values on the second paradigm. These missing values were imputed using “Multiple Imputation with Chained Equations” implemented in the R function “mice.” Five imputations were created, and the range of *P*-values is reported across all five.

When comparing patients versus CTRs, we used Analysis of Variance (ANOVA) with within-participant factors Process (Recollection vs. Familiarity), Material-Type (Faces vs. Scenes), Paradigm (ROC vs. RDP), and a between-participant factor of Group (Patient vs. CTR). Only effects involving the Group factor are of interest (specifically, a main effect of Group or interactions involving Group, Process and/or Material-Type) and therefore reported in the main Results (for full ANOVA output see Supplementary Tables 2 and 3). In the presence of a significant interaction, follow-up ANOVAs were performed for each level of one of the interacting factors. An analogous ANOVA was used when comparing the two groups of patients against each other, except that the data were now each patient’s Z-score relative to CTRs, for which imputation was not required.

For the continuous analysis of brain–behavior relationships across all patients, the binary group factor was replaced with continuous measures of Z-scores for the volume of a candidate ROI (raw volume divided by TIV and then expressed as Z-scores) and entered into a linear mixed effects model (using “lmer” in R), with fully factorial fixed effects of ROI volume, Process, Material-Type, and Paradigm. This model was run on each of the 4 ROIs separately: HPC, PRC, ERC, and PHC, averaged across left and right hemispheres (see Supplementary Table 10 for results split by hemisphere). A single random intercept across participants was used (except when noted otherwise, additional random slopes did not always enable convergence, suggesting that a single error term was sufficiently parsimonious for our data; see https://arxiv.org/pdf/1506.04967.pdf). The results were reported in terms of Type III ANOVA using Satterthwaite’s method for adjusting degrees of freedom.

We also repeated our continuous analyses with age included as an additional covariate of no interest (Supplementary Table 6*b*). Moreover, in case our assumptions of Gaussian error terms were not met, *P*-values were also calculated from 5000 permutations using the “aovperm” (Supplementary Tables 2–4) and “lmperm” functions in R (Supplementary Table 7).

## Results

**Figure 2:**
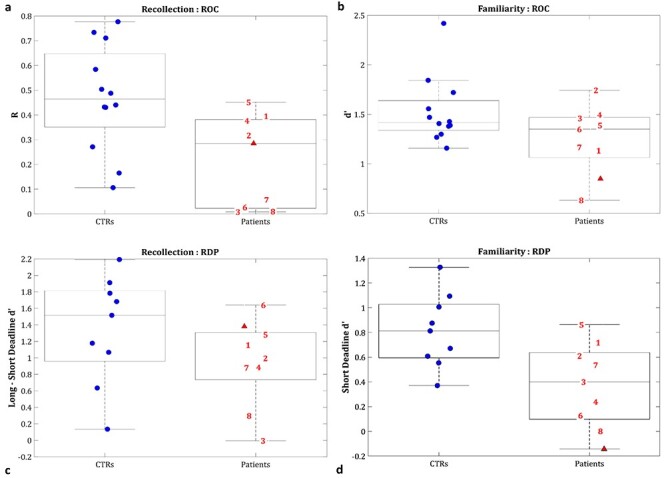
Recollection and familiarity estimates of CTRs and patients, averaging across Material-Types (Faces, Scenes) in the first paradigm (ROC; *a*, *b*) and in the second paradigm (RDP; *c*, *d*). The second boxplot in each panel pertains to all 9 patients; line in boxplots = median; bottom of box = 25th %ile; top = 75th %ile; whiskers = 1.5 ^*^ interquartile range; **key:** ▲ = MH (patient with right PRC lesion); **1–7** = patients with HPC but no PRC lesion; **8** = patient with both HPC and right PRC lesion.

We started with the more traditional categorical analysis of patients, based on a binary classification of whether or not the volume of various MTL structures fell below a threshold (1.67 SD below the mean of the CTR group). More specifically, we examined whether Recollection and/or Familiarity estimates, or the effects of Material-Type, differed 1) between the patient with a “selective” PRC lesion (MH) and CTRs and 2) between a group of patients with a “selective” HPC lesion (*n* = 7, after excluding H8, whose right PRC was also below the CTR cut-off) and CTRs. In order to test for a double-dissociation between these two types of patient, we then Z-transformed the patients’ behavioral scores on the basis of the mean and SD of the corresponding CTR data, and examined whether 3) the patient groups differed from each other in Recollection and/or Familiarity, or the effects of Material-Type. Finally, to examine continuous brain–behavior relationships, we ignored the binary classification into groups and combined all patients (*n* = 9) for a continuous analysis that related their Z-transformed volumes to their behavioral scores across tasks and material types, separately for the HPC, PRC, ERC, and PHC ROIs. Since analyses showed no significant effects of Material-Type, we averaged across this factor in order to show the main pattern in Figure 2; raw scores for CTRs and patients (split by Material-Type) are shown in Supplementary Figure 4.

## Categorical Analyses

### MH versus CTRs

To compare Patient MH with controls, an ANOVA was conducted on estimates of recollection and familiarity with within-participant factors of Process (Recollection vs. Familiarity), Material-Type (Faces vs. Scenes), Paradigm (ROC vs. RDP), and a between-participant factor of Group (PRC lesion, i.e., MH vs. CTRs). The only such effect to reach significance was the main effect of Group (0.041 ≥ *P* ≥ 0.017 across 5 imputations). While the Group-by-Process interaction did not quite reach significance (0.133 ≥ *P* ≥ 0.071), given prior claims of impairments on Familiarity rather than Recollection in a patient with lesions similar to MH (Bowles et al. 2007, 2010), we conducted follow-up ANOVAs on each type of memory separately. These showed a highly significant effect of Group on Familiarity (0.003 ≥ *P* ≥ 0.001), but not on Recollection (0.819 ≥ *P* ≥ 0.775; Table 1; for full ANOVA output, see Supplementary Table 2). Thus, our results were consistent with the previously reported patient NB (Bowles et al. 2007, 2010), suggesting that PRC damage impairs Familiarity but not Recollection.

### HPC Patients versus CTRs

Unlike the process-specific impairment that we observed above for patient MH, the ANOVA for the HPC group versus CTRs disclosed no suggestion of an interaction between Group and Process (0.874 ≥ *P* ≥ 0.649 across 5 imputations; Table 1; Supplementary Table 3), despite a clear main effect of Group (0.005 ≥ *P* ≥ 0.002), where the HPC group performed worse overall (as expected). Follow-up ANOVAs, to match those done for Patient MH, showed an effect of Group for Recollection (0.047 ≥ *P* ≥ 0.039) and, more surprisingly, also for Familiarity (0.015 ≥ *P* ≥ 0.004).

There was a borderline interaction between Group and Material-Type (0.102 ≥ *P* ≥ 0.018). Follow-up ANOVAs showed an effect of Group for Scenes (*P* ≤ 0.001), but only a marginal one for Faces (0.158 ≥ *P* ≥ 0.049).

To summarize, the main results from the comparison with CTRs were that the patient MH with PRC damage showed impaired familiarity but intact recollection (consistent with Bowles et al. 2010), whereas the patients with HPC damage were impaired on both recollection and familiarity. To explore this pattern further, and the possible additional effects of Material-Type, we next directly compared the two types of patient.

### MH versus HPC Patients

To test whether there was a double dissociation between the two categories of patient (PRC vs. HPC lesion), we added both MH and the 7 HPC cases (H1–7) to a single linear model, which was fit to their Z-scores relative to CTRs (so no imputation of missing CTR data was needed). This model included fixed effects of patient Group (PRC lesion vs. HPC lesion), Paradigm (ROC vs. RDP), Process (Recollection vs. Familiarity), and Material-Type (Faces vs. Scenes). The full results are shown in Supplementary Table 4. Here, we only report significant effects that involve the Group factor, since these reflect dissociations between the behavioral consequences of the two lesion locations. There was only one such effect: the two-way interaction between Group and Process (*F*(1,6) = 7.95, *P* = 0.030; Table 1).

Follow-up ANOVAs showed that the simple effect of Group was significant for Familiarity (*F*(1,6) = 8.50, *P* = 0.027), but not for Recollection (*F*(1,6) = 0.42, *P* = 0.542). When averaging across Material-Type and Paradigm, the mean Z-score for Recollection was −0.73 for the HPC group, but −0.33 for MH, whereas the mean Z-score for Familiarity was −0.57 for the HPC group, but −1.91 for MH. Numerically at least, this pattern of means shows a cross-over interaction in the degree of behavioral impairment, with MH relatively more impaired in Familiarity, and the HPC group relatively more impaired in Recollection.

**Table 1:** ANOVAs for the three planned, categorical analyses, with different between-participant definitions of Group [MH (PRC lesion) vs. CTRs, or HPC patients vs. CTRs, or MH vs. HPC patients]

Independent Variables per Model		Main Effects and Interactions	df1	df2	F		*P* (min)	*P* (max)
Within-participants	Group (between-participants)				Min	Max		
**Group, Process, Material-Type, Paradigm**	**MH, CTRs**	**Group**	1	13	5.12	7.46	0.017	0.041
		Group^*^Process	1	13	2.56	3.87	0.071	0.133
		Group^*^Material-Type	1	13	0.06	0.46	0.510	0.815
		Group^*^Material-Type^*^Process	1	13	<0.05	<0.05	0.840	0.996
	**HPC patients (H1–7), CTRs**	**Group**	1	19	10.06	13.66	0.002	0.005
		Group^*^Process	1	19	<0.05	0.21	0.649	0.874
		Group^*^Material-Type	1	19	2.95	6.71	0.018	0.102
		Group^*^Material-Type^*^Process	1	19	0.98	2.39	0.139	0.335
	**MH, HPC patients (H1–7)**	**Group**	1	6	1.12		0.330	
		Group^*^Process	1	6	7.95		0.030	
		Group^*^Material-Type	1	6	0.82		0.400	
		Group^*^Material-Type^*^Process	1	6	0.07		0.800	

Within-participants factors were Paradigm (ROC vs. RDP); Process (Familiarity vs. Recollection); and Material-Type (Faces vs. Scenes). Missing values were imputed using “Multiple Imputation with Chained Equations” implemented in the R function “mice.” Five imputations were created and we report the range of *F*- and *P*-values for the first two analyses. Only the effects of Group and its interactions with Material-Type and/or Process are of interest; for full results, see Supplementary Tables 2–4; **key: H1–7,** patients with HPC lesions without apparent PRC lesions; **MH,** patient with focal PRC lesion; **CTRs,** healthy controls.

## Continuous Analyses

Rather than binarizing patients according to whether specific brain regions are “lesioned” or “intact,” a potentially more powerful way to test models of functional specialization within the MTL is to correlate memory scores with continuous measures of the volume of ROIs (normalized by CTRs), across all patients. For this analysis, we combined MH with all 8 “HPC” patients (H1–H8; including now H8, the patient with additional right PRC lesion).

We estimated a linear mixed-effects model predicting Z-scored memory performance with fully factorial fixed effects of Process, Material-Type, and Paradigm, analogous to the categorical model above, except that the binary “Group” factor was replaced with the continuous Z-scored “ROI Volume” of the ROI tested (averaged across hemisphere; for analyses on left and right hemisphere only, see Supplementary Table 10). These models were conducted separately for each of the four ROIs (HPC, PRC, ERC, and PHC). As for the categorical analyses above, we only report significant effects that involve the ROI Volume factor, since these reflect brain–behavior relationships (Table 2; full results are reported in Supplementary Table 5).

**Table 2:** Continuous analyses; linear mixed-effects models (all patients; *n* = 9)

ROI model	Effect/Interaction	Df1	Df2	*F*	*P*
HPC	Volume	1	7	0.35	0.571
	Volume^*^Process	1	49	7.53	0.008
	Volume^*^Material-Type	1	49	0.30	0.589
	Volume^*^Process^*^Material-Type	1	49	0.10	0.750
PRC	Volume	1	7	0.72	0.425
	Volume^*^Process	1	49	0.08	0.784
	Volume^*^Material-Type	1	49	2.52	0.119
	Volume^*^Process^*^Material-Type	1	49	0.85	0.361
ERC	Volume	1	7	0.36	0.568
	Volume^*^Process	1	49	<0.05	0.927
	Volume^*^Material-Type	1	49	0.33	0.571
	Volume^*^Process^*^Material-Type	1	49	0.16	0.689
PHC	Volume	1	7	9.38	0.018
	Volume^*^Process	1	49	3.63	0.062
	Volume^*^Material-Type	1	49	0.25	0.616
	Volume^*^Process^*^Material-Type	1	49	0.41	0.527

Predictors: ROI Volume, Process (Recollection vs. Familiarity); Material-Type (Faces vs. Scenes), Paradigm (ROC vs. RDP) for each of the four ROIs (HPC, PRC, ERC, PHC), averaged across hemispheres. Only the effects of Volume and its interactions with Process and Material-Type are reported here; all effects and interactions are reported in Supplementary Table 7 (results for each hemisphere separately are reported in Supplementary Table 10). **ROI**, region of interest; **PHC**, parahippocampal cortex.

**Figure 3:**
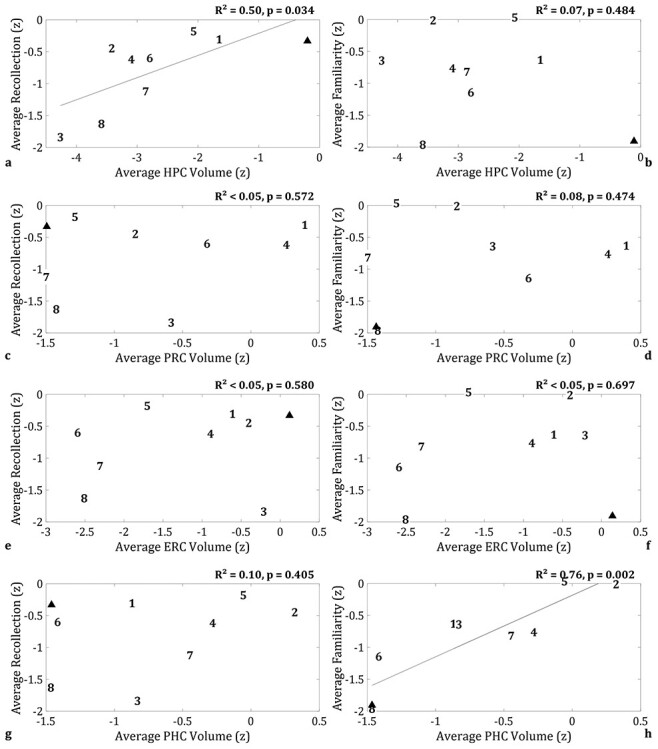
Double dissociation in brain-behavior relationships between HPC volume and Recollection, and PHC volume and Familiarity (averaged across Hemisphere, Material-Type and Paradigm); (*a*, *b*) average HPC volume correlated with Recollection, but not Familiarity; (*c*, *d*) average PRC volume did not correlate with recollection or familiarity; (*e*, *f*) average ERC volume did not correlate with recollection or familiarity; (*g*, *h*) average PHC volume correlated with Familiarity, but not Recollection. *R*^2^ values are reported for simple linear regressions. Note that effect size estimates are less precise when the sample size is small, so may be inflated (or deflated) here. **Key: PHC,** parahippocampal cortex; **Z,** volumes are expressed as Z-scores, based on the mean and standard deviation of the volumes of the 48 CTRs whose MTL structures were manually delineated (see Argyropoulos et al. 2019 for details); familiarity and recollection estimates are expressed as Z-scores, based on the mean and standard deviation of the CTRs that completed the two tasks (see also Supplementary Table 7).

For HPC, the only significant interaction was between ROI Volume and Process (*F*(1,49) = 7.53, *P* = 0.008). Follow-up analyses on Recollection and Familiarity separately (Supplementary Table 6*a*) showed an effect of ROI Volume on Recollection (*F*(1,7) = 6.93, *P* = 0.034), but not on Familiarity (*F*(1,7) = 0.55, *P* = 0.484). To illustrate this effect, Figure 3*a*,*b* shows scatter plots for Recollection and Familiarity, respectively, averaged across Material-Type and Paradigm (and hemisphere). The addition of Age in the model as a covariate did not affect the significance of the findings (Supplementary Table 6*b*).

For PRC, no interaction involving ROI Volume was significant (all *P*s, *P* ≥ 0.119; Table 2; Supplementary Table 5). As can be seen in Figure 3*c*,*d*, PRC volume did not show any significant relationship with Recollection or Familiarity.

ERC showed a similar pattern to ERC, that is, no significant interactions with ROI Volume (all *P*s, *P* ≥ 0.571; Table 2; Supplementary Table 5) and no relationship with Recollection or Familiarity (Fig. 3*e*,*f*).

For PHC, the only interaction that approached significance was between ROI Volume and Process (*F*(1,49) = 3.63, *P* = 0.062; Table 2; Supplementary Table 5). Given the expected direction of a larger effect on Familiarity than Recollection, and for comparison with HPC, we analyzed Recollection and Familiarity separately (Supplementary Table 6*a*). These showed no effect of ROI Volume on Recollection (*F*(1,7) = 0.79, *P* = 0.405), but a highly significant effect on Familiarity (*F*(1,28) = 14.09, *P* = 0.001). This model was estimated as singular by the “lmer” function, so it was repeated with a more complex error structure, namely adding random slopes for Material-Type and Paradigm. This model was no longer singular, and the effect of ROI volume remained significant (*F*(1,7.9) = 21.6, *P* = 0.002). This dissociation is illustrated in Figure 3*g*,*h*. These effects remained even with age as an additional fixed effect (Supplementary Table 6*b*).

## Discussion

In this study, we examined the relationship between MTL damage and impairment in recognition memory for two types of memoranda (unknown faces, topographical scenes) within two distinct paradigms designed to separate recollection and familiarity processes. Across a cohort of 9 patients with MTL damage, we observed that recollection impairment was associated with degree of HPC volume reduction, whereas familiarity impairment was associated with degree of PHC volume reduction. There was no evidence that these relationships depended on material type. When using a more traditional “categorical” analysis, in which patients were categorized according to which brain region was appreciably below the control mean, a somewhat different pattern emerged, in which HPC lesions were associated with impairments of both recollection and familiarity, and also greater impairments for scenes than faces, whereas the one case with a selective PRC lesion was associated with impairments of familiarity (with no effect of material-type; note that no patient had PHC volume below our threshold for defining a lesion). We discuss possible explanations for these different results below but, more generally, both analyses provide compelling neuropsychological support for dual-process models of recognition memory, whereby recollection and familiarity depend on the integrity of distinct MTL structures.

### Support for Dual-Process Models

There is an ongoing debate whether different structures within the MTL support distinct processes underlying recognition memory, especially whether the HPC selectively supports recollection and whether the PRC and/or PHC selectively support familiarity (e.g., Aggleton and Brown 1999; Montaldi and Mayes 2011; but see Squire et al. 2004; Wixted and Squire 2011b). Whereas task-based fMRI (e.g., Staresina et al. 2012; Staresina, Cooper, et al. 2013; Kafkas et al. 2016) and intracranial EEG studies (e.g., Staresina, Fell, et al. 2013) have provided some evidence for such dissociations, patient studies are required to establish causal brain–behavior relationships. Nevertheless, patient studies are limited by the scarcity of patients with “focal” PRC lesions. To our knowledge, only one such study has provided evidence for a double dissociation between recollection and familiarity (Bowles et al. 2010). This study reported on patient NB, who had a lesion in the left PRC that extended to the amygdala, ERC and anterolateral temporal cortices, and who presented with a selective familiarity impairment (Bowles et al. 2007). Importantly, she showed the opposite pattern to an older patient who had undergone left amygdalo-hippocampectomy and who presented with a selective recollection impairment (demonstrated with a “Remember-Know” paradigm), despite their comparable overall recognition memory performance (Bowles et al. 2010).

Our PRC patient, MH, appears to have a more focal lesion than patient NB. Consistent with NB’s performance (Köhler and Martin 2020), our patient showed impaired familiarity and preserved recollection relative to CTRs. Moreover, when compared directly with a larger group of amnesic patients, who have HPC lesions appearing to spare the PRC, MH showed greater impairment on familiarity and less on recollection. Nevertheless, our HPC group did show impaired familiarity relative to CTRs, in apparent conflict with dual-process theories and previous reports of impaired recollection but intact familiarity following HPC lesions (e.g., Aggleton et al. 2005; Vann et al. 2009).

Prima facie, this finding might be considered to support single-process theories (e.g., Wixted and Squire 2011a), aligning with other reported cases in which HPC lesions are accompanied by impairment of both recollection and familiarity (e.g., Wais et al. 2006; Kirwan et al. 2010; Song et al. 2011). Nonetheless, the latter interpretation was questioned by our subsequent continuous analyses. Moving beyond the dichotomization of structures into “lesioned” and “preserved,” we examined whether the variability in familiarity and recollection was a function of the volume of different MTL structures across patients. This approach is consistent with the idea that damage within one MTL structure often co-occurs with structural and/or functional abnormalities in broader networks of regions (Henson et al. 2016; Bubb et al. 2017; Argyropoulos et al. 2019), which might be particularly likely for etiologies such as the autoimmune limbic encephalitis in the case of our HPC patients (Argyropoulos et al. 2019; Loane et al. 2019). For the continuous analyses, there was compelling evidence for a selective role of HPC in recollection, since HPC volume correlated with recollection but not with familiarity. The impairments in familiarity in the “HPC group” (in the categorical analysis) are therefore most likely due to subthreshold damage to other regions outside the HPC, such as the PHC (see below). Indeed, the correlation between HPC volume and recollection was mirrored by a correlation between PHC volume and familiarity (but not of PHC volume with recollection). This double-dissociation of HPC correlation with recollection but not familiarity, and PHC correlation with familiarity but not recollection, is consistent with the large body of evidence from other studies in favor of “dual-process” theories, particularly those that map these processes to distinct regions with the MTL (e.g., Aggleton and Brown 2006; Montaldi and Mayes 2010).

### Possible Effects of Material-Type

Some results from our categorical analyses might be interpreted as supporting a representational account of MTL function (e.g., Graham et al. 2010; Ranganath 2010; Nadel and Peterson 2013; Lacot et al. 2017) or, more specifically, the view that different parahippocampal structures support familiarity for different material-types (Kafkas et al. 2016; Köhler and Martin 2020). In particular, both MH and H8 (who had a lesion in the right PRC: volume: *Z* < −1.67) showed impaired face recognition memory in neuropsychological assessment, along with impaired face familiarity relative to the rest of the patients. At group level, patients with HPC lesions (H1–7) showed only marginal impairment in memory for faces, in contrast with marked impairment for scenes (Supplementary Table 3). This pattern dovetails with evidence from case studies of patients with HPC damage (e.g., Cipolotti et al. 2006), meta-analytical findings (Bird and Burgess 2008) on HPC patients’ performance in neuropsychological tests of recognition memory for faces, and with our previous findings on a larger cohort of HPC patients, who showed group-level sparing of face recognition (Argyropoulos et al. 2019). It also supports the idea that the PRC is engaged in processing faces (see Robin et al. 2019 for discussion).

Nevertheless, MH’s familiarity impairment was not selective for faces, and he did not show process-independent or material-specific impairment relative to CTRs. Furthermore, our continuous analyses did not detect any evidence for a privileged role of the PHC (e.g., Buffalo et al. 2006; Litman et al. 2009) or the HPC (e.g., Mullally et al. 2014) in processing spatial information (here, in the form of unknown topographical scenes). Thus, unlike our clear evidence for dissociations between MTL regions as a function of memory process, our results do not fully disentangle potential further dissociations as a function of material-type. Future research with larger patient cohorts (and hence greater power for detecting continuous relationships) may be needed to investigate interactions between memory processes, material-types, and MTL regions.

### PHC and Familiarity

In neuropsychological dual-process frameworks, the PRC is the portion of the parahippocampal gyrus that has been most consistently associated with familiarity processes (e.g., Brown and Aggleton 2001; Köhler and Martin 2020). In these frameworks, the PHC has more commonly been associated with processing context information, including its recollection (e.g., the “Binding in Context” model and subsequent developments; Diana et al. 2007; Ranganath 2010) or familiarity (e.g., Montaldi and Mayes 2010). However, there is also, in our opinion, a somewhat neglected literature on the role of the PHC in processing item familiarity. In task-based fMRI studies in healthy participants, the PHC has shown increased activation during judgments of pre-existing familiarity (Gimbel et al. 2017), sensitivity to perceptual and task novelty/familiarity (O’Kane et al. 2005), and material-specific familiarity-related signals (Martin et al. 2013, 2018). In single-neuron recordings in presurgical epilepsy patients, a large portion of familiarity-related neurons was found in the PHC, with a substantial portion of all PHC neurons related to successful item retrieval (Derner et al. 2020, though no direct comparison could be made to the PRC, since no PRC neurons were evaluated). In another study, PHC neurons were more likely than those in other MTL regions to respond to familiar face/scene pairs and, in contrast to HPC neurons, were not sensitive to recombining face/scene pairs (Viskontas et al. 2016).

Most of these previous studies are based on brain–behavior correlations in healthy adults. It is possible, therefore, that PHC contributes to both recollection and familiarity in the healthy brain but is only “indispensable” for familiarity. In other words, the role of these regions in the healthy brain may differ from their role in a damaged brain, owing for example to the disruption of connectivity between brain regions (Henson et al. 2016).

Overall, our findings here should be seen not as competing but as complementary with respect to PRC and PHC contributions to familiarity processes underlying recognition memory. The absence of evidence from our continuous analysis for a PRC role in familiarity should not be interpreted as evidence of absence for such a role.

### Beyond the Case-Study Approach

An important methodological message from our findings is the benefit of moving beyond the case-study approach, which requires the dichotomization of structures into “lesioned” (e.g., volumes below some threshold) and “preserved.” By testing a number of patients, we capitalized on the variability in the integrity of the different structures to examine continuous brain–behavior relationships, allowing for individual differences and obviating the need for arbitrary thresholds to define “lesioned vs. nonlesioned” (Lambon Ralph et al. 2011). The danger of the dichotomous approach to characterizing MTL lesions is illustrated by our examination of the HPC group, who showed evidence of familiarity impairments even though there was no evidence that HPC volume correlated with familiarity. We interpret this as indicating that their HPC lesion caused their recollection impairment but that subthreshold damage to other MTL regions (e.g., PHC) caused their familiarity impairment. Group-based, continuous analyses like those performed here might help resolve debates in the domain of MTL amnesia that may have arisen largely from the focus on single-case studies (e.g., Aggleton and Brown 1999; Montaldi and Mayes 2011; but see Squire et al. 2004; Wixted and Squire 2011b).

Moreover, unlike our categorical analyses, there was less concern regarding low degrees of freedom (dfs) in our continuous analyses, since we did not focus our inferences upon single correlations with *N* = 9 datapoints (i.e., 8 dfs). Instead, we combined Material-Type (Faces/Scenes), Paradigm (ROC/RDP), and Process (Recollection/Familiarity) into a single linear model, greatly increasing our residual dfs. By not splitting these data into small subsets, we gained power by averaging over, or interacting across, multiple factors.

Moreover, we note that, just because one finds a correlation between regional volume and behavior in patients, this does not imply that one should see the same brain–behavior correlation in healthy individuals. Indeed, we saw no trends for such structure–behavior relationships in our CTRs (though our power was low; Supplementary Fig. 5). We think this is because the regional volumes in patients are likely to reflect a within-participant change in volume, relative to their premorbid volume (which is likely to be close to the mean of controls). Behavioral performance is more likely to reflect such brain “changes,” rather than stable individual (cross-sectional) differences in brain volumes (analogous to the different results often seen between longitudinal and cross-sectional studies; Raz and Lindenberger 2011).

### Limitations and Future Directions

There are certain limitations to our study. First, in both paradigms, the order of presentation of the stimulus blocks (faces, scenes, words) was kept constant across participants. This enabled us to compare directly the performance of MH with that of CTRs and HPC patients. (Since a single case such as MH can typically only attempt one order of tasks, at least when it is difficult to repeat those tasks on the same person; another advantage of group studies is that task order is more easily counter-balanced.)

Note that, due to technical errors in the design of the second paradigm (RDP), the word stimuli in the second session (short response deadline) were the same as those used in the first session (long response deadline). Because this could potentially affect familiarity or recollection differentially for patients versus CTRs, we excluded the word stimuli from the present analyses (but report them in Supplementary Tables 11–19 for completeness). As a result, we were unable to assess whether our patient’s familiarity deficit was selective for nonverbal material, given the evidence for a relationship between left PRC and familiarity for phonological and conceptual aspects of verbal material (Martin et al. 2011; Köhler and Martin 2020).

Another issue that our study did not address is the possibility that the extent to which scenes are processed by the HPC, PHC, or PRC is a function of stimulus size. It has been argued that relatively small stimuli may be treated like objects, thus maximizing the involvement of the PRC, whereas processing larger background stimuli requires an intact PHC (Cassaday and Rawlins 1997; Montaldi and Mayes 2010). We thus cannot exclude the possibility that MH’s recognition memory for scenes was affected by the relatively small size of our scene stimuli. These limitations are another reason why the present study cannot offer definitive evidence on the role of different material-types.

Moreover, the nature of our cohort means that we had little power to detect differential effects of bilateral versus unilateral MTL damage on recognition memory. This is primarily because all 8 of our HPC patients showed largely bilateral HPC damage (L and R HPC: *Z* < −1.4), which is the characteristic of the etiology (Argyropoulos et al. 2019). Indeed, bilateral HPC damage may be necessary to see recollection deficits (see Spiers et al. 2001 for discussion). Interestingly however, while Patient MH’s PRC lesion was clearly unilateral (R PRC: *Z* = −2.99; L PRC: *Z* = 0.12), his PHC was the only MTL structure that showed bilateral volume reduction (R and L PHC: *Z* < −1.2). It is conceivable that the latter might be the cause of his deficits in familiarity (rather than the unilateral PRC lesion that initially brought him to our attention).

Larger patient groups are needed to ensure sufficient variability in both the extent and the laterality of MTL damage, in order to replicate the novel relationship noted here between PHC volume and familiarity. Indeed, some of the effect sizes we report may be imprecise owing to the small sample size (e.g., possibly inflated in the case of *R*^2^ > 0.7). Ideally, such larger patient cohorts would also be etiologically more homogeneous than the cohort here, which comprised 8 patients that had suffered from autoimmune limbic encephalitis, along with one (MH) who had damage due to a previous focal abscess in the R PRC.

Finally, the present study did not examine other brain abnormalities that may be associated with MTL damage, such as lesion-induced changes in functional activity or structural and functional connectivity. Thus, whether the effects of MTL damage on recollection and familiarity that we noted here are better explained by abnormalities in broader functional networks involving MTL regions (Argyropoulos et al. 2019) needs further investigation (we did not have fMRI data on all present cases). Likewise, our data do not suffice to ascertain whether the relationship between PHC damage and familiarity impairment might be explained by damage to fibers passing through the PHC to the PRC (Lavenex et al. 2004). In the same vein, although we did not observe any relationship between overall ERC volumes and recollection strength, manual delineation of the anterolateral and the posteromedial portions of the ERC would be required for closer investigation of potential relationships between damage in these and process- or material-specific recognition memory impairment (e.g., Sauvage et al. 2010; Schultz et al. 2012). Moreover, our scanning protocols did not allow us to investigate the integrity of smaller structures within the Papez circuit, which have also been implicated in memory, such as the thalamic nuclei and mammillary bodies (Aggleton and Brown 1999; Tsivilis et al. 2008; Kafkas et al. 2020).

## Conclusion

We believe that our data provide the most compelling neuropsychological support yet for dual-process models of recognition memory, in which recollection and familiarity depend on different MTL structures. By capitalizing on the variability of damage across patients with MTL pathology, our study complements single-case approaches and suggests that the PHC may be necessary for familiarity. Future studies of even larger patient groups, ideally across centers and using multiple, common paradigms and material-types, will hopefully further dissect the contributions of different MTL regions to memory.

## Supplementary Material

argyropoulos_et_al_cercor_supplementary_material_03_07_2021_bhab290

## Data Availability

Scripts for statistical analysis, behavioral and volumetric data are publicly available at: https://osf.io/a82ht

## Notes

We are very grateful to the participants who took part in this study. *Conflict of Interests:* The authors declare no competing financial interests.

## References

[ref1] Aggleton JP , Brown MW. 2006. Interleaving brain systems for episodic and recognition memory. Trends Cogn Sci. 10:455–463. 10.1016/j.tics.2006.08.003. 16935547

[ref2] Aggleton JP , Brown MW. 1999. Episodic memory, amnesia, and the hippocampal–anterior thalamic axis. Behav Brain Sci. 22:425–444. 10.1017/S0140525X99002034. 11301518

[ref3] Aggleton JP , Vann SD, Denby C, Dix S, Mayes AR, Roberts N, Yonelinas AP. 2005. Sparing of the familiarity component of recognition memory in a patient with hippocampal pathology. Neuropsychologia. 43:1810–1823. 10.1016/j.neuropsychologia.2005.01.019. 16154457

[ref4] Argyropoulos GP , Loane C, Roca-Fernandez A, Lage-Martinez C, Gurau O, Irani SR, Butler CR. 2019. Network-wide abnormalities explain memory variability in hippocampal amnesia. Elife. 8:e46156. 10.7554/eLife.46156. PMC6639076 31282861

[ref5] Argyropoulos GPD , Butler CR. 2020. Does hippocampal atrophy explain anterograde and retrograde amnesia following autoimmune limbic encephalitis? Hippocampus. 30:1013–1017. 10.1002/hipo.23208. 32320116

[ref6] Ashburner J . 2007. A fast diffeomorphic image registration algorithm. Neuroimage. 38:95–113. 10.1016/j.neuroimage.2007.07.007. 17761438

[ref7] Baddeley A , Emslie H, Nimmo-Smith I. 1994. Doors and people: a test of visual and verbal recall and recognition. Bury St. Edmunds, England: Thames Valley Test Co.

[ref8] Bird CM , Burgess N. 2008. The hippocampus supports recognition memory for familiar words but not unfamiliar faces. Curr Biol. 18:1932–1936. 10.1016/j.cub.2008.10.046. 19084409

[ref9] Bowles B , Crupi C, Mirsattari SM, Pigott SE, Parrent AG, Pruessner JC, Yonelinas AP, Kohler S. 2007. Impaired familiarity with preserved recollection after anterior temporal-lobe resection that spares the hippocampus. Proc Natl Acad Sci. 104:16382–16387. 10.1073/pnas.0705273104. 17905870 PMC1995093

[ref10] Bowles B , Crupi C, Pigott S, Parrent A, Wiebe S, Janzen L, Köhler S. 2010. Double dissociation of selective recollection and familiarity impairments following two different surgical treatments for temporal-lobe epilepsy. Neuropsychologia. 48:2640–2647. 10.1016/J.NEUROPSYCHOLOGIA.2010.05.010. 20466009

[ref11] Bozeat S , Lambon Ralph MA, Patterson K, Garrard P, Hodges JR. 2000. Non-verbal semantic impairment in semantic dementia. Neuropsychologia. 38:1207–1215. 10.1016/S0028-3932(00)00034-8. 10865096

[ref12] Brainard DH . 1997. The psychophysics toolbox. Spat Vis. 10:433–436. 10.1163/156856897X00357. 9176952

[ref13] Brown MW , Aggleton JP. 2001. Recognition memory: what are the roles of the perirhinal cortex and hippocampus? Nat Rev Neurosci. 2:51–61. 10.1038/35049064. 11253359

[ref14] Bubb EJ , Kinnavane L, Aggleton JP. 2017. Hippocampal–diencephalic–cingulate networks for memory and emotion: an anatomical guide. Brain Neurosci Adv. 1:239821281772344. 10.1177/2398212817723443. PMC5608081 28944298

[ref15] Buffalo EA , Bellgowan PSF, Martin A. 2006. Distinct roles for medial temporal lobe structures in memory for objects and their locations. Learn Mem. 13:638–643. 10.1101/lm.251906. 16980544 PMC1783618

[ref16] Butler CR , Miller TD, Kaur MS, Baker IW, Boothroyd GD, Illman NA, Rosenthal CR, Vincent A, Buckley CJ. 2014. Persistent anterograde amnesia following limbic encephalitis associated with antibodies to the voltage-gated potassium channel complex. J Neurol Neurosurg Psychiatry. 85:387–391. 10.1136/jnnp-2013-306724. 24403282

[ref17] Cassaday HJ , Rawlins JNP. 1997. The hippocampus, objects, and their contexts. Behav Neurosci. 111:1228–1244. 10.1037/0735-7044.111.6.1228. 9438792

[ref18] Cipolotti L , Bird C, Good T, Macmanus D, Rudge P, Shallice T. 2006. Recollection and familiarity in dense hippocampal amnesia: a case study. Neuropsychologia. 44:489–506. 10.1016/j.neuropsychologia.2005.05.014. 16023686

[ref19] Delis DCD , Kaplan E, Kramer JH. 2001. The Delis-Kaplan Executive Function System. 2nd ed. San Antonio, TX: The Psychological Corporation.

[ref20] Derner M , Dehnen G, Chaieb L, Reber TP, Borger V, Surges R, Staresina BP, Mormann F, Fell J. 2020. Patterns of single-neuron activity during associative recognition memory in the human medial temporal lobe. Neuroimage. 221:117214. 10.1016/j.neuroimage.2020.117214. 32755669

[ref21] Diana RA , Yonelinas AP, Ranganath C. 2007. Imaging recollection and familiarity in the medial temporal lobe: a three-component model. Trends Cogn Sci. 11:379–386. 10.1016/j.tics.2007.08.001. 17707683

[ref22] Gimbel SI , Brewer JB, Maril A. 2017. I know I’ve seen you before: distinguishing recent-single-exposure-based familiarity from pre-existing familiarity. Brain Res. 1658:11–24. 10.1016/j.brainres.2017.01.007. 28073651 PMC5867277

[ref23] Graham KS , Barense MD, Lee ACHH. 2010. Going beyond LTM in the MTL: a synthesis of neuropsychological and neuroimaging findings on the role of the medial temporal lobe in memory and perception. Neuropsychologia. 48:831–853. 10.1016/j.neuropsychologia.2010.01.001. 20074580

[ref24] Graus F , Titulaer MJ, Balu R, Benseler S, Bien CG, Cellucci T, Cortese I, Dale RC, Gelfand JM, Geschwind M, et al. 2016. A clinical approach to diagnosis of autoimmune encephalitis. Lancet Neurol. 15:391–404. 10.1016/S1474-4422(15)00401-9. 26906964 PMC5066574

[ref25] Henson RN , Greve A, Cooper E, Gregori M, Simons JS, Geerligs L, Erzinçlioğlu S, Kapur N, Browne G, Geerligs L, et al. 2016. The effects of hippocampal lesions on MRI measures of structural and functional connectivity. Hippocampus. 26:1447–1463. 10.1002/hipo.22621. 27479794 PMC5082505

[ref26] Huang BY , Castillo M. 2008. Hypoxic-ischemic brain injury: imaging findings from birth to adulthood. Radiographics. 28:417–439. 10.1148/rg.282075066. 18349449

[ref27] Inhoff MC , Heusser AC, Tambini A, Martin CB, O’Neil EB, Köhler S, Meager MR, Blackmon K, Vazquez B, Devinsky O, et al. 2019. Understanding perirhinal contributions to perception and memory: evidence through the lens of selective perirhinal damage. Neuropsychologia. 124:9–18. 10.1016/J.NEUROPSYCHOLOGIA.2018.12.020. 30594569 PMC6456260

[ref28] Insausti R , Juottonen K, Soininen H, Insausti AM, Partanen K, Vainio P, Laakso MP, Pitkänen A. 1998. MR volumetric analysis of the human entorhinal, perirhinal, and temporopolar cortices. Am J Neuroradiol. 19:659–671. 9576651 PMC8337393

[ref29] Kafkas A , Mayes AR, Montaldi D. 2020. Thalamic-medial temporal lobe connectivity underpins familiarity memory. Cereb Cortex. 30:3827–3837. 10.1093/cercor/bhz345. 31989161 PMC7232995

[ref30] Kafkas A , Migo EM, Morris RG, Kopelman MD, Montaldi D, Mayes AR. 2016. Material specificity drives medial temporal lobe familiarity but not hippocampal recollection. Hippocampus. 209:1–20. 10.1002/hipo.22683. PMC5299537 27859925

[ref31] Kim S , Dede AJO, Hopkins RO, Squire LR. 2015. Memory, scene construction, and the human hippocampus. Proc Natl Acad Sci U S A. 112:4767–4772. 10.1073/pnas 25825712 PMC4403152

[ref32] Kirwan CB , Wixted JT, Squire LR. 2010. A demonstration that the hippocampus supports both recollection and familiarity. Proc Natl Acad Sci U S A. 107:344–348. 10.1073/pnas.0912543107. 19966306 PMC2806702

[ref33] Kleiner M, Brainard D, Pelli D, Ingling A, Murray R, Broussard C. 2007. What's new in Psychtoolbox-3. Perception 36:1.1–16.

[ref34] Köhler S , Martin CB. 2020. Familiarity impairments after anterior temporal-lobe resection with hippocampal sparing: lessons learned from case NB. Neuropsychologia. 138:107339. 10.1016/J.NEUROPSYCHOLOGIA.2020.107339. 31930957

[ref35] Lacot E , Vautier S, Kőhler S, Pariente J, Martin CB, Puel M, Lotterie J-AA, Barbeau EJ. 2017. Familiarity and recollection vs representational models of medial temporal lobe structures: a single-case study. Neuropsychologia. 104:76–91. 10.1016/j.neuropsychologia.2017.07.032. 28760565

[ref36] Lambon Ralph MA , Patterson K, Plaut DC. 2011. Finite case series or infinite single-case studies? Comments on “case series investigations in cognitive neuropsychology” by Schwartz and Dell (2010). Cogn Neuropsychol. 28:466–474. 10.1080/02643294.2012.671765. 22746688

[ref37] Lavenex P , Suzuki WA, Amaral DG. 2004. Perirhinal and parahippocampal cortices of the macaque monkey: intrinsic projections and interconnections. J Comp Neurol. 472:371–394. 10.1002/cne.20079. 15065131

[ref38] Litman L , Awipi T, Davachi L. 2009. Category-specificity in the human medial temporal lobe cortex. Hippocampus. 19:308–319. 10.1002/hipo.20515. 18988234 PMC2649983

[ref39] Loane C , Argyropoulos GPDD, Roca-Fernández A, Lage C, Sheerin F, Ahmed S, Zamboni G, MacKay C, Irani SR, Butler CR. 2019. Hippocampal network abnormalities explain amnesia after VGKCC-ab related autoimmune limbic encephalitis. J Neurol Neurosurg Psychiatry. 90:965–974. 10.1136/jnnp-2018-320168. 31072956 PMC6820158

[ref40] Maass A , Berron D, Libby LA, Ranganath C, Düzel E. 2015. Functional subregions of the human entorhinal cortex. Elife. 4:1–20. 10.7554/eLife.06426. PMC4458841 26052749

[ref41] Martin CB , Bowles B, Mirsattari SM, Köhler S. 2011. Selective familiarity deficits after left anterior temporal-lobe removal with hippocampal sparing are material specific. Neuropsychologia. 49:1870–1878. 10.1016/j.neuropsychologia.2011.03.012. 21419788

[ref42] Martin CB , McLean DA, O’Neil EB, Köhler S. 2013. Distinct familiarity-based response patterns for faces and buildings in perirhinal and parahippocampal cortex. J Neurosci. 33:10915–10923. 10.1523/JNEUROSCI.0126-13.2013. 23804111 PMC6618503

[ref43] Martin CB , Sullivan JA, Wright J, Köhler S. 2018. How landmark suitability shapes recognition memory signals for objects in the medial temporal lobes. Neuroimage. 166:425–436. 10.1016/j.neuroimage.2017.11.004. 29108942

[ref44] Mckenna P , Warrington EK. 1980. Testing for nominal dysphasia. J Neurol Neurosurg Psychiatry. 43:781–788. 10.1136/jnnp.43.9.781. 7420102 PMC490668

[ref45] Minear M , Park DC. 2004. A lifespan database of adult facial stimuli. Behav Res Methods Instrum Comput. 36:630–633. 10.3758/BF03206543. 15641408

[ref46] Montaldi D , Mayes AR. 2011. Familiarity, recollection and medial temporal lobe function: an unresolved issue. Trends Cogn Sci. 15:339–340. 10.1016/j.tics.2011.06.007. 21752696

[ref47] Montaldi D , Mayes AR. 2010. The role of recollection and familiarity in the functional differentiation of the medial temporal lobes. Hippocampus. 20:1291–1314. 10.1002/hipo.20853. 20928828

[ref48] Mullally SL , Vargha-Khadem F, Maguire EA. 2014. Scene construction in developmental amnesia: an fMRI study. Neuropsychologia. 52:1–10. 10.1016/j.neuropsychologia.2013.11.001. 24231038 PMC3905188

[ref49] Nadel L , Peterson MA. 2013. The hippocampus: part of an interactive posterior representational system spanning perceptual and memorial systems. J Exp Psychol Gen. 142:1242–1254. 10.1037/a0033690. 23895347

[ref50] Nelson HE , Willison J. 1991. National Adult Reading Test (NART): Test manual. Windsor, England: NFER-Nelson.

[ref51] O’Kane G , Insler RZ, Wagner AD. 2005. Conceptual and perceptual novelty effects in human medial temporal cortex. Hippocampus. 15:326–332. 10.1002/hipo.20053. 15490462

[ref52] Pelli DG . 1997. The VideoToolbox software for visual psychophysics: transforming numbers into movies. Spat Vis. 10:437–442. 10.1163/156856897X00366. 9176953

[ref53] Pruessner JC , Köhler S, Crane J, Pruessner M, Lord C, Byrne A, Kabani N, Collins DL, Evans AC. 2002. Volumetry of temporopolar, perirhinal, entorhinal and parahippocampal cortex from high-resolution MR images: considering the variability of the collateral sulcus. Cereb Cortex. 12:1342–1353. 10.1093/cercor/12.12.1342. 12427684

[ref54] Pustina D , Gizewski E, Forsting M, Daum I, Suchan B. 2012. Human memory manipulated: dissociating factors contributing to MTL activity, an fMRI study. Behav Brain Res. 229:57–67. 10.1016/J.BBR.2011.12.034. 22230115

[ref55] R Core Team . 2018. R: A language and environment for statistical computing. Vienna: R Foundation for Statistical Computing.

[ref56] Ranganath C . 2010. A unified framework for the functional organization of the medial temporal lobes and the phenomenology of episodic memory. Hippocampus. 20:1263–1290. 10.1002/hipo.20852. 20928833

[ref57] Raz N , Lindenberger U. 2011. Only time will tell: cross-sectional studies offer no solution to the age–brain–cognition triangle: comment on Salthouse (2011). Psychol Bull. 137:790–795. 10.1037/a0024503. 21859179 PMC3160731

[ref58] Reagh ZM , Yassa MA. 2014. Object and spatial mnemonic interference differentially engage lateral and medial entorhinal cortex in humans. Proc Natl Acad Sci U S A. 111:E4264–E4273. 10.1073/pnas.1411250111. 25246569 PMC4210036

[ref59] Rey A. 1959. Manuel du test de copie d’une figure complexe de A. Rey. Paris Les Ed. du Cent. Psychol. Appliquée.

[ref60] Robin J , Rai Y, Valli M, Olsen RK. 2019. Category specificity in the medial temporal lobe: a systematic review. Hippocampus. 29:313–339. 10.1002/hipo.23024. 30155943

[ref61] Sauvage MM , Beer Z, Ekovich M, Ho L, Eichenbaum H. 2010. The caudal medial entorhinal cortex: a selective role in recollection-based recognition memory. J Neurosci. 30:15695–15699. 10.1523/JNEUROSCI.4301-10.2010. 21084625 PMC3073554

[ref62] Schultz H , Sommer T, Peters J. 2012. Direct evidence for domain-sensitive functional subregions in human entorhinal cortex. J Neurosci. 32:4716–4723. 10.1523/JNEUROSCI.5126-11.2012. 22492028 PMC6620910

[ref63] Scoville WB , Milner B. 1957. Loss of recentmemory after bilateral hippocampal lesions. J Neurol Neurosurg Psychiatry. 20:11–21. 10.1136/jnnp.20.1.11. 13406589 PMC497229

[ref64] Shallice T. 2019. The single case study of memory. In: Cases of amnesia. New York: Routledge, pp. 1–15. 10.4324/9780429023880-1

[ref65] Song Z , Jeneson A, Squire LR. 2011. Medial temporal lobe function and recognition memory: a novel approach to separating the contribution of recollection and familiarity. J Neurosci. 31:16026–16032. 10.1523/JNEUROSCI.3012-11.2011. 22049444 PMC3227550

[ref66] Spiers HJ , Maguire EA, Burgess N. 2001. Hippocampal amnesia. Neurocase. 7:357–382. 10.1076/neur.7.5.357.16245. 11744778

[ref67] Squire LR , Stark CEL, Clark RE. 2004. The medial temporal lobe. Annu Rev Neurosci. 27:279–306. 10.1146/annurev.neuro.27.070203.144130. 15217334

[ref68] Squire LR , Wixted JT. 2011. The cognitive neuroscience of human memory since H.M. Annu Rev Neurosci. 34:259–288. 10.1146/annurev-neuro-061010-113720. 21456960 PMC3192650

[ref69] Staresina BP , Cooper E, Henson RN. 2013. Reversible information flow across the medial temporal lobe: the hippocampus links cortical modules during memory retrieval. J Neurosci. 33:14184–14192. 10.1523/JNEUROSCI.1987-13.2013. 23986252 PMC3756762

[ref70] Staresina BP , Fell J, Do Lam ATA, Axmacher N, Henson RN. 2012. Memory signals are temporally dissociated in and across human hippocampus and perirhinal cortex. Nat Neurosci. 15:1167–1173. 10.1038/nn.3154. 22751037 PMC3428860

[ref71] Staresina BP , Fell J, Dunn JC, Axmacher N, Henson RN. 2013. Using state-trace analysis to dissociate the functions of the human hippocampus and perirhinal cortex in recognition memory. Proc Natl Acad Sci U S A. 110:3119–3124. 10.1073/pnas.1215710110. 23382181 PMC3581882

[ref72] Tsivilis D , Vann SD, Denby C, Roberts N, Mayes AR, Montaldi D, Aggleton JP. 2008. A disproportionate role for the fornix and mammillary bodies in recall versus recognition memory. Nat Neurosci. 11:834–842. 10.1038/nn.2149. 18552840

[ref73] Van Strien NM , Cappaert NLM, Witter MP. 2009. The anatomy of memory: an interactive overview of the parahippocampal–hippocampal network. Nat Rev Neurosci. 10:272–282. 10.1038/nrn2614. 19300446

[ref74] Vann SD , Tsivilis D, Denby CE, Quamme JR, Yonelinas AP, Aggleton JP, Montaldi D, Mayes AR. 2009. Impaired recollection but spared familiarity in patients with extended hippocampal system damage revealed by 3 convergent methods. Proc Natl Acad Sci U S A. 106:5442–5447. 10.1073/pnas.0812097106. 19289844 PMC2664061

[ref75] Viskontas IV , Knowlton BJ, Fried I. 2016. Responses of neurons in the medial temporal lobe during encoding and recognition of face-scene pairs. Neuropsychologia. 90:200–209. 10.1016/j.neuropsychologia.2016.07.014. 27424273 PMC5510888

[ref76] Wais PE , Wixted JT, Hopkins RO, Squire LR. 2006. The hippocampus supports both the recollection and the familiarity components of recognition memory. Neuron. 49:459–466. 10.1016/J.NEURON.2005.12.020. 16446148 PMC1457095

[ref77] Warrington E . 1984. The recognition memory test. Windsor, UK: NFER-Nelson.

[ref78] Warrington EK . 1996. The camden memory tests. Hove (UK): Psychology Press.

[ref79] Warrington EK , James M. 1991. The visual object and space perception battery. Bury St. Edmunds (UK): Thames Valley Test Company.

[ref80] Wechsler D . 2011. WASI-II: Wechsler abbreviated scale of intelligence. 2nd ed. WASI. San Antonio, TX: Psychological Corporation.

[ref81] Wechsler D . 1997. Wechsler memory scale. 3rd ed. San Antonio, TX: Psychological Corporation.

[ref82] Wixted JT , Squire LR. 2011a. The medial temporal lobe and the attributes of memory. Trends Cogn Sci. 15:210–217. 10.1016/J.TICS.2011.03.005. 21481629 PMC3085543

[ref83] Wixted JT , Squire LR. 2011b. The familiarity/recollection distinction does not illuminate medial temporal lobe function: response to Montaldi and Mayes. Trends Cogn Sci. 15:340–341. 10.1016/j.tics.2011.06.006. 21763175 PMC3457644

[ref84] Yonelinas AP . 1994. Receiver-operating characteristics in recognition memory: evidence for a dual-process model. J Exp Psychol Learn Mem Cogn. 20:1341–1354. 10.1037/0278-7393.20.6.1341. 7983467

[ref85] Yonelinas AP , Dobbins I, Szymanski MD, Dhaliwal HS, King L. 1996. Signal-detection, threshold, and dual-process models of recognition memory: ROCs and conscious recollection. Conscious Cogn. 5:418–441. 10.1006/CCOG.1996.0026. 9063609

[ref86] Yonelinas AP , Kroll NEA, Quamme JR, Lazzara MM, Sauvé M-J, Widaman KF, Knight RT. 2002. Effects of extensive temporal lobe damage or mild hypoxia on recollection and familiarity. Nat Neurosci. 5:1236–1241. 10.1038/nn961. 12379865

[ref87] Yonelinas AP , Parks CM. 2007. Receiver operating characteristics (ROCs) in recognition memory: a review. Psychol Bull. 133:800–832. 10.1037/0033-2909.133.5.800. 17723031

[ref88] Yushkevich PA , Piven J, Hazlett HC, Smith RG, Ho S, Gee JC, Gerig G. 2006. User-guided 3D active contour segmentation of anatomical structures: significantly improved efficiency and reliability. Neuroimage. 31:1116–1128. 10.1016/j.neuroimage.2006.01.015. 16545965

[ref89] Zeidman P , Mullally SL, Maguire EA. 2015. Constructing, perceiving, and maintaining scenes: hippocampal activity and connectivity. Cereb Cortex. 25:3836–3855. 10.1093/cercor/bhu266. 25405941 PMC4585517

