# Supplementary material for: Functional Specialization of the Medial Temporal Lobes in Human Recognition Memory: Dissociating Effects of Hippocampal versus Parahippocampal Damage
Source: Cereb Cortex. 2021 Sep 17;32(8):1637–52. doi: 10.1093/cercor/bhab290 (PMC9016283; doi:10.1093/cercor/bhab290)
Supplement: argyropoulos_et_al_cercor_supplementary_material_03_07_2021_bhab290 [file argyropoulos_et_al_cercor_supplementary_material_03_07_2021_bhab290.docx]

# Supplementary Information

## Supplementary Section 1

### ROC – Stimulus Materials – Words

160 words (targets: n=80; foils: n = 80) were common, singular nouns. Those used for targets and foils were matched according to i) corpus frequency [SUBTLEXUS word frequency (Brysbaert and New, 2009)] (targets: M = 15.58, IQR = 28.32; foils: M = 15.14; IQR = 15.78 occurrences per million words; targets vs. foils: U = 3039.50, p = 0.585); ii) length (targets: M = 5; IQR = 2; foils: M = 5, IQR = 2; targets vs. foils: U = 3074.50, p = 0.658); iii) mean concreteness ratings (targets: M = 5.81, IQR = 1.54; foils: M = 5.80; IQR = 1.87; targets vs. foils: U = 1684.00, p = 0.631); iv) mean imageability ratings (targets: M = 5.91; IQR = 1.39; foils: M = 5.97, IQR = 1.74; targets vs. foils: U = 1688.50, p = 0.648); v) mean familiarity ratings (targets: M = 5.65, IQR = 0.86; foils: M = 5.71, IQR = 0.77; targets vs. foils: U = 1701.50, p = 0.698); vi) age of acquisition (targets: mean = 3.43; SD = 1.01; foils: mean = 3.34 ; SD = 0.91; targets vs. foils: t = 0.49, p =0.63); vii) mean ratings of arousal levels (targets: mean = 4.49; SD = 0.94; foils: mean = 4.29; SD = 0.98; targets vs. foils: t = 1.12, p = 0.26); viii) mean valence ratings (targets: Μ = 5.29; IQR = 0.77; foils: M = 5.20; IQR = 1.06; targets vs. foils: U = 1653.50, p = 0.521) [see (Scott et al., 2018) for details on ratings of concreteness, imageability, familiarity, age of acquisition, arousal, and valence]. They were presented in the center of the display (font size: 28).

In the study phase, word stimuli were presented for 3 seconds (faces and scenes: 4.5 seconds), in order to control for task difficulty. Indeed, the parameter estimates (from applying the independent dual-process model to ROCs) for CTRs did not differ across the three material-types for either recollection (one-way repeated-measures ANOVA; independent variable: Material-Type(3); F<0.5, p=0.728; pair-wise t-tests: all ts, |t| ≤ 0.759; all ps, p ≥ 0.464) or familiarity (F<1, p=0.474; pair-wise t-tests: all ts, |t| ≤ 1.23; all ps, p ≥ 0.244), suggesting that the three material-types did not differ with respect to difficulty. Based upon extensive piloting, we equated levels of difficulty across material-types, using two studies and, correspondingly, two test phases for scenes and faces, but one study and one test phase for words. Moreover, the study phase for words was positioned at the beginning of the session, and the recognition phase for words at the end, similar to other studies [e.g. (Cipolotti et al., 2006)].

### RDP – Stimulus Materials – Words

Due to an error in the design of the word stimuli for this task, the 60 words used (n = 30 targets and n = 30 foils) were the same in the first (long response deadline) and the second (short response deadline) sessions (unlike face and scene stimuli).

Targets and foils did not differ in corpus frequency [SUBTLEXUS word frequency (Brysbaert and New, 2009)] (targets: Μ = 17.36; IQR = 31.52 occurrences per million words; foils: M = 17.39; IQR= 26.43 occurrences per million words; targets vs. foils: U = 437, p = 0.854); iv) length (targets: M= 5; IQR = 2; foils: M = 5; IQR = 2; targets vs. foils: U = 427, p = 0.741); v) mean concreteness ratings (targets: Μ = 6.00, IQR = 1.98; foils: M = 5.68; IQR = 1.76; targets vs. foils: U = 252.5, p = 0.995); vi) mean imageability ratings (targets: Μ = 6.06, IQR = 1.34; foils: M = 6.13, IQR = 1.29; targets vs. foils: U =241, p =0.792); vii) mean familiarity ratings (targets: M = 5.94, IQR = 0.76; foils: M = 5.94, IQR = 0.73; targets vs. foils: U = 239.5, p = 0.766); viii) age of acquisition (targets: M = 2.87; IQR = 1.48; foils: M = 2.60; IQR = 1.43; targets vs. foils: U = 220.5, p = 0.468) ; ix) mean ratings of arousal levels (targets: mean = 4.54, SD =1.05; foils: mean = 4.31; SD = 0.94; targets vs. foils: t = 0.77, p = 0.443); x) mean valence ratings (targets: M = 5.47, IQR = 1.04; foils: M = 5.49; IQR = 0.81; targets vs. foils: U = 242.0, p = 0.809).

In the study phase, participants were given 3 seconds to rate pleasantness for words.

## Supplementary figure 1


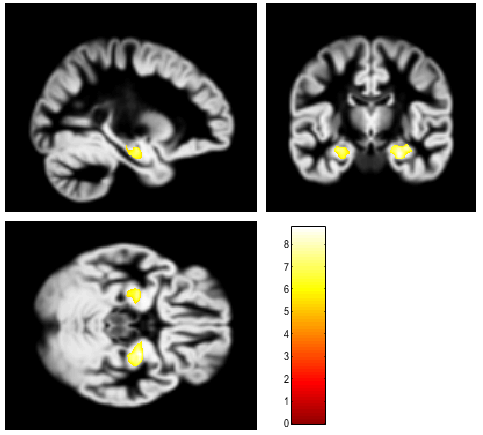


VBM results for contrast ‘CTRs (n=67) > HPC patients (n=8)’ on modulated GM tissue maps (reflecting GM volume). Volume reduction is noted bilaterally in the anterior HPC: Cluster 1: Right anterior HPC: cluster size: kE = 1865 voxels; peak coordinates: x=25, y=-11, z=-19 mm; t = 8.74; p-FWE < 0.0005; Cluster 2: Left anterior HPC: cluster size: kE = 863 voxels; peak coordinates: x=-25; y=-13; z=-19; t = 7.29 mm; p-FWE < 0.0005. Rest of ps: p-FWE > 0.15. Second-level between-subjects covariates: age at research scan; sex; study [see (Argyropoulos et al., 2019) for details]; total intracranial volume]. The voxels presented survive peak-level whole-brain FWE-correction (p < 0.05). Clusters are overlaid on an ICBM152 GM template (MNI space). Color-bar indicates t-values.

## Supplementary figure 2


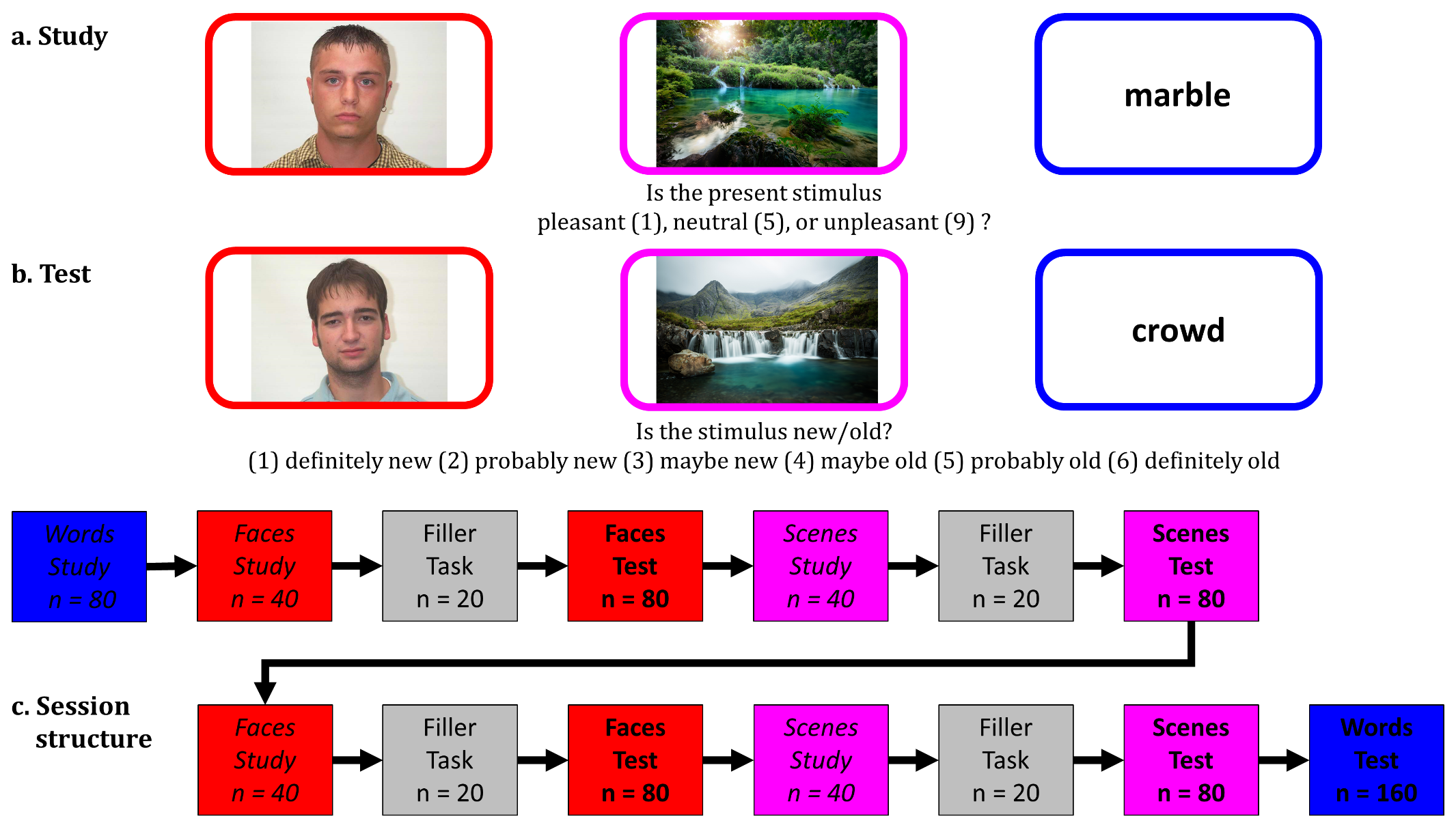


Paradigm 1 (ROC): Paradigm involving analysis of Receiver Operating Characteristics; a. Study phase: participants judged whether each stimulus was ‘pleasant’, ‘neutral’, or ‘unpleasant’; b. Test phase: participants were presented with each stimulus and were asked to judge, in a self-paced fashion, whether they have each stimulus before, rating their confidence on a scale from 1 to 6; c: session structure: the order of blocks was held constant across participants; the order of trials within each block was randomized for each session. Faces and Scenes were tested in two study-test blocks (separated by a filler task), whereas words were tested in a single pair of blocks at start and end of experiment, because memory for words is generally superior overall. blue: words; red: faces; green: scenes; grey: filler task; n: number of trials per block.

## Supplementary figure 3


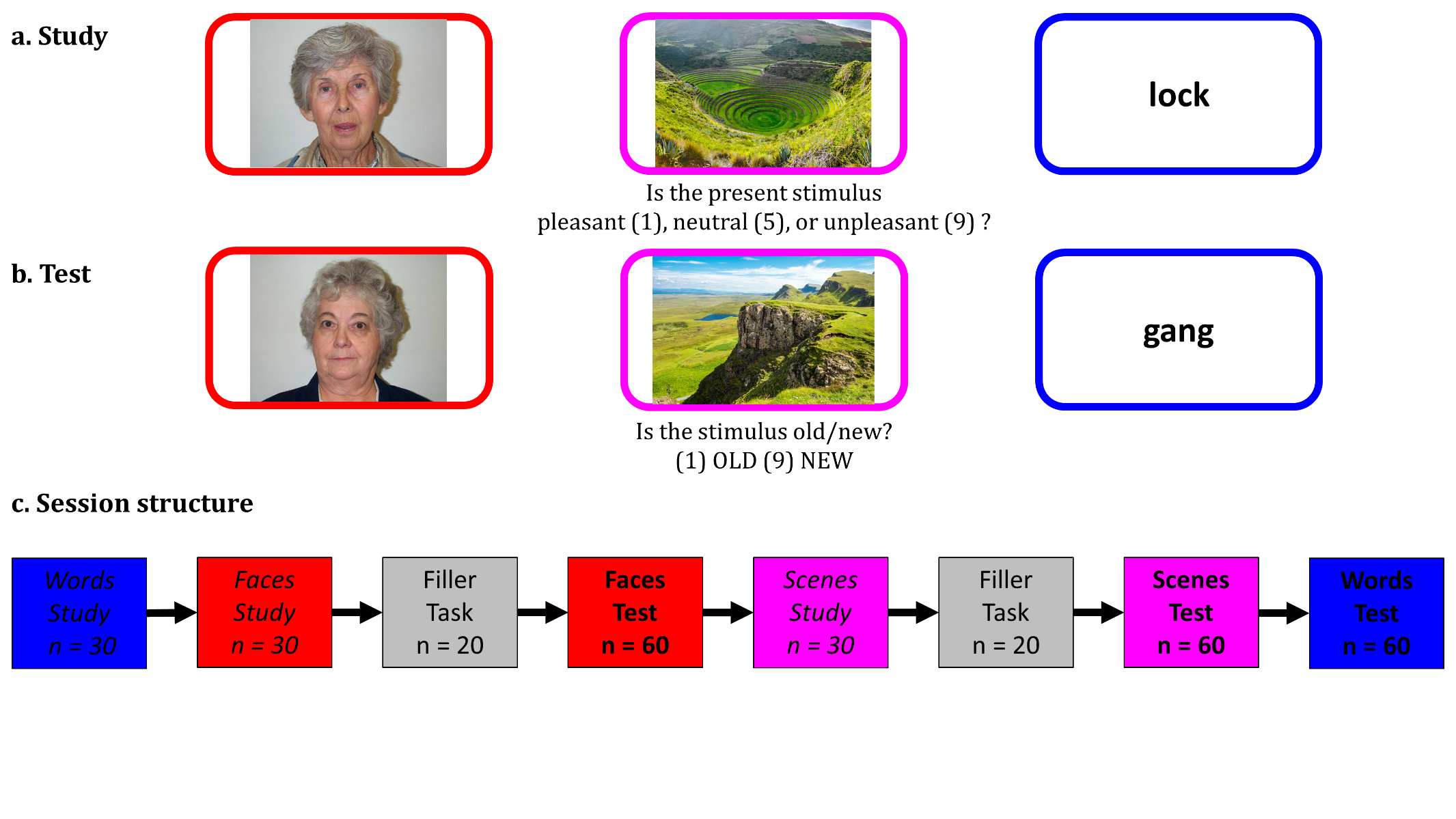


Paradigm 2 (RDP): Response Deadline Paradigm; a. Study phase: participants judged whether each stimulus was ‘pleasant’, ‘neutral’, or ‘unpleasant’; b. Test phase: participants were presented with each stimulus and were asked to judge whether they had encountered each stimulus before; c: session structure: the order of blocks was held constant across participants; the order of trials within each block was randomized for each session – see Supplementary Figure 2 legend for more details. The short versus long response deadline was manipulated across two sessions on different days (see Methods). blue: words; red: faces; green: scenes; grey: filler task; *n:* number of trials per block.

## Supplementary figure 4


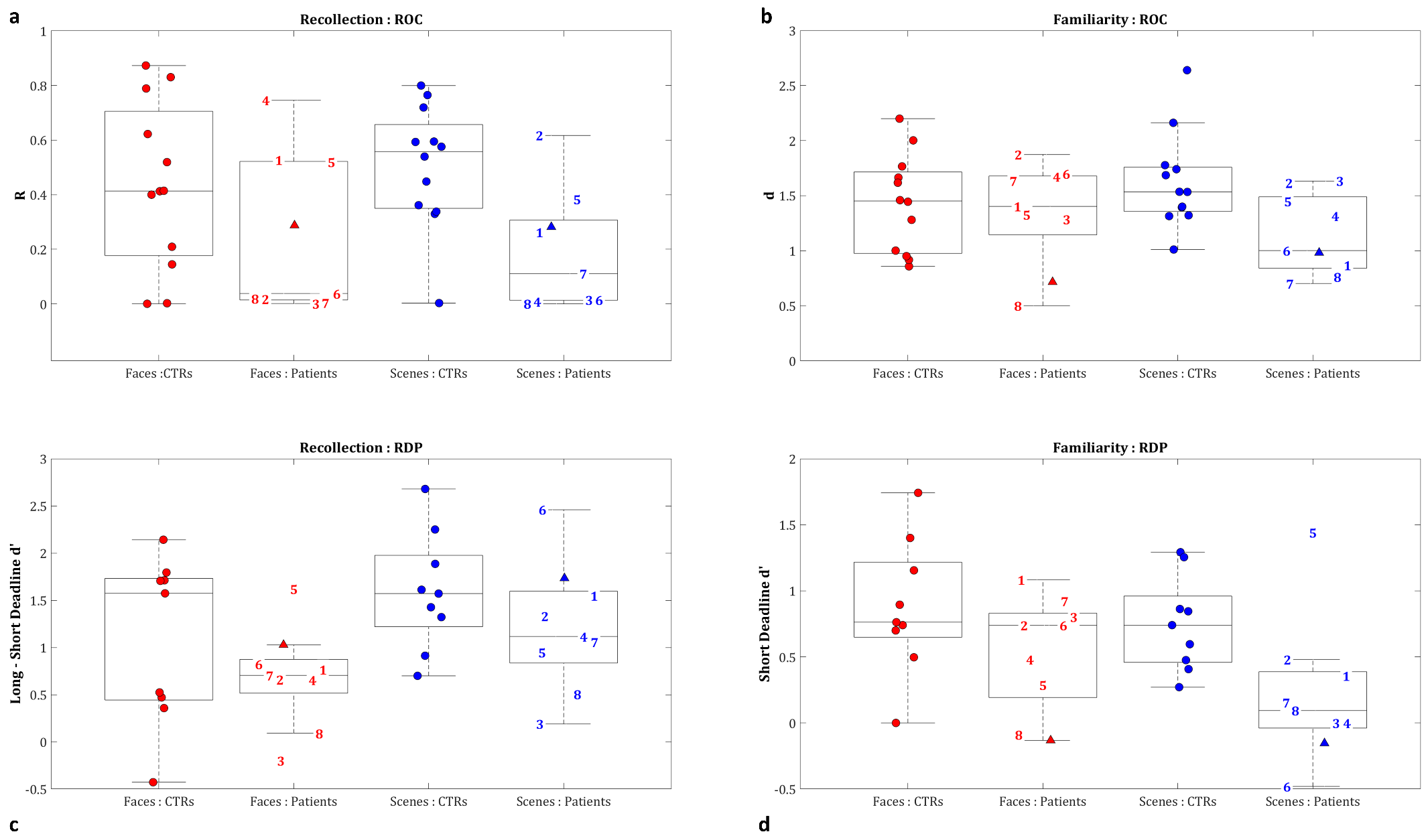


Recollection and familiarity estimates for CTRs and patients for face and scene recognition memory in Paradigms 1 and 2; **a,c: recollection estimates; b,d: familiarity estimates;** in Paradigm 2 (RDP), familiarity is reflected by the sensitivity indices (d’) for the short deadline, whereas recollection by the difference between the d’ for the long response deadline and that for the short response deadline. Line in boxplots=median; bottom of box=25^th^ %ile; top=75^th^ %ile; whiskers: 1.5 * interquartile range; **key:** red: performance on faces; blue: performance on scenes; **L, R**: left/right hemisphere; ● **CTRs** : healthy controls; ▲: **MH** (patient with right PRC lesion); **H1-7**: patients with HPC but no PRC lesion; H8: patient with both HPC and right PRC lesions; **PRC:** perirhinal cortex; **HPC:** hippocampus;

## Supplementary figure 5


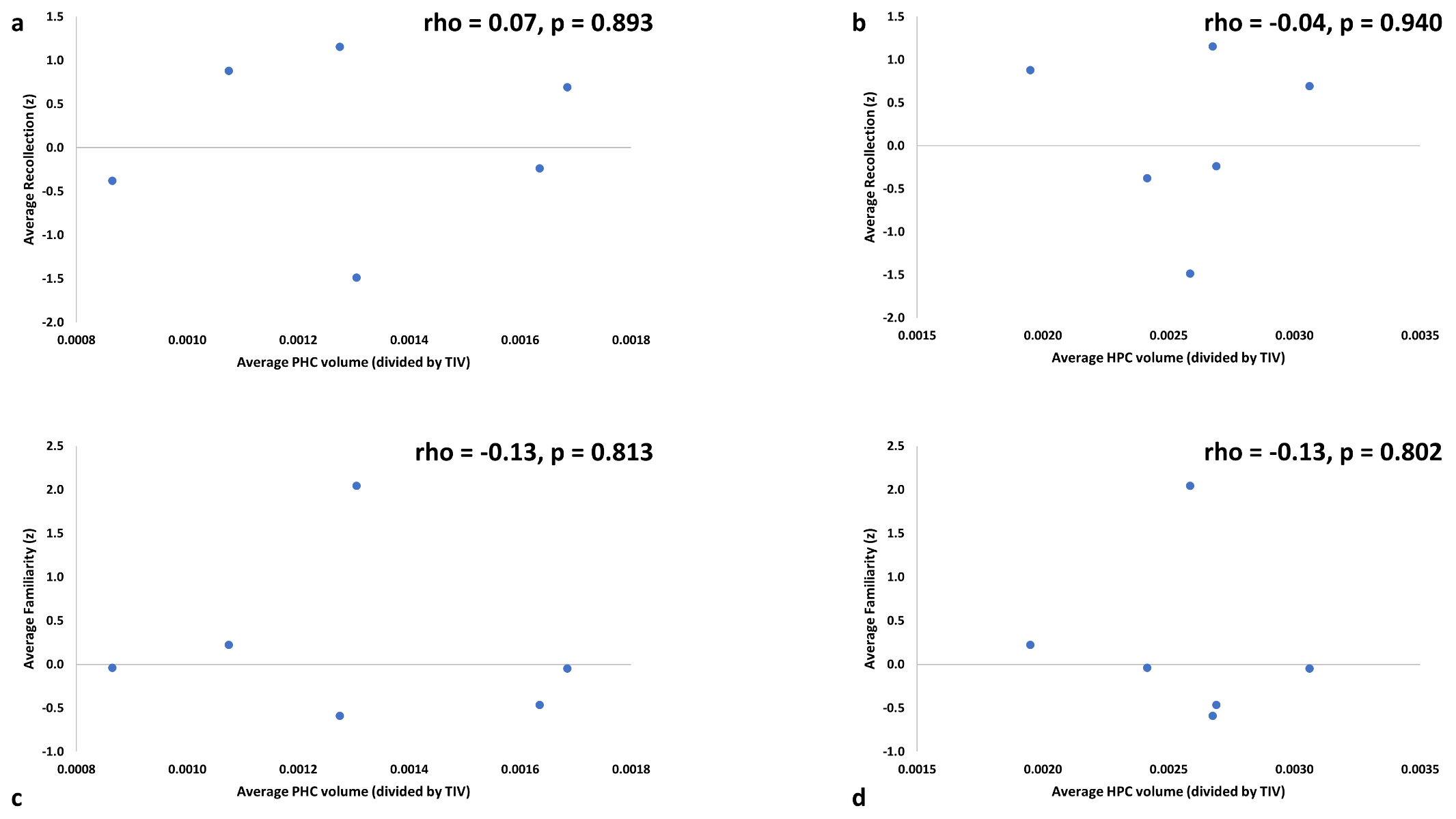


Relationship of HPC (b, d) and PHC (a, c) volumes with recollection (a, b) and familiarity estimates (c, d) across CTRs (Material-Type: Faces vs. Scenes); volume is divided by TIV; 6 CTRs had volumetric data available; recollection and familiarity estimates are separately z-transformed (on the basis of the mean and SD of all CTRs) and averaged across conditions (here and elsewhere, excluding performance on Words). No trend for a positive relationship was noted between familiarity and PHC volume or between recollection and HPC volume across CTRs; **key**: **CTR**s: healthy controls; **HPC**: hippocampus; **PHC:** parahippocampal cortex; **rho:** Spearman’s rank correlation coefficient; **TIV:** total intracranial volume.

## Supplementary table 1: Neuropsychological Profiles

| Test | NART | WASI-II | | | GNT * | C&CT * | VOSP | | | DKEFS | WMS-III | | | | | | RMT | | | ROCFT | | D&P | | | |
| --- | --- | --- | --- | --- | --- | --- | --- | --- | --- | --- | --- | --- | --- | --- | --- | --- | --- | --- | --- | --- | --- | --- | --- | --- | --- |
|  | p-FSIQ | MR | SI | VC |  |  | DC | CA | PD | LNS | DS | LM1 | LM2 | WL1 | WL2 | WL rec | Scenes | Faces | Words | Imm. | Del. | Names | People | Shapes | Doors |
| Function | Intelligence, Semantic Memory and Language | | | | | | Visuospatial | | | Executive | | Episodic Memory | | | | | | | | | | | | | |
| Patient |  | | | | | | | | | | | | | | | | | | | | | | | | |
| MH - PRC lesion | 0.80 | -0.75 | 0.67 | 0.88 | 0.39 | 0.02 | > 5^th^ %ile | | | -0.33 | 0.00 | n/a | | -0.67 | -0.33 | 1.00 | -1.12 | -2.33 | 0.00 | -0.75 | -0.82 | 2.67 | -0.33 | 0.33 | 0.67 |
| H1 -HPC lesion | 0.00 | -0.30 | -0.60 | 0.00 | -0.67 | 0.02 |  |  |  | -1.00 | -1.33 | -0.67 | 0.00 | -0.33 | 1.33 | 1.00 | -0.49 | -1.00 | 1.00 | -0.42 | 0.00 | 1.00 | -2.00 | 1.33 | 0.33 |
| H2 - HPC lesion | 1.20 | 0.30 | 1.00 | 0.60 | -0.10 | 0.99 |  |  |  | 0.00 | 0.33 | 1.00 | -0.67 | -0.67 | 0.33 | 1.00 | 1.68 | -0.67 | 0.67 | 0.40 | 0.00 | -1.67 | 0.67 | 1.33 | 2.33 |
| H3 - HPC lesion | 1.07 | 1.20 | 1.00 | 2.00 | -0.10 | 0.34 |  |  |  | -0.33 | 0.33 | -1.33 | -0.33 | 1.33 | -0.33 | -0.67 | 1.13 | 0.00 | -0.33 | 0.79 | 1.26 | -1.33 | -2.00 | -0.67 | 0.33 |
| H4 - HPC lesion | -0.13 | -1.20 | 0.70 | -0.80 | -0.83 | 0.67 |  |  |  | 1.33 | 0.33 | -1.00 | -2.33 | -2.00 | 0.00 | -0.33 | 0.09 | -0.33 | 0.00 | -3.00 | -3.00 | -2.33 | -0.33 | -1.00 | -1.00 |
| H5 - HPC lesion | 1.87 | 1.00 | 0.70 | 2.10 | 1.12 | 0.67 |  |  |  | 1.67 | 2.67 | 0.33 | -1.00 | -0.33 | 0.67 | -1.33 | n/a | 1.67 | 1.00 | 1.70 | 1.44 | 2.00 | -0.67 | 0.67 | 0.67 |
| H6 - HPC lesion | 1.53 | 0.10 | 1.80 | 3.00 | 0.63 | -0.64 |  |  |  | 0.33 | -0.33 | -2.00 | -2.67 | 0.00 | -1.33 | -1.33 | -1.84 | 2.33 | -1.33 | -2.16 | -3.00 | 0.67 | -1.67 | -2.00 | -1.67 |
| H7 - HPC lesion | 1.47 | -0.60 | 0.60 | 0.10 | -0.34 | 0.34 |  |  |  | 0.00 | -0.33 | -0.33 | -2.00 | 0.00 | 1.33 | 1.33 | -1.84 | 0.00 | 0.67 | -1.20 | -1.31 | -2.67 | -2.00 | -2.33 | -0.67 |
| H8 - HPC and PRC lesion | 1.13 | 1.70 | 1.50 | -1.00 | 0.88 | 0.99 |  |  |  | 1.33 | 2.00 | -1.67 | -1.67 | -2.00 | -1.33 | -2.00 | -0.83 | -2.33 | -1.33 | -3.00 | -1.51 | 0.00 | -1.67 | -1.33 | -0.67 |

Patients’ neuropsychological profile. Scores are age-scaled standardized scores (z), except for those marked with * (not age-scaled: based on the published means and standard deviations); **key:** **C&CT:** Camel and Cactus Test (Bozeat et al., 2000); **DC:** dot counting; **DKEFS:** Delis-Kaplan executive function system(Delis et al., 2001); **D&P:** Doors and People test (Baddeley et al., 1994); **DS:** digit span (WMS-III); **GNT:** Graded Naming Test (Mckenna and Warrington, 1980); **LNS:** letter-number switching (DKEFS); **MR:** Matrix Reasoning (WASI-II); **NART:** national adult reading test (Nelson and Willison, 1991); **PD:** position discrimination (VOSP); **p-FSIQ:** premorbid full-scale intelligence quotient (NART); **RMT:** Recognition Memory Test and Camden Memory Test for scenes (Warrington, 1984, 1996); **ROCFT:** Rey-Osterrieth Complex Figures Test (Rey, 1959); **SI:** Similarities (WASI-II); **VOSP:** Visual Object and Space Perception battery(Warrington and James, 1991); **WASI-II:** Wechsler Abbreviated Scale of Intelligence 2^nd^ Edition (Wechsler, 2011); **WL1:** word-list immediate recall (WMS-III); **WL2:** word-list delayed recall (WMS-III); **WL rec:** Word-list recognition test; **VC:** Vocabulary (WASI-II); **WMS-III:** Wechsler Memory Scale 3d edition (Wechsler, 1997); highlighted: z ≤ -1.67, i.e. the conventional cut-off point that corresponds to the 5^th^ %ile commonly employed to define impairment in neuropsychological assessment; **n/a:** not available (tests not administered due to scheduling conflicts).

## Supplementary table 2: Categorical Analyses: MH vs. CTRs

| **Analysis** | **Main Effect / Interaction** | **df1** | **df2** | **MICE IMPUTATION** | | | | | | | | | | | | | | |
| --- | --- | --- | --- | --- | --- | --- | --- | --- | --- | --- | --- | --- | --- | --- | --- | --- | --- | --- |
|  |  |  |  | **1** | | | **2** | | | **3** | | | **4** | | | **5** | | |
|  |  |  |  | **F** | **p** | **p-perm** | **F** | **p** | **p-perm** | **F** | **p** | **p-perm** | **F** | **p** | **p-perm** | **F** | **p** | **p-perm** |
| **MH vs CTRs**  **(4-way ANOVA:**  **Group,**  **Process,**  **Material-Type,**  **Paradigm)** | **Group** | 1 | 13 | 7.46 | 0.017 | 0.018 | 5.87 | 0.031 | 0.030 | 5.12 | 0.041 | 0.041 | 7.35 | 0.018 | 0.021 | 5.81 | 0.031 | 0.032 |
|  | **Process** | 1 | 13 | 0.17 | 0.686 | 0.684 | 0.24 | 0.629 | 0.623 | 0.27 | 0.612 | 0.610 | 0.23 | 0.636 | 0.640 | 0.18 | 0.680 | 0.682 |
|  | **Group*Process** | 1 | 13 | 3.87 | 0.071 | 0.071 | 2.56 | 0.133 | 0.135 | 3.54 | 0.083 | 0.086 | 3.34 | 0.091 | 0.097 | 3.67 | 0.078 | 0.079 |
|  | **Material-Type** | 1 | 13 | 3.12 | 0.101 | 0.107 | 1.60 | 0.229 | 0.221 | 1.56 | 0.233 | 0.231 | 4.43 | 0.055 | 0.062 | 4.26 | 0.060 | 0.056 |
|  | **Group*Material-Type** | 1 | 13 | 0.30 | 0.594 | 0.590 | 0.06 | 0.815 | 0.808 | 0.17 | 0.690 | 0.688 | 0.46 | 0.510 | 0.513 | 0.31 | 0.586 | 0.569 |
|  | **Paradigm** | 1 | 13 | 0.55 | 0.473 | 0.452 | 0.61 | 0.449 | 0.450 | 0.64 | 0.440 | 0.425 | 0.12 | 0.733 | 0.724 | 0.52 | 0.485 | 0.484 |
|  | **Group*Paradigm** | 1 | 13 | 0.13 | 0.725 | 0.723 | 0.16 | 0.697 | 0.700 | 0.13 | 0.722 | 0.709 | < 0.05 | 0.946 | 0.949 | 0.11 | 0.744 | 0.746 |
|  | **Material-Type*Process** | 1 | 13 | 0.19 | 0.667 | 0.667 | 0.35 | 0.566 | 0.567 | 0.31 | 0.587 | 0.576 | 0.19 | 0.673 | 0.682 | 0.28 | 0.604 | 0.594 |
|  | **Group*Material-Type*Process** | 1 | 13 | < 0.05 | 0.956 | 0.957 | < 0.05 | 0.989 | 0.990 | < 0.05 | 0.988 | 0.987 | < 0.05 | 0.840 | 0.842 | < 0.05 | 0.996 | 0.997 |
|  | **Paradigm*Process** | 1 | 13 | 13.49 | 0.003 | 0.003 | 22.52 | < 0.001 | < 0.001 | 16.42 | 0.001 | 0.002 | 16.73 | 0.001 | 0.002 | 17.50 | 0.001 | 0.001 |
|  | **Group*Paradigm*Process** | 1 | 13 | 0.42 | 0.527 | 0.525 | 0.30 | 0.594 | 0.570 | 0.40 | 0.537 | 0.526 | 0.52 | 0.482 | 0.480 | 0.54 | 0.476 | 0.476 |
|  | **Paradigm*Material-Type** | 1 | 13 | 0.18 | 0.682 | 0.669 | 0.11 | 0.748 | 0.735 | 0.16 | 0.699 | 0.712 | 0.16 | 0.698 | 0.696 | 0.19 | 0.671 | 0.667 |
|  | **Group*Paradigm*Material-Type** | 1 | 13 | 0.27 | 0.615 | 0.617 | 0.07 | 0.798 | 0.788 | 0.08 | 0.782 | 0.793 | 0.17 | 0.689 | 0.681 | 0.23 | 0.640 | 0.641 |
|  | **Paradigm*Material-Type*Process** | 1 | 13 | 1.41 | 0.256 | 0.252 | 1.85 | 0.198 | 0.192 | 1.84 | 0.198 | 0.197 | 1.34 | 0.269 | 0.265 | 1.35 | 0.266 | 0.261 |
|  | **Group*Paradigm*Material-Type*Process** | 1 | 13 | 0.09 | 0.769 | 0.770 | 0.05 | 0.825 | 0.824 | 0.05 | 0.835 | 0.839 | 0.08 | 0.781 | 0.777 | < 0.05 | 0.880 | 0.884 |
|  | | | | | | | | | | | | | | | | | | |
| **MH vs CTRs**  **Recollection**  **(3-way ANOVA:**  **Group,**  **Material-Type,**  **Paradigm)** | **Group** | 1 | 13 | 0.06 | 0.809 | 0.802 | 0.09 | 0.775 | 0.763 | 0.07 | 0.801 | 0.789 | 0.05 | 0.819 | 0.811 | 0.08 | 0.785 | 0.787 |
|  | **Material-Type** | 1 | 13 | 0.97 | 0.342 | 0.347 | 0.98 | 0.340 | 0.322 | 1.08 | 0.318 | 0.295 | 1.37 | 0.263 | 0.259 | 1.25 | 0.284 | 0.293 |
|  | **Group*Material-Type** | 1 | 13 | 0.06 | 0.813 | 0.810 | < 0.05 | 0.915 | 0.908 | 0.05 | 0.830 | 0.824 | 0.19 | 0.673 | 0.670 | < 0.05 | 0.858 | 0.855 |
|  | **Paradigm** | 1 | 13 | 11.97 | 0.004 | 0.004 | 17.11 | 0.001 | 0.001 | 13.30 | 0.003 | 0.004 | 9.18 | 0.010 | 0.009 | 12.51 | 0.004 | 0.004 |
|  | **Group*Paradigm** | 1 | 13 | 0.14 | 0.714 | 0.714 | < 0.05 | 0.836 | 0.825 | 0.13 | 0.729 | 0.721 | 0.21 | 0.655 | 0.651 | 0.16 | 0.699 | 0.690 |
|  | **Paradigm*Material-Type** | 1 | 13 | 1.40 | 0.257 | 0.245 | 1.26 | 0.282 | 0.269 | 1.64 | 0.223 | 0.215 | 1.30 | 0.274 | 0.269 | 1.48 | 0.246 | 0.240 |
|  | **Group*Paradigm*Material-Type** | 1 | 13 | 0.28 | 0.608 | 0.591 | 0.12 | 0.738 | 0.718 | 0.13 | 0.723 | 0.712 | 0.22 | 0.647 | 0.629 | 0.15 | 0.707 | 0.692 |
|  | | | | | | | | | | | | | | | | | | |
| **MH vs CTRs**  **Familiarity**  **(3-way ANOVA:**  **Group,**  **Material-Type,**  **Paradigm)** | **Group** | 1 | 13 | 18.78 | 0.001 | 0.001 | 15.35 | 0.002 | 0.001 | 14.98 | 0.002 | 0.002 | 15.25 | 0.002 | 0.002 | 12.79 | 0.003 | 0.002 |
|  | **Material-Type** | 1 | 13 | 0.14 | 0.717 | 0.712 | 0.25 | 0.627 | 0.616 | 0.11 | 0.751 | 0.738 | 0.34 | 0.573 | 0.579 | 0.13 | 0.722 | 0.719 |
|  | **Group*Material-Type** | 1 | 13 | < 0.05 | 0.843 | 0.847 | 0.06 | 0.810 | 0.796 | 0.07 | 0.794 | 0.785 | < 0.05 | 0.909 | 0.908 | 0.07 | 0.796 | 0.795 |
|  | **Paradigm** | 1 | 13 | 8.40 | 0.012 | 0.008 | 13.25 | 0.003 | 0.002 | 12.13 | 0.004 | 0.005 | 10.30 | 0.007 | 0.007 | 12.53 | 0.004 | 0.004 |
|  | **Group*Paradigm** | 1 | 13 | 0.58 | 0.458 | 0.455 | 0.51 | 0.488 | 0.484 | 0.64 | 0.437 | 0.449 | 0.44 | 0.519 | 0.522 | 0.82 | 0.383 | 0.385 |
|  | **Paradigm*Material-Type** | 1 | 13 | 0.58 | 0.461 | 0.475 | 0.41 | 0.533 | 0.534 | 0.43 | 0.524 | 0.518 | 0.46 | 0.509 | 0.498 | 0.57 | 0.464 | 0.459 |
|  | **Group*Paradigm*Material-Type** | 1 | 13 | < 0.05 | 0.962 | 0.966 | < 0.05 | 0.959 | 0.962 | < 0.05 | 0.962 | 0.966 | < 0.05 | 0.979 | 0.977 | < 0.05 | 0.904 | 0.910 |

ANOVAs (between-participants independent variables: Group (MH (PRC lesion) vs. CTRs); within-participants independent variables: Paradigm (ROC vs. RDP); Process (Familiarity vs. Recollection); Material-Type (Faces vs. Scenes) on (recollection and familiarity) estimates for MH (focal PRC lesion) and CTRs for faces and scenes in the ROC and the RDP paradigms. Missing values were imputed using “Multiple Imputation with Chained Equations” implemented in the R function “mice”. Five imputations were created (MICE 1-5). shaded cells: significant (p < 0.05) main effects and interactions of interest. ‘p-perm’: p-values calculated from 5000 permutations using the “aovperm” function in R.

## Supplementary table 3: Categorical Analyses: HPC Patients vs. CTRs

| **Analysis** | **Main Effect / Interaction** | **df1** | **df2** | **MICE IMPUTATION** | | | | | | | | | | | | | | | | | | | |
| --- | --- | --- | --- | --- | --- | --- | --- | --- | --- | --- | --- | --- | --- | --- | --- | --- | --- | --- | --- | --- | --- | --- | --- |
|  |  |  |  | **1** | | | **2** | | | | | **3** | | | | | | **4** | | | **5** | | |
|  |  |  |  | **F** | **p** | **p-perm** | **F** | **p** | | **p-perm** | | **F** | | **p** | | **p-perm** | | **F** | **p** | **p-perm** | **F** | **p** | **p-perm** |
| **HPC (H1-H7) vs. CTRs**  **(4-way ANOVA)** | **Group** | 1 | 19 | 13.66 | 0.002 | 0.002 | 11.53 | 0.003 | | 0.003 | | 10.06 | | 0.005 | | 0.005 | | 12.26 | 0.002 | 0.003 | 11.86 | 0.003 | 0.004 |
|  | **Process** | 1 | 19 | 14.60 | 0.001 | 0.001 | 9.87 | 0.005 | | 0.006 | | 12.98 | | 0.002 | | 0.002 | | 12.52 | 0.002 | 0.003 | 13.91 | 0.001 | 0.001 |
|  | **Group*Process** | 1 | 19 | < 0.05 | 0.874 | 0.877 | 0.21 | 0.649 | | 0.649 | | 0.16 | | 0.690 | | 0.693 | | 0.13 | 0.727 | 0.728 | < 0.05 | 0.846 | 0.842 |
|  | **Material-Type** | 1 | 19 | 0.23 | 0.634 | 0.630 | 0.40 | 0.533 | | 0.538 | | 0.11 | | 0.739 | | 0.741 | | 0.26 | 0.619 | 0.622 | 0.43 | 0.518 | 0.505 |
|  | **Group*Material-Type** | 1 | 19 | 5.11 | 0.036 | 0.032 | 3.64 | 0.072 | | 0.071 | | 2.95 | | 0.102 | | 0.100 | | 6.30 | 0.021 | 0.022 | 6.71 | 0.018 | 0.018 |
|  | **Paradigm** | 1 | 19 | 0.37 | 0.549 | 0.559 | 0.47 | 0.501 | | 0.499 | | 0.32 | | 0.579 | | 0.569 | | < 0.05 | 0.977 | 0.973 | 0.30 | 0.590 | 0.598 |
|  | **Group*Paradigm** | 1 | 19 | 3.07 | 0.096 | 0.097 | 3.37 | 0.082 | | 0.082 | | 3.24 | | 0.088 | | 0.080 | | 0.91 | 0.352 | 0.355 | 2.89 | 0.106 | 0.104 |
|  | **Material-Type*Process** | 1 | 19 | 3.79 | 0.067 | 0.068 | 5.34 | 0.032 | | 0.030 | | 5.02 | | 0.037 | | 0.040 | | 4.01 | 0.060 | 0.057 | 4.69 | 0.043 | 0.044 |
|  | **Group*Material-Type*Process** | 1 | 19 | 1.18 | 0.291 | 0.295 | 1.06 | 0.317 | | 0.318 | | 1.18 | | 0.290 | | 0.292 | | 2.39 | 0.139 | 0.136 | 0.98 | 0.335 | 0.341 |
|  | **Paradigm*Process** | 1 | 19 | 63.15 | < 0.001 | < 0.001 | 99.15 | < 0.001 | | < 0.001 | | 75.16 | | < 0.001 | | < 0.001 | | 75.44 | < 0.001 | < 0.001 | 78.29 | < 0.001 | < 0.001 |
|  | **Group*Paradigm*Process** | 1 | 19 | 0.21 | 0.655 | 0.654 | < 0.05 | 0.952 | | 0.949 | | 0.10 | | 0.758 | | 0.758 | | 0.24 | 0.627 | 0.626 | 0.24 | 0.629 | 0.634 |
|  | **Paradigm*Material-Type** | 1 | 19 | 1.23 | 0.281 | 0.291 | 0.83 | 0.374 | | 0.376 | | 1.16 | | 2.942 | | 0.301 | | 1.15 | 0.298 | 0.301 | 1.33 | 0.264 | 0.266 |
|  | **Group*Paradigm*Material-Type** | 1 | 19 | 1.80 | 0.195 | 0.192 | 0.55 | 0.468 | | 0.475 | | 0.63 | | 0.437 | | 0.442 | | 1.22 | 0.283 | 0.282 | 1.58 | 0.223 | 0.213 |
|  | **Paradigm*Material-Type*Process** | 1 | 19 | 4.75 | 0.042 | 0.038 | 6.15 | 0.023 | | 0.023 | | 6.18 | | 0.022 | | 0.024 | | 4.59 | 0.045 | 0.044 | 5.07 | 0.036 | 0.038 |
|  | **Group*Paradigm*Material-Type*Process** | 1 | 19 | 0.03 | 0.864 | 0.859 | < 0.05 | 0.980 | | 0.981 | | 0.00 | | 0.963 | | 0.961 | | < 0.05 | 0.878 | 0.875 | < 0.05 | 0.923 | 0.919 |
|  | | | | | | | | | | | | | | | | | | | | | | | |
| **HPC (H1-H7) vs. CTRs:**  **Recollection**  **(3-way ANOVA)** | **Group** | 1 | 19 | 4.69 | 0.043 | 0.040 | 4.55 | 0.046 | | 0.047 | | 4.51 | | 0.047 | | 0.047 | | 4.66 | 0.044 | 0.046 | 4.93 | 0.039 | 0.037 |
|  | **Material-Type** | 1 | 19 | 3.48 | 0.078 | 0.073 | 3.88 | 0.064 | | 0.061 | | 3.87 | | 0.064 | | 0.067 | | 4.02 | 0.059 | 0.059 | 4.50 | 0.047 | 0.044 |
|  | **Group*Material-Type** | 1 | 19 | < 0.05 | 0.939 | 0.942 | < 0.05 | 0.852 | | 0.850 | | 1.95 | | 0.989 | | 0.990 | | 0.13 | 0.725 | 0.728 | < 0.05 | 0.930 | 0.933 |
|  | **Paradigm** | 1 | 19 | 43.67 | < 0.001 | < 0.001 | 59.38 | < 0.001 | | < 0.001 | | 4.76 | | < 0.001 | | < 0.001 | | 34.49 | < 0.001 | < 0.001 | 44.97 | < 0.001 | < 0.001 |
|  | **Group*Paradigm** | 1 | 19 | 0.31 | 0.583 | 0.576 | 1.19 | 0.289 | | 0.297 | | 4.37 | | 0.517 | | 0.512 | | 0.06 | 8.113 | 0.816 | 0.30 | 0.592 | 0.587 |
|  | **Paradigm*Material-Type** | 1 | 19 | 5.50 | 0.030 | 0.029 | 5.50 | 0.030 | | 0.027 | | 6.79 | | 0.017 | | 0.018 | | 5.32 | 0.033 | 0.029 | 6.17 | 0.022 | 0.022 |
|  | **Group*Paradigm*Material-Type** | 1 | 19 | 0.71 | 0.409 | 0.411 | 0.25 | 0.626 | | 0.633 | | 0.24 | | 6.278 | | 0.633 | | 0.56 | 0.463 | 0.462 | 0.31 | 0.584 | 0.581 |
|  | | | | | | | | | | | | | | | | | | | | | | | |
| **HPC (H1-H7) vs. CTRs:**  **Familiarity**  **(3-way ANOVA)** | **Group** | 1 | 19 | 10.80 | 0.004 | 0.003 | 7.71 | 0.012 | | 0.012 | | 7.53 | | 0.013 | | 0.012 | | 7.23 | 0.015 | 0.017 | 8.14 | 0.010 | 0.010 |
|  | **Material-Type** | 1 | 19 | 2.86 | 0.107 | 0.105 | 3.60 | 0.073 | | 0.080 | | 3.38 | | 0.082 | | 0.083 | | 2.67 | 0.119 | 0.117 | 3.46 | 0.078 | 0.076 |
|  | **Group*Material-Type** | 1 | 19 | 4.13 | 0.056 | 0.059 | 5.49 | 0.030 | | 0.032 | | 3.82 | | 0.066 | | 0.060 | | 6.16 | 0.023 | 0.019 | 4.21 | 0.054 | 0.054 |
|  | **Paradigm** | 1 | 19 | 44.40 | < 0.001 | < 0.001 | 65.79 | < 0.001 | | < 0.001 | | 60.42 | | < 0.001 | | < 0.001 | | 53.68 | < 0.001 | < 0.001 | 61.22 | < 0.001 | < 0.001 |
|  | **Group*Paradigm** | 1 | 19 | 2.03 | 0.170 | 0.182 | 1.37 | 0.256 | | 0.248 | | 1.95 | | 0.179 | | 0.175 | | 1.30 | 0.269 | 0.269 | 2.58 | 0.125 | 0.126 |
|  | **Paradigm*Material-Type** | 1 | 19 | 1.62 | 0.218 | 0.220 | 1.28 | 0.273 | | 0.267 | | 1.31 | | 0.267 | | 0.265 | | 1.33 | 0.263 | 0.266 | 1.84 | 0.191 | 0.187 |
|  | **Group*Paradigm*Material-Type** | 1 | 19 | 0.37 | 0.551 | 0.550 | 0.31 | 0.586 | | 0.594 | | 0.31 | | 0.586 | | 0.580 | | 0.28 | 0.604 | 0.610 | 0.56 | 0.465 | 0.460 |
|  | | | | | | | | | | | | | | | | | | | | | | | |
| **HPC (H1-H7) vs. CTRs:**  **Faces**  **(3-way ANOVA)** | **Group** | 1 | 19 | 4.35 | 0.051 | 0.052 | 2.16 | 0.158 | 0.160 | | 2.40 | | 0.138 | | 0.141 | | 3.52 | | 0.076 | 0.081 | 4.44 | 0.049 | 0.048 |
|  | **Process** | 1 | 19 | 12.48 | 0.002 | 0.002 | 11.97 | 0.003 | 0.002 | | 13.11 | | 0.002 | | 0.002 | | 14.13 | | 0.001 | 0.001 | 13.05 | 0.002 | 0.002 |
|  | **Group*Process** | 1 | 19 | 0.70 | 0.413 | 0.412 | 0.89 | 0.359 | 0.362 | | 0.91 | | 0.353 | | 0.360 | | 1.65 | | 0.214 | 0.216 | 0.59 | 0.452 | 0.475 |
|  | **Paradigm** | 1 | 19 | 0.05 | 0.829 | 0.840 | 0.06 | 0.809 | 0.798 | | 0.19 | | 0.672 | | 0.680 | | 0.40 | | 0.533 | 0.545 | 0.08 | 0.778 | 0.775 |
|  | **Group*Paradigm** | 1 | 19 | 7.98 | 0.011 | 0.009 | 4.07 | 0.058 | 0.061 | | 3.96 | | 0.061 | | 0.061 | | 2.41 | | 0.137 | 0.135 | 5.41 | 0.031 | 0.031 |
|  | **Process*Paradigm** | 1 | 19 | 18.91 | < 0.001 | 0.001 | 30.36 | < 0.001 | < 0.001 | | 24.12 | | < 0.001 | | 0.001 | | 19.90 | | < 0.001 | < 0.001 | 22.60 | < 0.001 | < 0.001 |
|  | **Group*Process*Paradigm** | 1 | 19 | 0.05 | 0.827 | 0.832 | < 0.05 | 0.978 | 0.975 | | 0.07 | | 0.801 | | 0.805 | | 0.05 | | 0.817 | 0.815 | 0.17 | 0.688 | 0.700 |
|  |  |  |  |  |  |  |  |  |  |  |  |  |  |  |  |  |  |  |  |  |  |  |  |
| **HPC (H1-H7) vs. CTRs:**  **Scenes**  **(3-way ANOVA)** | **Group** | 1 | 19 | 19.01 | < 0.001 | 0.001 | 18.87 | < 0.001 | < 0.001 | | 16.86 | | 0.001 | | 0.001 | | 19.91 | | < 0.001 | 0.001 | 16.30 | 0.001 | 0.001 |
|  | **Process** | 1 | 19 | 1.25 | 0.277 | 0.271 | 0.53 | 0.476 | 0.469 | | 0.85 | | 0.369 | | 0.353 | | 1.32 | | 0.264 | 0.273 | 1.02 | 0.324 | 0.318 |
|  | **Group*Process** | 1 | 19 | 0.70 | 0.412 | 0.418 | 0.20 | 0.660 | 0.658 | | 0.38 | | 0.543 | | 0.546 | | 0.77 | | 0.392 | 0.382 | 0.52 | 0.480 | 0.480 |
|  | **Paradigm** | 1 | 19 | 0.95 | 0.341 | 0.346 | 1.06 | 0.317 | 0.323 | | 1.17 | | 0.292 | | 0.293 | | 0.33 | | 0.571 | 0.560 | 1.10 | 0.308 | 0.313 |
|  | **Group*Paradigm** | 1 | 19 | 0.27 | 0.612 | 0.606 | 0.38 | 0.546 | 0.544 | | 0.38 | | 0.545 | | 0.555 | | < 0.05 | | 0.876 | 0.873 | 0.30 | 0.591 | 0.600 |
|  | **Process*Paradigm** | 1 | 19 | 87.38 | < 0.001 | < 0.001 | 100.91 | < 0.001 | < 0.001 | | 83.52 | | < 0.001 | | < 0.001 | | 103.18 | | < 0.001 | < 0.001 | 71.53 | < 0.001 | < 0.001 |
|  | **Group*Process*Paradigm** | 1 | 19 | 0.33 | 0.575 | 0.573 | 0.00 | 0.946 | 0.942 | | 0.06 | | 0.816 | | 0.814 | | 0.37 | | 0.552 | 0.552 | 0.10 | 0.760 | 0.751 |

ANOVAs (between-participants independent variables: Group (HPC lesion (H1-H7) vs. CTRs); within-participants independent variables: Paradigm (ROC vs. RDP); Process (Familiarity vs. Recollection); Material-Type (Faces vs. Scenes)) on (recollection and familiarity) estimates for MH (focal PRC lesion) and CTRs for faces and scenes in the ROC and the RDP paradigms. These missing values were imputed using “Multiple Imputation with Chained Equations” implemented in the R function “mice”. Five imputations were created (MICE 1-5). shaded cells: significant (p < 0.05) main effects and interactions of interest. ‘p-perm’: p-values calculated from 5000 permutations.

## Supplementary table 4: Categorical Analyses: MH vs HPC patients

| **Linear Model – Fixed Effects** | **Effects/Interactions** | **df1** | **df2** | **F** | **P** | **p-perm** |
| --- | --- | --- | --- | --- | --- | --- |
| Group, Paradigm, Process, Material-Type | Group | 1 | 6 | 1.12 | 0.330 | 0.320 |
|  | Process | 1 | 6 | 5.25 | 0.062 | 0.066 |
|  | Group*Process | 1 | 6 | 7.95 | 0.030 | 0.034 |
|  | Material-Type | 1 | 6 | 1.87 | 0.221 | 0.221 |
|  | Group*Material-Type | 1 | 6 | 0.82 | 0.400 | 0.390 |
|  | Paradigm | 1 | 6 | < 0.05 | 0.954 | 0.954 |
|  | Group*Paradigm | 1 | 6 | < 0.05 | 0.896 | 0.890 |
|  | Process*Material-Type | 1 | 6 | 0.14 | 0.722 | 0.711 |
|  | Group*Process*Material-Type | 1 | 6 | 0.07 | 0.800 | 0.792 |
|  | Process*Paradigm | 1 | 6 | 2.49 | 0.166 | 0.165 |
|  | Group*Process*Paradigm | 1 | 6 | 0.14 | 0.725 | 0.714 |
|  | Material-Type*Paradigm | 1 | 6 | 0.22 | 0.653 | 0.639 |
|  | Group*Material-Type*Paradigm | 1 | 6 | 0.11 | 0.748 | 0.724 |
|  | Process*Material-Type*Paradigm | 1 | 6 | 0.38 | 0.561 | 0.559 |
|  | Group*Process*Material-Type*Paradigm | 1 | 6 | 0.11 | 0.752 | 0.737 |
|  | | | | | | |
| Group (Recollection) | Group | 1 | 6 | 0.42 | 0.542 | 0.622 |
| Group (Familiarity) | Group | 1 | 6 | 8.50 | 0.027 | 0.119 |

We added both MH and the 7 HPC cases (H1-7) to a single linear model, which was fit to patients’ Z-scores relative to CTRs. The model included fixed effects of: Group (PRC vs. HPC lesion); Paradigm (ROC vs. RDP); Process (Recollection vs. Familiarity) and Material-Type (Faces vs. Scenes); shaded cells: significant (p < 0.05) main effects and interactions of interest. ‘p-perm’: p-values calculated from 5000 permutations using the “aovperm” function in R.

## Supplementary table 5: Continuous Analyses : Separate models for HPC / ERC / PRC / PHC

| **Model** | **Effect/Interaction** | **Df1** | **Df2** | **F** | **p** |
| --- | --- | --- | --- | --- | --- |
| ROI: HPC  **Predictors:**  ROI volume  Process (Recollection vs. Familiarity)  Material-Type (Faces vs. Scenes)  Paradigm (ROC vs. RDP);  **Outcome variable:**  (Recollection and Familiarity) estimates | Volume | 1 | 7 | 0.35 | 0.571 |
|  | Process | 1 | 49 | 7.17 | 0.010 |
|  | Material-Type | 1 | 49 | 0.59 | 0.445 |
|  | Paradigm | 1 | 49 | 0.63 | 0.431 |
|  | Volume*Process | 1 | 49 | 7.53 | 0.008 |
|  | Volume*Material-Type | 1 | 49 | 0.30 | 0.589 |
|  | Process*Material-Type | 1 | 49 | < 0.05 | 0.921 |
|  | Volume*Paradigm | 1 | 49 | 0.68 | 0.413 |
|  | Process*Paradigm | 1 | 49 | 0.52 | 0.474 |
|  | Material-Type*Paradigm | 1 | 49 | 1.01 | 0.320 |
|  | Volume*Process*Material-Type | 1 | 49 | 0.10 | 0.750 |
|  | Volume*Process*Paradigm | 1 | 49 | < 0.05 | 0.876 |
|  | Volume*Material-Type*Paradigm | 1 | 49 | 0.54 | 0.464 |
|  | Process*Material-Type*Paradigm | 1 | 49 | < 0.05 | 0.876 |
|  | Volume*Process*Material-Type*Paradigm | 1 | 49 | < 0.05 | 0.913 |
| ROI: PRC  **Predictors:**  ROI volume  Process (Recollection vs. Familiarity)  Material-Type (Faces vs. Scenes)  Paradigm (ROC vs. RDP);  **Outcome variable:**  (Recollection and Familiarity) estimates | Volume | 1 | 7 | 0.72 | 0.425 |
|  | Process | 1 | 49 | < 0.05 | 0.947 |
|  | Material-Type | 1 | 49 | 10.79 | 0.002 |
|  | Paradigm | 1 | 49 | 0.18 | 0.669 |
|  | Volume*Process | 1 | 49 | 0.08 | 0.784 |
|  | Volume*Material-Type | 1 | 49 | 2.52 | 0.119 |
|  | Process*Material-Type | 1 | 49 | 1.78 | 0.188 |
|  | Volume*Paradigm | 1 | 49 | 0.45 | 0.507 |
|  | Process*Paradigm | 1 | 49 | 3.10 | 0.085 |
|  | Material-Type*Paradigm | 1 | 49 | 0.98 | 0.326 |
|  | Volume*Process*Material-Type | 1 | 49 | 0.85 | 0.361 |
|  | Volume*Process*Paradigm | 1 | 49 | 0.18 | 0.670 |
|  | Volume*Material-Type*Paradigm | 1 | 49 | 0.37 | 0.547 |
|  | Process*Material-Type*Paradigm | 1 | 49 | 2.58 | 0.115 |
|  | Volume*Process*Material-Type*Paradigm | 1 | 49 | 2.57 | 0.116 |
| ROI: ERC  **Predictors:**  ROI volume  Process (Recollection vs. Familiarity)  Material-Type (Faces vs. Scenes)  Paradigm (ROC vs. RDP);  **Outcome variable:**  (Recollection and Familiarity) estimates | Volume | 1 | 7 | 0.36 | 0.568 |
|  | Process | 1 | 49 | 0.10 | 0.756 |
|  | Material-Type | 1 | 49 | 2.15 | 0.149 |
|  | Paradigm | 1 | 49 | 0.78 | 0.381 |
|  | Volume*Process | 1 | 49 | < 0.05 | 0.927 |
|  | Volume*Material-Type | 1 | 49 | 0.33 | 0.571 |
|  | Process*Material-Type | 1 | 49 | 0.08 | 0.779 |
|  | Volume*Paradigm | 1 | 49 | 1.48 | 0.230 |
|  | Process*Paradigm | 1 | 49 | 0.93 | 0.341 |
|  | Material-Type*Paradigm | 1 | 49 | < 0.05 | 0.987 |
|  | Volume*Process*Material-Type | 1 | 49 | 0.16 | 0.689 |
|  | Volume*Process*Paradigm | 1 | 49 | 0.19 | 0.662 |
|  | Volume*Material-Type*Paradigm | 1 | 49 | 0.44 | 0.512 |
|  | Process*Material-Type*Paradigm | 1 | 49 | 0.32 | 0.574 |
|  | Volume*Process*Material-Type*Paradigm | 1 | 49 | 0.05 | 0.817 |
| ROI: PHC  **Predictors:**  ROI volume  Process (Recollection vs. Familiarity)  Material-Type (Faces vs. Scenes)  Paradigm (ROC vs. RDP);  **Outcome variable:**  (Recollection and Familiarity) estimates | Volume | 1 | 7 | 9.38 | 0.018 |
|  | Process | 1 | 49 | 1.42 | 0.239 |
|  | Material-Type | 1 | 49 | 2.83 | 0.099 |
|  | Paradigm | 1 | 49 | 0.39 | 0.534 |
|  | Volume*Process | 1 | 49 | 3.63 | 0.062 |
|  | Volume*Material-Type | 1 | 49 | 0.25 | 0.616 |
|  | Process*Material-Type | 1 | 49 | < 0.05 | 0.877 |
|  | Volume*Paradigm | 1 | 49 | 0.81 | 0.373 |
|  | Process*Paradigm | 1 | 49 | 0.34 | 0.565 |
|  | Material-Type*Paradigm | 1 | 49 | 0.37 | 0.546 |
|  | Volume*Process*Material-Type | 1 | 49 | 0.41 | 0.527 |
|  | Volume*Process*Paradigm | 1 | 49 | 1.19 | 0.282 |
|  | Volume*Material-Type*Paradigm | 1 | 49 | < 0.05 | 0.922 |
|  | Process*Material-Type*Paradigm | 1 | 49 | 0.94 | 0.336 |
|  | Volume*Process*Material-Type*Paradigm | 1 | 49 | 3.33 | 0.074 |

Results from fitting a series of linear mixed-effects models, for recollection and familiarity estimates, across all patients (n=9). One model was fit for each ROI: HPC / ERC / PRC / PHC. Each model involved the following predictors: ROI volume (averaged across hemispheres); Paradigm (ROC vs. RDP); Process (Recollection vs. Familiarity); Material Type (Faces vs. Scenes). **Key: ROI:** region of interest; **PHC:** parahippocampal cortex; **PRC:** perirhinal cortex; **HPC:** hippocampus; **ERC:** entorhinal cortex; shaded cells: significant (p < 0.05) main effects and interactions of interest.

## Supplementary table 6a: Continuous Analyses : Separate models for HPC / PHC x Recollection / Familiarity

| **Model** | **Effect/Interaction** | **Df1** | **Df2** | **F** | **p** |
| --- | --- | --- | --- | --- | --- |
| ROI: HPC  **Predictors:**  ROI volume  Material-Type (Faces vs. Scenes)  Paradigm (ROC vs. RDP)  **Outcome variable:**  Recollection estimates | Volume | 1 | 7 | 6.93 | 0.034 |
|  | Material-Type | 1 | 21 | 0.26 | 0.617 |
|  | Paradigm | 1 | 21 | 1.32 | 0.264 |
|  | Volume *Material-Type | 1 | 21 | < 0.05 | 0.867 |
|  | Volume *Paradigm | 1 | 21 | 0.26 | 0.617 |
|  | Material-Type*Paradigm | 1 | 21 | 0.78 | 0.388 |
|  | Volume *Material-Type*Paradigm | 1 | 21 | 0.23 | 0.639 |
| ROI: HPC  **Predictors:**  ROI volume  Material-Type (Faces vs. Scenes)  Paradigm (ROC vs. RDP)  **Outcome variable:**  Familiarity Estimates | Volume | 1 | 7 | 0.55 | 0.484 |
|  | Material-Type | 1 | 21 | 0.30 | 0.590 |
|  | Paradigm | 1 | 21 | < 0.05 | 0.964 |
|  | Volume*Material-Type | 1 | 21 | 0.30 | 0.593 |
|  | Volume*Paradigm | 1 | 21 | 0.38 | 0.543 |
|  | Material-Type*Paradigm | 1 | 21 | 0.28 | 0.600 |
|  | Volume*Material-Type*Paradigm | 1 | 21 | 0.28 | 0.600 |
| ROI: PHC  **Predictors:**  ROI volume  Material-Type (Faces vs. Scenes)  Paradigm (ROC vs. RDP)  **Outcome variable:**  Recollection Estimates | Volume | 1 | 7 | 0.79 | 0.405 |
|  | Material-Type | 1 | 21 | 1.59 | 0.221 |
|  | Paradigm | 1 | 21 | < 0.05 | 0.969 |
|  | Volume*Material-Type | 1 | 21 | < 0.05 | 0.913 |
|  | Volume*Paradigm | 1 | 21 | 2.70 | 0.115 |
|  | Material-Type*Paradigm | 1 | 21 | 0.09 | 0.767 |
|  | Volume*Material-Type*Paradigm | 1 | 21 | 2.04 | 0.168 |
| ROI: PHC  **Predictors:**  ROI volume  Material-Type (Faces vs. Scenes)  Paradigm (ROC vs. RDP)  **Outcome variable:**  Familiarity Estimates | Volume | 1 | 28 | 14.09 | 0.001 |
|  | Material-Type | 1 | 28 | 1.56 | 0.222 |
|  | Paradigm | 1 | 28 | 0.67 | 0.420 |
|  | Volume*Material-Type | 1 | 28 | 0.60 | 0.445 |
|  | Volume*Paradigm | 1 | 28 | < 0.05 | 0.898 |
|  | Material-Type*Paradigm | 1 | 28 | 1.15 | 0.292 |
|  | Volume*Material-Type*Paradigm | 1 | 28 | 1.71 | 0.202 |

Results from fitting a series of linear mixed-effects models, across all patients (n=9). One model was fit for each of the two ROIs of interest (HPC, PHC), separately for Recollection and Familiarity estimates. Each model involved the following predictors: ROI volume (averaged across hemispheres); Paradigm (ROC vs. RDP); Material Type (Faces vs. Scenes). **Key: ROI:** region of interest; **PHC:** parahippocampal cortex; **HPC:** hippocampus; shaded cells: significant (p < 0.05) main effects and interactions of interest.

## Supplementary table 6b: Continuous Analyses : Separate models for HPC / PHC x Recollection / Familiarity (+ Age)

| **Model** | **Effect/Interaction** | **Df1** | **Df2** | **F** | **p** |
| --- | --- | --- | --- | --- | --- |
| ROI: HPC  **Predictors:**  ROI volume  Material-Type (Faces vs. Scenes)  Paradigm (ROC vs. RDP)  Age  **Outcome variable:**  Recollection estimates | Volume | 1 | 6 | 4.51 | 0.078 |
|  | Material-Type | 1 | 21 | 0.26 | 0.617 |
|  | Paradigm | 1 | 21 | 1.32 | 0.264 |
|  | Age | 1 | 6 | < 0.05 | 0.855 |
|  | Volume *Material-Type | 1 | 21 | < 0.05 | 0.867 |
|  | Volume *Paradigm | 1 | 21 | 0.26 | 0.617 |
|  | Material-Type*Paradigm | 1 | 21 | 0.78 | 0.388 |
|  | Volume *Material-Type*Paradigm | 1 | 21 | 0.23 | 0.639 |
| ROI: HPC  **Predictors:**  ROI volume  Material-Type (Faces vs. Scenes)  Paradigm (ROC vs. RDP)  Age  **Outcome variable:**  Familiarity Estimates | Volume | 1 | 6 | 0.84 | 0.394 |
|  | Material-Type | 1 | 21 | 0.30 | 0.590 |
|  | Paradigm | 1 | 21 | < 0.05 | 0.964 |
|  | Age | 1 | 6 | 0.42 | 0.541 |
|  | Volume *Material-Type | 1 | 21 | 0.30 | 0.593 |
|  | Volume *Paradigm | 1 | 21 | 0.38 | 0.543 |
|  | Material-Type*Paradigm | 1 | 21 | 0.28 | 0.600 |
|  | Volume *Material-Type*Paradigm | 1 | 21 | 0.28 | 0.600 |
| ROI: PHC  **Predictors:**  ROI volume  Material-Type (Faces vs. Scenes)  Paradigm (ROC vs. RDP)  Age  **Outcome variable:**  Recollection Estimates | Volume | 1 | 6 | 0.36 | 0.569 |
|  | Material-Type | 1 | 21 | 1.59 | 0.221 |
|  | Paradigm | 1 | 21 | < 0.05 | 0.969 |
|  | Age | 1 | 6 | 0.54 | 0.492 |
|  | Volume *Material-Type | 1 | 21 | < 0.05 | 0.913 |
|  | Volume *Paradigm | 1 | 21 | 2.70 | 0.115 |
|  | Material-Type*Paradigm | 1 | 21 | 0.09 | 0.767 |
|  | Volume *Material-Type*Paradigm | 1 | 21 | 2.04 | 0.168 |
| ROI: PHC  **Predictors:**  ROI volume  Material-Type (Faces vs. Scenes)  Paradigm (ROC vs. RDP)  Age  **Outcome variable:**  Familiarity Estimates | Volume | 1 | 27 | 14.07 | 0.001 |
|  | Material-Type | 1 | 27 | 1.53 | 0.227 |
|  | Paradigm | 1 | 27 | 0.66 | 0.425 |
|  | Age | 1 | 27 | 0.46 | 0.505 |
|  | Volume *Material-Type | 1 | 27 | 0.59 | 0.449 |
|  | Volume *Paradigm | 1 | 27 | < 0.05 | 0.899 |
|  | Material-Type*Paradigm | 1 | 27 | 1.13 | 0.297 |
|  | Volume*Material-Type*Paradigm | 1 | 27 | 1.67 | 0.207 |

Results from fitting a series of linear mixed-effects models, across all patients (n=9). One model was fit for each of the two ROIs of interest (HPC, PHC), separately for Recollection and Familiarity estimates. Each model involved the following predictors: ROI volume (averaged across hemispheres); Paradigm (ROC vs. RDP); Material Type (Faces vs. Scenes), and Age. **Key: ROI:** region of interest; **PHC:** parahippocampal cortex; **HPC:** hippocampus; shaded cells: significant (p < 0.05) main effects and interactions of interest.

## Supplementary table 7: Continuous Analyses: Linear regression of Average HPC / PRC / ERC / PHC volume against Average Recollection and Familiarity estimates

| **Average**  **volume**  **(z)** | **Estimate (z)** | | | | | |
| --- | --- | --- | --- | --- | --- | --- |
|  | **Recollection** | | | **Familiarity** | | |
|  | **R^2^** | **p** | **p-perm** | **R^2^** | **p** | **p-perm** |
| **HPC** | 0.50 | 0.034 | 0.028 | 0.07 | 0.484 | 0.483 |
| **PRC** | < 0.05 | 0.572 | 0.569 | 0.08 | 0.474 | 0.466 |
| **ERC** | < 0.05 | 0.580 | 0.553 | < 0.05 | 0.697 | 0.676 |
| **PHC** | 0.10 | 0.405 | 0.391 | 0.76 | 0.002 | 0.002 |

Double dissociation in brain-behavior relationships between HPC volume – Recollection and PHC volume – Familiarity across patients; **key: ERC:** entorhinal cortex; **HPC:** hippocampus; **PHC:** parahippocampal cortex; **PRC:** perirhinal cortex; **Z:** volumes are expressed as Z-scores, based on the mean and standard deviation of the volumes of the 48 CTRs whose MTL structures were manually delineated (see Argyropoulos et al. (2019) for details); Familiarity and Recollection estimates are expressed as Z-scores, based on the mean and standard deviation of the CTRs that completed the two tasks; p-perm: p-values were calculated from 5000 permutations using the “lmperm” function in R; shaded cells: significant (p < 0.05) main effects and interactions of interest.

## Supplementary table 8: Continuous Analyses: HPC and PHC volumes in a single model

| **Model** | **Effect/Interaction** | **Df1** | **Df2** | **F** | **p** |
| --- | --- | --- | --- | --- | --- |
| **‘ROI model’**  **Predictors:**  ROI average volume (HPC vs. PHC)  Process (Recollection vs. Familiarity)  Material-Type (Faces vs. Scenes)  Paradigm (ROC vs. RDP);  **Outcome variable:** (Recollection and Familiarity) estimates | HPC | 1 | 5 | 2.15 | 0.203 |
|  | PHC | 1 | 5 | 3.47 | 0.121 |
|  | Process | 1 | 35 | 0.07 | 0.787 |
|  | Material-Type | 1 | 35 | 0.06 | 0.811 |
|  | Paradigm | 1 | 35 | 1.59 | 0.215 |
|  | HPC*PHC | 1 | 5 | 0.69 | 0.445 |
|  | HPC*Process | 1 | 35 | 0.29 | 0.594 |
|  | PHC*Process | 1 | 35 | 0.56 | 0.459 |
|  | HPC*Material-Type | 1 | 35 | < 0.05 | 0.899 |
|  | PHC*Material-Type | 1 | 35 | < 0.05 | 0.960 |
|  | Process*Material-Type | 1 | 35 | 3.46 | 0.071 |
|  | HPC*Paradigm | 1 | 35 | 2.05 | 0.161 |
|  | PHC*Paradigm | 1 | 35 | 1.04 | 0.315 |
|  | Process*Paradigm | 1 | 35 | 1.99 | 0.167 |
|  | Material-Type*Paradigm | 1 | 35 | 2.42 | 0.129 |
|  | HPC*PHC*Process | 1 | 35 | 0.15 | 0.698 |
|  | HPC*PHC*Material-Type | 1 | 35 | < 0.05 | 0.887 |
|  | HPC*Process*Material-Type | 1 | 35 | 3.75 | 0.061 |
|  | PHC*Process*Material-Type | 1 | 35 | 4.08 | 0.051 |
|  | HPC*PHC*Paradigm | 1 | 35 | 1.63 | 0.210 |
|  | HPC*Process*Paradigm | 1 | 35 | 2.47 | 0.125 |
|  | PHC*Process*Paradigm | 1 | 35 | 3.20 | 0.082 |
|  | HPC*Material-Type*Paradigm | 1 | 35 | 2.11 | 0.155 |
|  | PHC*Material-Type*Paradigm | 1 | 35 | 1.62 | 0.212 |
|  | Process*Material-Type*Paradigm | 1 | 35 | 1.72 | 0.198 |
|  | HPC*PHC*Process*Material-Type | 1 | 35 | 3.54 | 0.068 |
|  | HPC*PHC*Process*Paradigm | 1 | 35 | 2.29 | 0.139 |
|  | HPC*PHC*Material-Type*Paradigm | 1 | 35 | 1.54 | 0.224 |
|  | HPC*Process*Material-Type*Paradigm | 1 | 35 | 1.26 | 0.269 |
|  | PHC*Process*Material-Type*Paradigm | 1 | 35 | 2.22 | 0.146 |
|  | HPC*PHC*Process*Material-Type*Paradigm | 1 | 35 | 0.96 | 0.334 |

Results from a linear mixed-effects model, where the volumes of the 2 ROIs of interest (HPC, PHC), averaged across hemispheres, are entered as predictors. **Key: ROI:** region of interest; **PHC:** parahippocampal cortex; **HPC:** hippocampus; shaded cells: significant (p < 0.05) main effects and interactions of interest.

## Supplementary table 9: Continuous Analyses: ROI vs MTL model

We compared a combined model with HPC, PHC, ERC, and PRC volumes (‘ROI model’) with a single model (‘MTL model’) that includes only total MTL volume (sum of the four ROIs). The model fit index of AIC prefers the ROI model (AIC=180.13) over the MTL model (AIC=222.56; χ^2^ = 106.43, p < 0.001), while the two models have similar BICs (ROI model: BIC = 293.56; MTL model: BIC = 263.54).

| **Model** | **Effect/Interaction** | **Df1** | **Df2** | **F** | **p** |
| --- | --- | --- | --- | --- | --- |
| **‘ROI model’**  **Predictors:**  ROI average volume (HPC vs. PHC vs. ERC vs. PRC)  Process (Recollection vs. Familiarity)  Material-Type (Faces vs. Scenes)  Paradigm (ROC vs. RDP);  **Outcome variable:** (Recollection and Familiarity) estimates | HPC | 1 | 3 | 1.08 | 0.375 |
|  | PHC | 1 | 3 | 1.43 | 0.317 |
|  | ERC | 1 | 3 | 0.20 | 0.683 |
|  | PRC | 1 | 3 | 2.24 | 0.232 |
|  | Process | 1 | 21 | 0.23 | 0.637 |
|  | Material-Type | 1 | 21 | 0.06 | 0.807 |
|  | Paradigm | 1 | 21 | 0.27 | 0.610 |
|  | HPC*PHC | 1 | 3 | 0.09 | 0.784 |
|  | HPC*Process | 1 | 21 | < 0.05 | 0.900 |
|  | PHC*Process | 1 | 21 | 2.11 | 0.161 |
|  | ERC*Process | 1 | 21 | 1.44 | 0.243 |
|  | PRC*Process | 1 | 21 | 0.31 | 0.586 |
|  | HPC*Material-Type | 1 | 21 | 0.53 | 0.473 |
|  | PHC*Material-Type | 1 | 21 | 0.66 | 0.427 |
|  | ERC*Material-Type | 1 | 21 | 1.42 | 0.247 |
|  | PRC*Material-Type | 1 | 21 | 5.37 | 0.031 |
|  | Process*Material-Type | 1 | 21 | 11.38 | 0.003 |
|  | HPC*Paradigm | 1 | 21 | 0.82 | 0.376 |
|  | PHC*Paradigm | 1 | 21 | 0.16 | 0.694 |
|  | ERC*Paradigm | 1 | 21 | 0.82 | 0.376 |
|  | PRC*Paradigm | 1 | 21 | 0.10 | 0.759 |
|  | Process*Paradigm | 1 | 21 | 6.61 | 0.018 |
|  | Material-Type*Paradigm | 1 | 21 | 0.93 | 0.345 |
|  | HPC*PHC*Process | 1 | 21 | 1.37 | 0.255 |
|  | HPC*PHC*Material-Type | 1 | 21 | 0.49 | 0.490 |
|  | HPC*Process*Material-Type | 1 | 21 | 12.36 | 0.002 |
|  | PHC*Process*Material-Type | 1 | 21 | 13.85 | 0.001 |
|  | ERC*Process*Material-Type | 1 | 21 | 8.43 | 0.008 |
|  | PRC*Process*Material-Type | 1 | 21 | 4.93 | 0.038 |
|  | HPC*PHC*Paradigm | 1 | 21 | 0.23 | 0.635 |
|  | HPC*Process*Paradigm | 1 | 21 | 7.58 | 0.012 |
|  | PHC*Process*Paradigm | 1 | 21 | 9.20 | 0.006 |
|  | ERC*Process*Paradigm | 1 | 21 | 4.66 | 0.043 |
|  | PRC*Process*Paradigm | 1 | 21 | 1.91 | 0.181 |
|  | HPC*Material-Type*Paradigm | 1 | 21 | 0.89 | 0.357 |
|  | PHC*Material-Type*Paradigm | 1 | 21 | 0.34 | 0.564 |
|  | ERC*Material-Type*Paradigm | 1 | 21 | 0.92 | 0.348 |
|  | PRC*Material-Type*Paradigm | 1 | 21 | 1.39 | 0.252 |
|  | Process*Material-Type*Paradigm | 1 | 21 | 1.33 | 0.261 |
|  | HPC*PHC*Process*Material-Type | 1 | 21 | 13.28 | 0.002 |
|  | HPC*PHC*Process*Paradigm | 1 | 21 | 7.88 | 0.011 |
|  | HPC*PHC*Material-Type*Paradigm | 1 | 21 | 0.17 | 0.687 |
|  | HPC*Process*Material-Type*Paradigm | 1 | 21 | 1.58 | 0.222 |
|  | PHC*Process*Material-Type*Paradigm | 1 | 21 | 2.69 | 0.116 |
|  | ERC*Process*Material-Type*Paradigm | 1 | 21 | 0.13 | 0.725 |
|  | PRC*Process*Material-Type*Paradigm | 1 | 21 | 4.53 | 0.045 |
|  | HPC*PHC*Process*Material-Type*Paradigm | 1 | 21 | 1.21 | 0.284 |
| **‘MTL model’**  **Predictors:**  MTL volume (HPC + ERC + PRC + PHC volume)  Process (Recollection vs. Familiarity)  Material-Type (Faces vs. Scenes)  Paradigm (ROC vs. RDP);  **Outcome variable:** (Recollection and Familiarity) estimates | MTL | 1 | 7 | 3.37 | 0.109 |
|  | Process | 1 | 49 | 0.94 | 0.338 |
|  | Material-Type | 1 | 49 | 0.74 | 0.393 |
|  | Paradigm | 1 | 49 | 0.32 | 0.577 |
|  | MTL*Process | 1 | 49 | 0.79 | 0.377 |
|  | MTL*Material-Type | 1 | 49 | < 0.05 | 0.845 |
|  | Process*Material-Type | 1 | 49 | < 0.05 | 0.927 |
|  | MTL*Paradigm | 1 | 49 | 0.40 | 0.530 |
|  | Process*Paradigm | 1 | 49 | 0.08 | 0.784 |
|  | Material-Type*Paradigm | 1 | 49 | 0.30 | 0.589 |
|  | MTL*Process*Material-Type | 1 | 49 | 0.06 | 0.805 |
|  | MTL*Process*Paradigm | 1 | 49 | 0.22 | 0.643 |
|  | MTL*Material-Type*Paradigm | 1 | 49 | 0.09 | 0.771 |
|  | Process*Material-Type*Paradigm | 1 | 49 | 0.08 | 0.781 |
|  | MTL*Process*Material-Type*Paradigm | 1 | 49 | < 0.05 | 0.943 |

Results from two linear mixed-effects models: the ‘ROI model’, where the volumes of the 4 ROIs (HPC, ERC, PRC, PHC), averaged across hemispheres, are entered as predictors; the ‘MTL model’, where ‘MTL volume’ (the sum of HPC, ERC, PRC, and PHC volume) is entered as a predictor instead (along with the rest of the predictors and nuisance covariates). **Key: ROI:** region of interest; **PHC:** parahippocampal cortex; **PRC:** perirhinal cortex; **HPC:** hippocampus; **ERC:** entorhinal cortex; shaded cells: significant (p < 0.05) main effects and interactions of interest.

## Supplementary table 10: Continuous Analyses : Separate models for L / R HPC / ERC / PRC / PHC

| **Model** | **Effect/Interaction** | **Df1** | **Df2** | **F** | **p** |
| --- | --- | --- | --- | --- | --- |
| ROI: Left HPC  **Predictors:**  ROI volume  Process (Recollection vs. Familiarity)  Material-Type (Faces vs. Scenes)  Paradigm (ROC vs. RDP);  **Outcome variable:** (Recollection and Familiarity) estimates | Volume | 1 | 7 | 0.27 | 0.622 |
|  | Process | 1 | 49 | 6.92 | 0.011 |
|  | Material-Type | 1 | 49 | 0.57 | 0.453 |
|  | Paradigm | 1 | 49 | 0.47 | 0.494 |
|  | Volume*Process | 1 | 49 | 7.62 | 0.008 |
|  | Volume*Material-Type | 1 | 49 | 0.68 | 0.415 |
|  | Process*Material-Type | 1 | 49 | 0.66 | 0.420 |
|  | Volume*Paradigm | 1 | 49 | 0.53 | 0.468 |
|  | Process*Paradigm | 1 | 49 | 1.40 | 0.242 |
|  | Material-Type*Paradigm | 1 | 49 | 0.56 | 0.459 |
|  | Volume*Process*Material-Type | 1 | 49 | 0.16 | 0.692 |
|  | Volume*Process*Paradigm | 1 | 49 | 0.04 | 0.846 |
|  | Volume*Material-Type*Paradigm | 1 | 49 | 0.17 | 0.683 |
|  | Process*Material-Type*Paradigm | 1 | 49 | 0.00 | 0.997 |
|  | Volume*Process*Material-Type*Paradigm | 1 | 49 | 0.12 | 0.732 |
| ROI: Right HPC  **Predictors:**  ROI volume  Process (Recollection vs. Familiarity)  Material-Type (Faces vs. Scenes)  Paradigm (ROC vs. RDP);  **Outcome variable:** (Recollection and Familiarity) estimates | Volume | 1 | 7 | 0.35 | 0.574 |
|  | Process | 1 | 49 | 5.30 | 0.026 |
|  | Material-Type | 1 | 49 | 1.11 | 0.297 |
|  | Paradigm | 1 | 49 | 0.61 | 0.440 |
|  | Volume*Process | 1 | 49 | 5.42 | 0.024 |
|  | Volume*Material-Type | 1 | 49 | 0.03 | 0.862 |
|  | Process*Material-Type | 1 | 49 | 0.32 | 0.573 |
|  | Volume*Paradigm | 1 | 49 | 0.65 | 0.424 |
|  | Process*Paradigm | 1 | 49 | 0.13 | 0.716 |
|  | Material-Type*Paradigm | 1 | 49 | 1.47 | 0.232 |
|  | Volume*Process*Material-Type | 1 | 49 | 1.05 | 0.311 |
|  | Volume*Process*Paradigm | 1 | 49 | 0.25 | 0.618 |
|  | Volume*Material-Type*Paradigm | 1 | 49 | 0.94 | 0.336 |
|  | Process*Material-Type*Paradigm | 1 | 49 | 0.15 | 0.701 |
|  | Volume*Process*Material-Type*Paradigm | 1 | 49 | 0.02 | 0.880 |
| ROI: Left PRC  **Predictors:**  ROI volume  Process (Recollection vs. Familiarity)  Material-Type (Faces vs. Scenes)  Paradigm (ROC vs. RDP);  **Outcome variable:** (Recollection and Familiarity) estimates | Volume | 1 | 7 | 0.14 | 0.718 |
|  | Process | 1 | 49 | 1.10 | 0.299 |
|  | Material-Type | 1 | 49 | 5.01 | 0.030 |
|  | Paradigm | 1 | 49 | 0.57 | 0.452 |
|  | Volume*Process | 1 | 49 | 1.11 | 0.297 |
|  | Volume*Material-Type | 1 | 49 | 0.08 | 0.782 |
|  | Process*Material-Type | 1 | 49 | 0.13 | 0.722 |
|  | Volume*Paradigm | 1 | 49 | 1.17 | 0.285 |
|  | Process*Paradigm | 1 | 49 | 2.77 | 0.102 |
|  | Material-Type*Paradigm | 1 | 49 | 0.14 | 0.709 |
|  | Volume*Process*Material-Type | 1 | 49 | 0.13 | 0.720 |
|  | Volume*Process*Paradigm | 1 | 49 | 0.14 | 0.709 |
|  | Volume*Material-Type*Paradigm | 1 | 49 | 0.04 | 0.844 |
|  | Process*Material-Type*Paradigm | 1 | 49 | 2.90 | 0.095 |
|  | Volume*Process*Material-Type*Paradigm | 1 | 49 | 2.93 | 0.093 |
| ROI: Right PRC  **Predictors:**  ROI volume  Process (Recollection vs. Familiarity)  Material-Type (Faces vs. Scenes)  Paradigm (ROC vs. RDP);  **Outcome variable:** (Recollection and Familiarity) estimates | Volume | 1 | 7 | 1.43 | 0.271 |
|  | Process | 1 | 49 | 0.02 | 0.885 |
|  | Material-Type | 1 | 49 | 12.36 | 0.001 |
|  | Paradigm | 1 | 49 | 0.01 | 0.922 |
|  | Volume*Process | 1 | 49 | 0.61 | 0.438 |
|  | Volume*Material-Type | 1 | 49 | 2.87 | 0.096 |
|  | Process*Material-Type | 1 | 49 | 2.25 | 0.140 |
|  | Volume*Paradigm | 1 | 49 | 0.08 | 0.776 |
|  | Process*Paradigm | 1 | 49 | 3.63 | 0.063 |
|  | Material-Type*Paradigm | 1 | 49 | 1.24 | 0.272 |
|  | Volume*Process*Material-Type | 1 | 49 | 1.48 | 0.230 |
|  | Volume*Process*Paradigm | 1 | 49 | 0.11 | 0.746 |
|  | Volume*Material-Type*Paradigm | 1 | 49 | 0.61 | 0.438 |
|  | Process*Material-Type*Paradigm | 1 | 49 | 1.32 | 0.256 |
|  | Volume*Process*Material-Type*Paradigm | 1 | 49 | 1.16 | 0.287 |
| ROI: Left ERC  **Predictors:**  ROI volume  Process (Recollection vs. Familiarity)  Material-Type (Faces vs. Scenes)  Paradigm (ROC vs. RDP);  **Outcome variable:** (Recollection and Familiarity) estimates | Volume | 1 | 7 | 0.00 | 0.955 |
|  | Process | 1 | 49 | 0.04 | 0.847 |
|  | Material-Type | 1 | 49 | 3.30 | 0.075 |
|  | Paradigm | 1 | 49 | 0.30 | 0.589 |
|  | Volume*Process | 1 | 49 | 0.01 | 0.922 |
|  | Volume*Material-Type | 1 | 49 | 0.14 | 0.707 |
|  | Process*Material-Type | 1 | 49 | 0.08 | 0.784 |
|  | Volume*Paradigm | 1 | 49 | 0.71 | 0.405 |
|  | Process*Paradigm | 1 | 49 | 0.88 | 0.353 |
|  | Material-Type*Paradigm | 1 | 49 | 0.00 | 0.955 |
|  | Volume*Process*Material-Type | 1 | 49 | 0.27 | 0.606 |
|  | Volume*Process*Paradigm | 1 | 49 | 0.46 | 0.501 |
|  | Volume*Material-Type*Paradigm | 1 | 49 | 0.69 | 0.410 |
|  | Process*Material-Type*Paradigm | 1 | 49 | 0.63 | 0.432 |
|  | Volume*Process*Material-Type*Paradigm | 1 | 49 | 0.26 | 0.610 |
| ROI: Right ERC  **Predictors:**  ROI volume  Process (Recollection vs. Familiarity)  Material-Type (Faces vs. Scenes)  Paradigm (ROC vs. RDP);  **Outcome variable:** (Recollection and Familiarity) estimates | Volume | 1 | 7 | 1.87 | 0.214 |
|  | Process | 1 | 49 | 0.22 | 0.638 |
|  | Material-Type | 1 | 49 | 2.17 | 0.147 |
|  | Paradigm | 1 | 49 | 0.95 | 0.333 |
|  | Volume*Process | 1 | 49 | 0.09 | 0.768 |
|  | Volume*Material-Type | 1 | 49 | 0.44 | 0.509 |
|  | Process*Material-Type | 1 | 49 | 0.24 | 0.629 |
|  | Volume*Paradigm | 1 | 49 | 1.88 | 0.176 |
|  | Process*Paradigm | 1 | 49 | 1.71 | 0.197 |
|  | Material-Type*Paradigm | 1 | 49 | 0.08 | 0.777 |
|  | Volume*Process*Material-Type | 1 | 49 | 0.03 | 0.868 |
|  | Volume*Process*Paradigm | 1 | 49 | 0.00 | 0.962 |
|  | Volume*Material-Type*Paradigm | 1 | 49 | 0.09 | 0.767 |
|  | Process*Material-Type*Paradigm | 1 | 49 | 0.08 | 0.780 |
|  | Volume*Process*Material-Type*Paradigm | 1 | 49 | 0.03 | 0.874 |
| ROI: Left PHC  **Predictors:**  ROI volume  Process (Recollection vs. Familiarity)  Material-Type (Faces vs. Scenes)  Paradigm (ROC vs. RDP);  **Outcome variable:** (Recollection and Familiarity) estimates | Volume | 1 | 7 | 5.29 | 0.055 |
|  | Process | 1 | 49 | 0.71 | 0.402 |
|  | Material-Type | 1 | 49 | 2.73 | 0.105 |
|  | Paradigm | 1 | 49 | 0.86 | 0.359 |
|  | Volume*Process | 1 | 49 | 1.96 | 0.168 |
|  | Volume*Material-Type | 1 | 49 | 0.20 | 0.653 |
|  | Process*Material-Type | 1 | 49 | 0.92 | 0.343 |
|  | Volume*Paradigm | 1 | 49 | 1.56 | 0.217 |
|  | Process*Paradigm | 1 | 49 | 0.13 | 0.722 |
|  | Material-Type*Paradigm | 1 | 49 | 0.82 | 0.371 |
|  | Volume*Process*Material-Type | 1 | 49 | 4.05 | 0.050 |
|  | Volume*Process*Paradigm | 1 | 49 | 1.65 | 0.205 |
|  | Volume*Material-Type*Paradigm | 1 | 49 | 0.24 | 0.626 |
|  | Process*Material-Type*Paradigm | 1 | 49 | 0.42 | 0.521 |
|  | Volume*Process*Material-Type*Paradigm | 1 | 49 | 1.80 | 0.186 |
| ROI: Right PHC  **Predictors:**  ROI volume  Process (Recollection vs. Familiarity)  Material-Type (Faces vs. Scenes)  Paradigm (ROC vs. RDP);  **Outcome variable:** (Recollection and Familiarity) estimates | Volume | 1 | 7 | 6.26 | 0.041 |
|  | Process | 1 | 49 | 0.93 | 0.339 |
|  | Material-Type | 1 | 49 | 4.37 | 0.042 |
|  | Paradigm | 1 | 49 | 0.05 | 0.821 |
|  | Volume*Process | 1 | 49 | 3.73 | 0.059 |
|  | Volume*Material-Type | 1 | 49 | 0.20 | 0.657 |
|  | Process*Material-Type | 1 | 49 | 1.22 | 0.274 |
|  | Volume*Paradigm | 1 | 49 | 0.20 | 0.655 |
|  | Process*Paradigm | 1 | 49 | 1.32 | 0.256 |
|  | Material-Type*Paradigm | 1 | 49 | 0.22 | 0.643 |
|  | Volume*Process*Material-Type | 1 | 49 | 0.31 | 0.580 |
|  | Volume*Process*Paradigm | 1 | 49 | 0.52 | 0.473 |
|  | Volume*Material-Type*Paradigm | 1 | 49 | 0.05 | 0.822 |
|  | Process*Material-Type*Paradigm | 1 | 49 | 0.53 | 0.469 |
|  | Volume*Process*Material-Type*Paradigm | 1 | 49 | 3.41 | 0.071 |

Results from fitting a series of linear mixed-effects models, for recollection and familiarity estimates, across all patients (n=9). One model was fit for each ROI: L / R HPC / ERC / PRC / PHC. Each model involved the following predictors: ROI volume; Paradigm (ROC vs. RDP); Process (Recollection vs. Familiarity); Material Type (Faces vs. Scenes). **Key: ROI:** region of interest; **PHC:** parahippocampal cortex; **PRC:** perirhinal cortex; **HPC:** hippocampus; **ERC:** entorhinal cortex; shaded cells: significant (p < 0.05) main effects and interactions of interest.

## Supplementary table 11: Categorical Analyses: MH vs. CTRs (including Words)

| **Analysis** | **Main Effect / Interaction** | **df1** | **df2** | **MICE IMPUTATION** | | | | | | | | | | | | | | |
| --- | --- | --- | --- | --- | --- | --- | --- | --- | --- | --- | --- | --- | --- | --- | --- | --- | --- | --- |
|  |  |  |  | **1** | | | **2** | | | **3** | | | **4** | | | **5** | | |
|  |  |  |  | **F** | **p** | **p-perm** | **F** | **p** | **p-perm** | **F** | **p** | **p-perm** | **F** | **p** | **p-perm** | **F** | **p** | **p-perm** |
| **MH vs CTRs**  **(4-way ANOVA:**  **Group,**  **Process,**  **Material-Type,**  **Paradigm)** | **Group** | 1 | 13 | 7.14 | 0.019 | 0.017 | 6.53 | 0.024 | 0.031 | 6.02 | 0.029 | 0.030 | 6.18 | 0.027 | 0.024 | 6.26 | 0.026 | 0.029 |
|  | **Process** | 1 | 13 | 0.54 | 0.477 | 0.477 | 0.36 | 0.557 | 0.556 | 0.37 | 0.552 | 0.538 | 0.44 | 0.518 | 0.523 | 0.54 | 0.474 | 0.480 |
|  | **Group*Process** | 1 | 13 | 5.46 | 0.036 | 0.033 | 4.05 | 0.065 | 0.070 | 5.70 | 0.033 | 0.037 | 5.35 | 0.038 | 0.040 | 5.92 | 0.030 | 0.032 |
|  | **Material-Type** | 2 | 26 | 3.43 | 0.048 | 0.048 | 2.16 | 0.136 | 0.145 | 2.16 | 0.136 | 0.135 | 2.94 | 0.071 | 0.073 | 2.67 | 0.088 | 0.083 |
|  | **Group*Material-Type** | 2 | 26 | 0.69 | 0.512 | 0.505 | 0.20 | 0.818 | 0.806 | 0.42 | 0.663 | 0.655 | 0.47 | 0.629 | 0.632 | 0.45 | 0.640 | 0.634 |
|  | **Paradigm** | 1 | 13 | 2.62 | 0.130 | 0.127 | 2.76 | 0.121 | 0.126 | 2.69 | 0.125 | 0.128 | 0.91 | 0.357 | 0.358 | 3.84 | 0.072 | 0.067 |
|  | **Group*Paradigm** | 1 | 13 | 0.22 | 0.646 | 0.648 | 0.21 | 0.657 | 0.669 | 0.28 | 0.602 | 0.599 | 0.29 | 0.602 | 0.605 | 0.15 | 0.701 | 0.704 |
|  | **Material-Type*Process** | 2 | 26 | 2.63 | 0.091 | 0.097 | 2.97 | 0.069 | 0.073 | 2.56 | 0.097 | 0.101 | 2.99 | 0.068 | 0.070 | 2.31 | 0.119 | 0.116 |
|  | **Group*Material-Type*Process** | 2 | 26 | 0.21 | 0.809 | 0.817 | 0.34 | 0.713 | 0.706 | 0.14 | 0.872 | 0.863 | 0.27 | 0.763 | 0.767 | 0.17 | 0.845 | 0.842 |
|  | **Paradigm*Process** | 1 | 13 | 10.72 | 0.006 | 0.006 | 18.14 | 0.001 | 0.001 | 9.92 | 0.008 | 0.009 | 11.76 | 0.004 | 0.003 | 10.58 | 0.006 | 0.006 |
|  | **Group*Paradigm*Process** | 1 | 13 | 0.48 | 0.503 | 0.501 | 0.32 | 0.582 | 0.580 | 0.27 | 0.612 | 0.604 | 0.36 | 0.559 | 0.546 | 0.48 | 0.501 | 0.504 |
|  | **Paradigm*Material-Type** | 2 | 26 | 1.45 | 0.253 | 0.245 | 0.85 | 0.437 | 0.430 | 1.24 | 0.307 | 0.299 | 1.71 | 0.200 | 0.204 | 1.91 | 0.168 | 0.162 |
|  | **Group*Paradigm*Material-Type** | 2 | 26 | 1.65 | 0.212 | 0.203 | 0.97 | 0.393 | 0.379 | 1.28 | 0.295 | 0.281 | 1.81 | 0.184 | 0.182 | 1.08 | 0.354 | 0.343 |
|  | **Paradigm*Material-Type*Process** | 2 | 26 | 4.19 | 0.026 | 0.022 | 6.16 | 0.006 | 0.006 | 4.92 | 0.015 | 0.019 | 4.41 | 0.022 | 0.022 | 5.08 | 0.014 | 0.013 |
|  | **Group*Paradigm*Material-Type*Process** | 2 | 26 | 0.08 | 0.924 | 0.926 | 0.06 | 0.945 | 0.941 | 0.10 | 0.906 | 0.905 | 0.16 | 0.852 | 0.853 | 0.06 | 0.941 | 0.940 |
|  | | | | | | | | | | | | | | | | | | |
| **MH vs CTRs**  **Recollection**  **(3-way ANOVA:**  **Group,**  **Material-Type,**  **Paradigm)** | **Group** | 1 | 13 | < 0.05 | 0.837 | 0.829 | < 0.05 | 0.911 | 0.904 | < 0.05 | 0.915 | 0.912 | 0.05 | 0.828 | 0.812 | < 0.05 | 0.884 | 0.883 |
|  | **Material-Type** | 2 | 26 | 0.96 | 0.395 | 0.374 | 0.86 | 0.434 | 0.410 | 0.93 | 0.408 | 0.378 | 1.20 | 0.317 | 0.312 | 1.03 | 0.372 | 0.356 |
|  | **Group*Material-Type** | 2 | 26 | 0.53 | 0.597 | 0.556 | 0.40 | 0.677 | 0.651 | 0.41 | 0.671 | 0.631 | 0.77 | 0.474 | 0.464 | 0.46 | 0.637 | 0.609 |
|  | **Paradigm** | 1 | 13 | 11.60 | 0.005 | 0.006 | 14.50 | 0.002 | 0.004 | 11.89 | 0.004 | 0.005 | 8.10 | 0.014 | 0.017 | 12.29 | 0.004 | 0.004 |
|  | **Group*Paradigm** | 1 | 13 | 0.62 | 0.445 | 0.434 | 0.41 | 0.533 | 0.519 | 0.50 | 0.492 | 0.489 | 0.55 | 0.472 | 0.468 | 0.54 | 0.477 | 0.466 |
|  | **Paradigm*Material-Type** | 2 | 26 | 2.62 | 0.092 | 0.097 | 2.70 | 0.086 | 0.097 | 2.24 | 0.127 | 0.134 | 2.10 | 0.142 | 0.145 | 2.15 | 0.137 | 0.138 |
|  | **Group*Paradigm*Material-Type** | 2 | 26 | 0.43 | 0.655 | 0.645 | 0.39 | 0.682 | 0.640 | 0.24 | 0.785 | 0.755 | 0.27 | 0.765 | 0.758 | 0.22 | 0.803 | 0.785 |
|  | | | | | | | | | | | | | | | | | | |
| **MH vs CTRs**  **Familiarity**  **(3-way ANOVA:**  **Group,**  **Material-Type,**  **Paradigm)** | **Group** | 1 | 13 | 13.74 | 0.003 | 0.003 | 9.99 | 0.008 | 0.005 | 14.31 | 0.002 | 0.002 | 11.06 | 0.005 | 0.005 | 12.16 | 0.004 | 0.005 |
|  | **Material-Type** | 2 | 26 | 6.11 | 0.007 | 0.005 | 6.09 | 0.007 | 0.006 | 4.34 | 0.024 | 0.024 | 4.38 | 0.023 | 0.022 | 4.04 | 0.030 | 0.029 |
|  | **Group*Material-Type** | 2 | 26 | < 0.05 | 0.972 | 0.970 | 0.07 | 0.933 | 0.932 | < 0.05 | 0.959 | 0.959 | < 0.05 | 0.994 | 0.995 | < 0.05 | 0.974 | 0.973 |
|  | **Paradigm** | 1 | 13 | 3.36 | 0.090 | 0.086 | 6.40 | 0.025 | 0.027 | 3.33 | 0.091 | 0.089 | 3.56 | 0.082 | 0.084 | 3.50 | 0.084 | 0.089 |
|  | **Group*Paradigm** | 1 | 13 | 0.09 | 0.765 | 0.759 | < 0.05 | 0.879 | 0.876 | < 0.05 | 0.879 | 0.880 | < 0.05 | 0.967 | 0.966 | 0.17 | 0.687 | 0.699 |
|  | **Paradigm*Material-Type** | 2 | 26 | 4.00 | 0.031 | 0.032 | 4.66 | 0.019 | 0.014 | 4.96 | 0.015 | 0.014 | 5.04 | 0.014 | 0.010 | 5.79 | 0.008 | 0.005 |
|  | **Group*Paradigm*Material-Type** | 2 | 26 | 0.48 | 0.624 | 0.627 | 0.49 | 0.619 | 0.620 | 0.67 | 0.519 | 0.504 | 0.81 | 0.455 | 0.457 | 0.45 | 0.640 | 0.645 |
|  | | | | | | | | | | | | | | | | | | |
| **MH vs CTRs**  **Faces**  **(3-way ANOVA:**  **Group,**  **Process,**  **Paradigm)** | **Group** | 1 | 13 | 5.83 | 0.031 | 0.033 | 3.02 | 0.106 | 0.108 | 3.09 | 0.102 | 0.105 | 5.56 | 0.035 | 0.036 | 5.83 | 0.031 | 0.028 |
|  | **Process** | 1 | 13 | < 0.05 | 0.963 | 0.962 | < 0.05 | 0.995 | 0.995 | < 0.05 | 0.986 | 0.987 | < 0.05 | 0.938 | 0.940 | < 0.05 | 0.941 | 0.945 |
|  | **Group*Process** | 1 | 13 | 1.10 | 0.312 | 0.310 | 1.04 | 0.326 | 0.322 | 1.16 | 0.300 | 0.294 | 1.26 | 0.282 | 0.273 | 1.17 | 0.299 | 0.290 |
|  | **Paradigm** | 1 | 13 | 0.14 | 0.719 | 0.721 | 0.05 | 0.827 | 0.823 | < 0.05 | 0.865 | 0.856 | < 0.05 | 0.956 | 0.956 | 0.07 | 0.797 | 0.789 |
|  | **Group*Paradigm** | 1 | 13 | 0.55 | 0.471 | 0.477 | 0.23 | 0.638 | 0.625 | 0.21 | 0.657 | 0.651 | 0.09 | 0.764 | 0.764 | 0.33 | 0.578 | 0.578 |
|  | **Process*Paradigm** | 1 | 13 | 3.62 | 0.080 | 0.074 | 6.03 | 0.029 | 0.026 | 4.76 | 0.048 | 0.047 | 3.83 | 0.072 | 0.069 | 4.49 | 0.054 | 0.052 |
|  | **Group*Process*Paradigm** | 1 | 13 | 0.09 | 0.772 | 0.776 | 0.06 | 0.809 | 0.799 | 0.12 | 0.738 | 0.731 | 0.09 | 0.763 | 0.755 | 0.16 | 0.695 | 0.704 |
|  | | | | | | | | | | | | | | | | | | |
| **MH vs CTRs**  **Scenes**  **(3-way ANOVA:**  **Group,**  **Process,**  **Paradigm)** | **Group** | 1 | 13 | 5.39 | 0.037 | 0.040 | 4.96 | 0.044 | 0.042 | 4.61 | 0.051 | 0.054 | 6.75 | 0.022 | 0.029 | 3.95 | 0.068 | 0.068 |
|  | **Process** | 1 | 13 | 0.66 | 0.433 | 0.416 | 1.03 | 0.329 | 0.322 | 1.19 | 0.295 | 0.296 | 0.56 | 0.468 | 0.451 | 0.92 | 0.356 | 0.340 |
|  | **Group*Process** | 1 | 13 | 3.26 | 0.094 | 0.095 | 2.60 | 0.131 | 0.131 | 3.75 | 0.075 | 0.079 | 3.09 | 0.102 | 0.108 | 3.50 | 0.084 | 0.080 |
|  | **Paradigm** | 1 | 13 | 0.52 | 0.482 | 0.454 | 0.47 | 0.506 | 0.492 | 0.61 | 0.448 | 0.426 | 0.24 | 0.629 | 0.615 | 0.67 | 0.427 | 0.416 |
|  | **Group*Paradigm** | 1 | 13 | < 0.05 | 0.964 | 0.963 | < 0.05 | 0.978 | 0.977 | < 0.05 | 0.997 | 0.998 | < 0.05 | 0.864 | 0.860 | < 0.05 | 0.954 | 0.949 |
|  | **Process*Paradigm** | 1 | 13 | 28.79 | < 0.001 | < 0.001 | 32.97 | < 0.001 | < 0.001 | 25.16 | < 0.001 | < 0.001 | 39.52 | < 0.001 | < 0.001 | 19.97 | 0.001 | 0.001 |
|  | **Group*Process*Paradigm** | 1 | 13 | 1.05 | 0.325 | 0.313 | 0.51 | 0.487 | 0.465 | 0.62 | 0.446 | 0.433 | 1.42 | 0.255 | 0.246 | 0.56 | 0.469 | 0.459 |
|  | | | | | | | | | | | | | | | | | | |
| **MH vs CTRs**  **Words**  **(3-way ANOVA:**  **Group,**  **Process,**  **Paradigm)** | **Group** | 1 | 13 | 2.02 | 0.179 | 0.181 | 1.98 | 0.183 | 0.182 | 2.23 | 0.159 | 0.151 | 1.15 | 0.303 | 0.306 | 1.61 | 0.227 | 0.239 |
|  | **Process** | 1 | 13 | 6.05 | 0.029 | 0.027 | 3.83 | 0.072 | 0.072 | 5.27 | 0.039 | 0.041 | 4.79 | 0.047 | 0.049 | 4.83 | 0.047 | 0.049 |
|  | **Group*Process** | 1 | 13 | 5.95 | 0.030 | 0.025 | 3.77 | 0.074 | 0.074 | 5.17 | 0.041 | 0.046 | 4.71 | 0.049 | 0.045 | 4.75 | 0.048 | 0.052 |
|  | **Paradigm** | 1 | 13 | 5.32 | 0.038 | 0.039 | 6.72 | 0.022 | 0.019 | 6.78 | 0.022 | 0.021 | 4.42 | 0.056 | 0.055 | 9.02 | 0.010 | 0.006 |
|  | **Group*Paradigm** | 1 | 13 | 2.42 | 0.144 | 0.142 | 3.09 | 0.103 | 0.096 | 3.26 | 0.094 | 0.092 | 2.96 | 0.109 | 0.108 | 2.06 | 0.175 | 0.164 |
|  | **Process*Paradigm** | 1 | 13 | < 0.05 | 0.942 | 0.936 | < 0.05 | 0.992 | 0.993 | < 0.05 | 0.956 | 0.956 | < 0.05 | 0.902 | 0.905 | < 0.05 | 0.921 | 0.924 |
|  | **Group*Process*Paradigm** | 1 | 13 | < 0.05 | 0.841 | 0.840 | <0.05 | 0.881 | 0.880 | < 0.05 | 0.952 | 0.947 | < 0.05 | 0.992 | 0.993 | 0.06 | 0.815 | 0.818 |

ANOVAs (between-participants independent variables: Group (MH (PRC lesion) vs. CTRs); within-participants independent variables: Paradigm (ROC vs. RDP); Process (Familiarity vs. Recollection); Material-Type (Faces vs. Scenes vs. Words)) on (recollection and familiarity) estimates for MH (focal PRC lesion) and CTRs for faces, scenes, and words, in the ROC and the RDP paradigms. Missing values were imputed using “Multiple Imputation with Chained Equations” implemented in the R function “mice”. Five imputations were created (MICE 1-5). shaded cells: significant (p < 0.05) main effects and interactions of interest. ‘p-perm’: p-values calculated from 5000 permutations using the “aovperm” function in R.

## Supplementary table 12: Categorical Analyses: HPC Patients vs. CTRs (including Words)

| **Analysis** | **Main Effect / Interaction** | **df1** | **df2** | **MICE IMPUTATION** | | | | | | | | | | | | | | |
| --- | --- | --- | --- | --- | --- | --- | --- | --- | --- | --- | --- | --- | --- | --- | --- | --- | --- | --- |
|  |  |  |  | **1** | | | **2** | | | **3** | | | **4** | | | **5** | | |
|  |  |  |  | **F** | **p** | **p-perm** | **F** | **p** | **p-perm** | **F** | **p** | **p-perm** | **F** | **p** | **p-perm** | **F** | **p** | **p-perm** |
| **HPC (H1-H7) vs. CTRs**  **(4-way ANOVA)** | **Group** | 1 | 19 | 31.42 | < 0.001 | < 0.001 | 30.45 | < 0.001 | < 0.001 | 27.76 | < 0.001 | < 0.001 | 27.86 | < 0.001 | < 0.001 | 29.52 | < 0.001 | < 0.001 |
|  | **Process** | 1 | 19 | 37.79 | < 0.001 | < 0.001 | 29.85 | < 0.001 | < 0.001 | 38.03 | < 0.001 | < 0.001 | 36.83 | < 0.001 | < 0.001 | 39.97 | < 0.001 | < 0.001 |
|  | **Group*Process** | 1 | 19 | 1.09 | 0.310 | 0.299 | 0.71 | 0.408 | 0.401 | 0.45 | 0.513 | 0.500 | 0.74 | 0.399 | 0.409 | 1.01 | 0.327 | 0.330 |
|  | **Material-Type** | 2 | 38 | 1.60 | 0.215 | 0.220 | 0.65 | 0.530 | 0.542 | 1.05 | 0.359 | 0.352 | 1.03 | 0.367 | 0.363 | 1.20 | 0.313 | 0.320 |
|  | **Group*Material-Type** | 2 | 38 | 9.83 | < 0.001 | < 0.001 | 7.81 | 0.001 | 0.001 | 7.12 | 0.002 | 0.003 | 9.28 | 0.001 | 0.001 | 8.49 | 0.001 | 0.001 |
|  | **Paradigm** | 1 | 19 | 0.56 | 0.464 | 0.472 | 0.64 | 0.434 | 0.437 | 0.45 | 0.509 | 0.507 | < 0.05 | 0.903 | 0.897 | 1.16 | 0.294 | 0.292 |
|  | **Group*Paradigm** | 1 | 19 | 2.84 | 0.109 | 0.108 | 3.04 | 0.097 | 0.095 | 2.67 | 0.119 | 0.124 | 0.75 | 0.398 | 0.390 | 4.34 | 0.051 | 0.057 |
|  | **Material-Type*Process** | 2 | 38 | 10.48 | < 0.001 | < 0.001 | 12.23 | < 0.001 | < 0.001 | 10.08 | < 0.001 | 0.001 | 10.81 | < 0.001 | < 0.001 | 9.86 | < 0.001 | 0.001 |
|  | **Group*Material-Type*Process** | 2 | 38 | 3.02 | 0.061 | 0.055 | 3.64 | 0.036 | 0.035 | 2.40 | 0.105 | 0.099 | 3.76 | 0.032 | 0.034 | 2.49 | 0.096 | 0.094 |
|  | **Paradigm*Process** | 1 | 19 | 57.01 | < 0.001 | < 0.001 | 89.82 | < 0.001 | < 0.001 | 54.35 | < 0.001 | < 0.001 | 62.41 | < 0.001 | < 0.001 | 56.34 | < 0.001 | < 0.001 |
|  | **Group*Paradigm*Process** | 1 | 19 | 1.43 | 0.247 | 0.236 | 0.56 | 0.463 | 0.467 | 0.68 | 0.420 | 0.421 | 0.93 | 0.347 | 0.347 | 1.45 | 0.244 | 0.242 |
|  | **Paradigm*Material-Type** | 2 | 38 | 0.71 | 0.498 | 0.489 | 0.66 | 0.523 | 0.518 | 0.96 | 0.392 | 0.381 | 0.94 | 0.399 | 0.406 | 1.19 | 0.316 | 0.310 |
|  | **Group*Paradigm*Material-Type** | 2 | 38 | 1.10 | 0.344 | 0.355 | 0.51 | 0.604 | 0.612 | 0.58 | 0.563 | 0.564 | 1.04 | 0.364 | 0.365 | 0.92 | 0.406 | 0.401 |
|  | **Paradigm*Material-Type*Process** | 2 | 38 | 13.91 | < 0.001 | < 0.001 | 19.42 | < 0.001 | < 0.001 | 15.64 | < 0.001 | < 0.001 | 13.69 | < 0.001 | < 0.001 | 16.54 | < 0.001 | < 0.001 |
|  | **Group*Paradigm*Material-Type*Process** | 2 | 38 | 0.44 | 0.650 | 0.658 | 0.79 | 0.462 | 0.452 | 0.34 | 0.715 | 0.710 | 0.18 | 0.839 | 0.838 | 0.53 | 0.593 | 0.603 |
|  | | | | | | | | | | | | | | | | | | |
| **HPC (H1-H7) vs. CTRs:**  **Recollection**  **(3-way ANOVA)** | **Group** | 1 | 19 | 7.88 | 0.011 | 0.013 | 8.23 | 0.010 | 0.009 | 9.36 | 0.006 | 0.006 | 8.81 | 0.008 | 0.009 | 9.49 | 0.006 | 0.006 |
|  | **Material-Type** | 2 | 38 | 10.66 | < 0.001 | < 0.001 | 9.38 | < 0.001 | 0.001 | 9.74 | < 0.001 | 0.001 | 12.69 | < 0.001 | < 0.001 | 10.61 | < 0.001 | 0.001 |
|  | **Group*Material-Type** | 2 | 38 | < 0.05 | 0.981 | 0.980 | < 0.05 | 0.976 | 0.979 | 0.11 | 0.899 | 0.904 | 0.13 | 0.881 | 0.886 | < 0.05 | 0.957 | 0.955 |
|  | **Paradigm** | 1 | 19 | 35.48 | < 0.001 | < 0.001 | 44.48 | < 0.001 | < 0.001 | 37.06 | < 0.001 | < 0.001 | 26.19 | < 0.001 | < 0.001 | 37.83 | < 0.001 | < 0.001 |
|  | **Group*Paradigm** | 1 | 19 | < 0.05 | 0.883 | 0.888 | 0.37 | 0.551 | 0.555 | 0.10 | 0.751 | 0.761 | < 0.05 | 0.978 | 0.979 | 0.09 | 0.768 | 0.768 |
|  | **Paradigm*Material-Type** | 2 | 38 | 14.70 | < 0.001 | < 0.001 | 15.45 | < 0.001 | < 0.001 | 13.00 | < 0.001 | < 0.001 | 12.06 | < 0.001 | 0.001 | 12.33 | < 0.001 | < 0.001 |
|  | **Group*Paradigm*Material-Type** | 2 | 38 | 1.13 | 0.334 | 0.339 | 1.08 | 0.352 | 0.353 | 0.57 | 0.568 | 0.559 | 0.62 | 0.541 | 0.532 | 0.47 | 0.631 | 0.635 |
|  | | | | | | | | | | | | | | | | | | |
| **HPC (H1-H7) vs. CTRs:**  **Familiarity**  **(3-way ANOVA)** | **Group** | 1 | 19 | 20.14 | < 0.001 | < 0.001 | 16.49 | 0.001 | 0.001 | 18.18 | < 0.001 | < 0.001 | 16.04 | 0.001 | 0.001 | 18.84 | < 0.001 | 0.001 |
|  | **Material-Type** | 2 | 38 | 5.30 | 0.009 | 0.010 | 6.53 | 0.004 | 0.004 | 4.28 | 0.021 | 0.026 | 4.26 | 0.021 | 0.023 | 4.35 | 0.020 | 0.015 |
|  | **Group*Material-Type** | 2 | 38 | 10.44 | < 0.001 | 0.001 | 12.07 | < 0.001 | < 0.001 | 8.01 | 0.001 | 0.001 | 9.37 | < 0.001 | 0.001 | 8.16 | 0.001 | 0.002 |
|  | **Paradigm** | 1 | 19 | 32.01 | < 0.001 | < 0.001 | 50.51 | < 0.001 | < 0.001 | 31.80 | < 0.001 | < 0.001 | 33.48 | < 0.001 | < 0.001 | 32.87 | < 0.001 | < 0.001 |
|  | **Group*Paradigm** | 1 | 19 | 4.49 | 0.048 | 0.052 | 3.94 | 0.062 | 0.062 | 2.89 | 0.106 | 0.110 | 1.97 | 0.177 | 0.165 | 5.83 | 0.026 | 0.028 |
|  | **Paradigm*Material-Type** | 2 | 38 | 8.24 | 0.001 | 0.001 | 9.66 | < 0.001 | 0.001 | 9.59 | < 0.001 | 0.001 | 9.27 | 0.001 | 0.001 | 12.36 | < 0.001 | 0.001 |
|  | **Group*Paradigm*Material-Type** | 2 | 38 | 0.26 | 0.769 | 0.769 | 0.39 | 0.682 | 0.678 | 0.27 | 0.765 | 0.761 | 0.18 | 0.834 | 0.835 | 0.78 | 0.464 | 0.464 |
|  | | | | | | | | | | | | | | | | | | |
| **HPC (H1-H7) vs. CTRs:**  **Faces**  **(3-way ANOVA)** | **Group** | 1 | 19 | 4.35 | 0.051 | 0.052 | 2.16 | 0.158 | 0.160 | 2.40 | 0.138 | 0.141 | 3.52 | 0.076 | 0.081 | 4.44 | 0.049 | 0.048 |
|  | **Process** | 1 | 19 | 12.48 | 0.002 | 0.002 | 11.97 | 0.003 | 0.002 | 13.11 | 0.002 | 0.002 | 14.13 | 0.001 | 0.001 | 13.05 | 0.002 | 0.002 |
|  | **Group*Process** | 1 | 19 | 0.70 | 0.413 | 0.412 | 0.89 | 0.359 | 0.362 | 0.91 | 0.353 | 0.360 | 1.65 | 0.214 | 0.216 | 0.59 | 0.452 | 0.475 |
|  | **Paradigm** | 1 | 19 | 0.05 | 0.829 | 0.840 | 0.06 | 0.809 | 0.798 | 0.19 | 0.672 | 0.680 | 0.40 | 0.533 | 0.545 | 0.08 | 0.778 | 0.775 |
|  | **Group*Paradigm** | 1 | 19 | 7.98 | 0.011 | 0.009 | 4.07 | 0.058 | 0.061 | 3.96 | 0.061 | 0.061 | 2.41 | 0.137 | 0.135 | 5.41 | 0.031 | 0.031 |
|  | **Process*Paradigm** | 1 | 19 | 18.91 | < 0.001 | 0.001 | 30.36 | < 0.001 | < 0.001 | 24.12 | < 0.001 | 0.001 | 19.90 | < 0.001 | < 0.001 | 22.60 | < 0.001 | < 0.001 |
|  | **Group*Process*Paradigm** | 1 | 19 | 0.05 | 0.827 | 0.832 | < 0.05 | 0.978 | 0.975 | 0.07 | 0.801 | 0.805 | 0.05 | 0.817 | 0.815 | 0.17 | 0.688 | 0.700 |
|  | | | | | | | | | | | | | | | | | | |
| **HPC (H1-H7) vs. CTRs:**  **Scenes**  **(3-way ANOVA)** | **Group** | 1 | 19 | 19.01 | < 0.001 | 0.001 | 18.87 | < 0.001 | < 0.001 | 16.86 | 0.001 | 0.001 | 19.91 | < 0.001 | 0.001 | 16.30 | 0.001 | 0.001 |
|  | **Process** | 1 | 19 | 1.25 | 0.277 | 0.271 | 0.53 | 0.476 | 0.469 | 0.85 | 0.369 | 0.353 | 1.32 | 0.264 | 0.273 | 1.02 | 0.324 | 0.318 |
|  | **Group*Process** | 1 | 19 | 0.70 | 0.412 | 0.418 | 0.20 | 0.660 | 0.658 | 0.38 | 0.543 | 0.546 | 0.77 | 0.392 | 0.382 | 0.52 | 0.480 | 0.480 |
|  | **Paradigm** | 1 | 19 | 0.95 | 0.341 | 0.346 | 1.06 | 0.317 | 0.323 | 1.17 | 0.292 | 0.293 | 0.33 | 0.571 | 0.560 | 1.10 | 0.308 | 0.313 |
|  | **Group*Paradigm** | 1 | 19 | 0.27 | 0.612 | 0.606 | 0.38 | 0.546 | 0.544 | 0.38 | 0.545 | 0.555 | < 0.05 | 0.876 | 0.873 | 0.30 | 0.591 | 0.600 |
|  | **Process*Paradigm** | 1 | 19 | 87.38 | < 0.001 | < 0.001 | 100.91 | < 0.001 | < 0.001 | 83.52 | < 0.001 | < 0.001 | 103.18 | < 0.001 | < 0.001 | 71.53 | < 0.001 | < 0.001 |
|  | **Group*Process*Paradigm** | 1 | 19 | 0.33 | 0.575 | 0.573 | 0.00 | 0.946 | 0.942 | 0.06 | 0.816 | 0.814 | 0.37 | 0.552 | 0.552 | 0.10 | 0.760 | 0.751 |
|  | | | | | | | | | | | | | | | | | | |
| **HPC (H1-H7) vs. CTRs:**  **Words**  **(3-way ANOVA)** | **Group** | 1 | 19 | 44.46 | < 0.001 | < 0.001 | 41.97 | < 0.001 | < 0.001 | 47.13 | < 0.001 | < 0.001 | 31.13 | < 0.001 | < 0.001 | 37.81 | < 0.001 | < 0.001 |
|  | **Process** | 1 | 19 | 54.93 | < 0.001 | < 0.001 | 41.68 | < 0.001 | < 0.001 | 49.64 | < 0.001 | < 0.001 | 47.84 | < 0.001 | < 0.001 | 48.09 | < 0.001 | < 0.001 |
|  | **Group*Process** | 1 | 19 | 5.73 | 0.027 | 0.028 | 4.99 | 0.038 | 0.044 | 3.60 | 0.073 | 0.070 | 4.62 | 0.045 | 0.042 | 4.70 | 0.043 | 0.041 |
|  | **Paradigm** | 1 | 19 | 0.51 | 0.482 | 0.489 | 0.59 | 0.453 | 0.453 | 0.52 | 0.481 | 0.487 | 0.09 | 0.770 | 0.762 | 2.43 | 0.136 | 0.127 |
|  | **Group*Paradigm** | 1 | 19 | 1.00 | 0.331 | 0.325 | 1.15 | 0.298 | 0.303 | 1.05 | 0.318 | 0.313 | 0.33 | 0.572 | 0.570 | 3.48 | 0.078 | 0.077 |
|  | **Process*Paradigm** | 1 | 19 | 0.80 | 0.383 | 0.380 | 1.75 | 0.202 | 0.202 | 1.31 | 0.267 | 0.274 | 2.04 | 0.170 | 0.170 | 0.80 | 0.383 | 0.396 |
|  | **Group*Process*Paradigm** | 1 | 19 | 2.53 | 0.128 | 0.132 | 2.77 | 0.113 | 0.113 | 1.33 | 0.262 | 0.262 | 1.30 | 0.269 | 0.279 | 3.01 | 0.099 | 0.101 |
|  | | | | | | | | | | | | | | | | | | |
| **HPC (H1-H7) vs. CTRs:**  **Familiarity: Faces**  **(2-way ANOVA)** | **Group** | 1 | 19 | 0.26 | 0.616 | 0.619 | 0.07 | 0.795 | 0.797 | 0.10 | 0.751 | 0.754 | < 0.05 | 0.976 | 0.975 | 0.35 | 0.560 | 0.559 |
|  | **Paradigm** | 1 | 19 | 20.51 | < 0.001 | < 0.001 | 26.23 | < 0.001 | < 0.001 | 29.06 | < 0.001 | < 0.001 | 24.71 | < 0.001 | < 0.001 | 21.50 | < 0.001 | < 0.001 |
|  | **Group*Paradigm** | 1 | 19 | 2.27 | 0.148 | 0.157 | 1.64 | 0.216 | 0.212 | 2.48 | 0.132 | 0.119 | 1.56 | 0.227 | 0.226 | 2.78 | 0.112 | 0.113 |
|  | | | | | | | | | | | | | | | | | | |
| **HPC (H1-H7) vs. CTRs:**  **Familiarity: Scenes**  **(2-way ANOVA)** | **Group** | 1 | 19 | 12.58 | 0.002 | 0.002 | 12.34 | 0.002 | 0.003 | 12.30 | 0.002 | 0.003 | 13.45 | 0.002 | 0.002 | 11.08 | 0.004 | 0.003 |
|  | **Paradigm** | 1 | 19 | 36.47 | < 0.001 | < 0.001 | 38.30 | < 0.001 | < 0.001 | 32.74 | < 0.001 | < 0.001 | 37.41 | < 0.001 | < 0.001 | 42.73 | < 0.001 | < 0.001 |
|  | **Group*Paradigm** | 1 | 19 | 0.58 | 0.457 | 0.451 | 0.16 | 0.690 | 0.693 | 0.30 | 0.591 | 0.596 | 0.25 | 0.620 | 0.626 | 0.39 | 0.540 | 0.555 |
|  | | | | | | | | | | | | | | | | | | |
| **HPC (H1-H7) vs. CTRs:**  **Familiarity: Words**  **(2-way ANOVA)** | **Group** | 1 | 19 | 24.18 | < 0.001 | < 0.001 | 19.48 | < 0.001 | 0.001 | 19.95 | < 0.001 | < 0.001 | 16.57 | 0.001 | 0.001 | 19.54 | < 0.001 | < 0.001 |
|  | **Paradigm** | 1 | 19 | 0.06 | 0.812 | 0.809 | 0.23 | 0.641 | 0.656 | 0.28 | 0.601 | 0.600 | 0.73 | 0.403 | 0.398 | < 0.05 | 0.886 | 0.887 |
|  | **Group*Paradigm** | 1 | 19 | 2.33 | 0.144 | 0.142 | 2.94 | 0.103 | 0.096 | 1.86 | 0.189 | 0.198 | 1.26 | 0.275 | 0.273 | 4.78 | 0.042 | 0.043 |

ANOVAs (between-participants independent variables: Group (HPC lesion (H1-H7), CTRs); within-participants independent variables: Paradigm (ROC vs. RDP); Process (Familiarity vs. Recollection); Material-Type (Faces vs. Scenes vs. Words)) on (recollection and familiarity) estimates for MH (focal PRC lesion) and CTRs for faces, scenes, and words, in the ROC and the RDP paradigms. These missing values were imputed using “Multiple Imputation with Chained Equations” implemented in the R function “mice”. Five imputations were created (MICE 1-5). shaded cells: significant (p < 0.05) main effects and interactions of interest. ‘p-perm’: p-values calculated from 5000 permutations.

## Supplementary table 13: Categorical Analyses: MH vs HPC patients (including Words)

| **Linear Model – Fixed Effects** | **Effects/Interactions** | **df1** | **df2** | **F** | **p** | **p-perm** |
| --- | --- | --- | --- | --- | --- | --- |
| Group, Paradigm, Process, Material-Type | Group | 1 | 6 | < 0.05 | 0.995 | 0.997 |
|  | Process | 1 | 6 | 9.24 | 0.023 | 0.025 |
|  | Group*Process | 1 | 6 | 6.90 | 0.039 | 0.042 |
|  | Material-Type | 2 | 12 | 1.32 | 0.303 | 0.305 |
|  | Group*Material-Type | 2 | 12 | 4.48 | 0.035 | 0.040 |
|  | Paradigm | 1 | 6 | 0.33 | 0.588 | 0.595 |
|  | Group*Paradigm | 1 | 6 | 0.74 | 0.422 | 0.425 |
|  | Process*Material-Type | 2 | 12 | 0.53 | 0.602 | 0.590 |
|  | Group*Process*Material-Type | 2 | 12 | 0.05 | 0.951 | 0.949 |
|  | Process*Paradigm | 1 | 6 | 3.55 | 0.108 | 0.106 |
|  | Group*Process*Paradigm | 1 | 6 | < 0.05 | > 0.999 | > 0.999 |
|  | Material-Type*Paradigm | 2 | 12 | 0.91 | 0.427 | 0.424 |
|  | Group*Material-Type*Paradigm | 2 | 12 | 1.56 | 0.249 | 0.258 |
|  | Process*Material-Type*Paradigm | 2 | 12 | 0.28 | 0.758 | 0.752 |
|  | Group*Process*Material-Type*Paradigm | 2 | 12 | 0.38 | 0.692 | 0.687 |
|  | | | | | | |
| Group (Recollection) | Group | 1 | 6 | 3.62 | 0.106 | 0.249 |
| Group (Familiarity) | Group | 1 | 6 | 1.84 | 0.224 | 0.251 |
|  | | | | | | |
| Group (Faces) | Group | 1 | 6 | 5.63 | 0.055 | 0.130 |
| Group (Scenes) | Group | 1 | 6 | 0.05 | 0.838 | 0.883 |
| Group (Words) | Group | 1 | 6 | 3.60 | 0.107 | 0.129 |

We added both MH and the 7 HPC cases (H1-7) to a single linear model, which was fit to patients’ Z-scores relative to CTRs. The model included fixed effects of: Group (PRC vs. HPC lesion); Paradigm (ROC vs. RDP); Process (Recollection vs Familiarity) and Material-Type (Faces vs. Scenes vs. Words); shaded cells: significant (p < 0.05) main effects and interactions of interest. ‘p-perm’: p-values calculated from 5000 permutations using the “aovperm” function in R.

## Supplementary table 14: Continuous Analyses : Separate models for HPC / ERC / PRC / PHC (including words)

| **Model** | **Effect/Interaction** | **Df1** | **Df2** | **F** | **p** |
| --- | --- | --- | --- | --- | --- |
| ROI: HPC  **Predictors:**  ROI volume  Process (Recollection vs. Familiarity)  Material-Type (Faces vs. Scenes vs. Words)  Paradigm (ROC vs. RDP);  **Outcome variable:**  (Recollection and Familiarity) estimates | Volume | 1 | 7 | 1.80 | 0.222 |
|  | Material-Type | 2 | 77 | 0.37 | 0.689 |
|  | Paradigm | 1 | 77 | 2.65 | 0.108 |
|  | Process | 1 | 77 | 13.96 | < 0.001 |
|  | Volume*Material-Type | 2 | 77 | 1.41 | 0.251 |
|  | Volume*Paradigm | 1 | 77 | 3.18 | 0.078 |
|  | Material-Type*Paradigm | 2 | 77 | 0.93 | 0.400 |
|  | Volume*Process | 1 | 77 | 10.03 | 0.002 |
|  | Material-Type*Process | 2 | 77 | 0.15 | 0.857 |
|  | Paradigm*Process | 1 | 77 | 0.95 | 0.334 |
|  | Volume*Material-Type*Paradigm | 2 | 77 | 0.85 | 0.433 |
|  | Volume*Material-Type*Process | 2 | 77 | 0.12 | 0.883 |
|  | Volume*Paradigm*Process | 1 | 77 | 0.16 | 0.694 |
|  | Material-Type*Paradigm*Process | 2 | 77 | < 0.05 | 0.982 |
|  | Volume*Material-Type*Paradigm*Process | 2 | 77 | 0.05 | 0.955 |
| ROI: PRC  **Predictors:**  ROI volume  Process (Recollection vs. Familiarity)  Material-Type (Faces vs. Scenes vs. Words)  Paradigm (ROC vs. RDP);  **Outcome variable:**  (Recollection and Familiarity) estimates | Volume | 1 | 7 | 0.31 | 0.593 |
|  | Material-Type | 2 | 77 | 8.37 | 0.001 |
|  | Paradigm | 1 | 77 | 0.49 | 0.485 |
|  | Process | 1 | 77 | 0.67 | 0.415 |
|  | Volume*Material-Type | 2 | 77 | 1.70 | 0.190 |
|  | Volume*Paradigm | 1 | 77 | 0.88 | 0.352 |
|  | Material-Type*Paradigm | 2 | 77 | 0.52 | 0.598 |
|  | Volume*Process | 1 | 77 | 0.53 | 0.471 |
|  | Material-Type*Process | 2 | 77 | 1.42 | 0.247 |
|  | Paradigm*Process | 1 | 77 | 7.47 | 0.008 |
|  | Volume*Material-Type*Paradigm | 2 | 77 | 0.20 | 0.822 |
|  | Volume*Material-Type*Process | 2 | 77 | 0.57 | 0.567 |
|  | Volume*Paradigm*Process | 1 | 77 | 0.59 | 0.443 |
|  | Material-Type*Paradigm*Process | 2 | 77 | 1.62 | 0.204 |
|  | Volume*Material-Type*Paradigm*Process | 2 | 77 | 1.33 | 0.271 |
| ROI: ERC  **Predictors:**  ROI volume  Process (Recollection vs. Familiarity)  Material-Type (Faces vs. Scenes vs. Words)  Paradigm (ROC vs. RDP);  **Outcome variable:**  (Recollection and Familiarity) estimates | Volume | 1 | 7 | 1.71 | 0.232 |
|  | Material-Type | 2 | 77 | 1.24 | 0.296 |
|  | Paradigm | 1 | 77 | 0.74 | 0.392 |
|  | Process | 1 | 77 | 1.02 | 0.315 |
|  | Volume*Material-Type | 2 | 77 | 1.13 | 0.327 |
|  | Volume*Paradigm | 1 | 77 | 1.21 | 0.276 |
|  | Material-Type*Paradigm | 2 | 77 | 0.05 | 0.949 |
|  | Volume*Process | 1 | 77 | 0.08 | 0.774 |
|  | Material-Type*Process | 2 | 77 | 0.43 | 0.651 |
|  | Paradigm*Process | 1 | 77 | 2.93 | 0.091 |
|  | Volume*Material-Type*Paradigm | 2 | 77 | 0.38 | 0.684 |
|  | Volume*Material-Type*Process | 2 | 77 | 0.24 | 0.785 |
|  | Volume*Paradigm*Process | 1 | 77 | 0.12 | 0.733 |
|  | Material-Type*Paradigm*Process | 2 | 77 | 0.44 | 0.646 |
|  | Volume*Material-Type*Paradigm*Process | 2 | 77 | 0.07 | 0.935 |
| ROI: PHC  **Predictors:**  ROI volume  Process (Recollection vs. Familiarity)  Material-Type (Faces vs. Scenes vs. Words)  Paradigm (ROC vs. RDP);  **Outcome variable:**  (Recollection and Familiarity) estimates | Volume | 1 | 7 | 6.20 | 0.042 |
|  | Material-Type | 2 | 77 | 5.14 | 0.008 |
|  | Paradigm | 1 | 77 | 0.82 | 0.369 |
|  | Process | 1 | 77 | 1.25 | 0.266 |
|  | Volume*Material-Type | 2 | 77 | 0.85 | 0.433 |
|  | Volume*Paradigm | 1 | 77 | 1.36 | 0.248 |
|  | Material-Type*Paradigm | 2 | 77 | 0.21 | 0.812 |
|  | Volume*Process | 1 | 77 | 10.62 | 0.002 |
|  | Material-Type*Process | 2 | 77 | 0.16 | 0.853 |
|  | Paradigm*Process | 1 | 77 | 1.90 | 0.172 |
|  | Volume*Material-Type*Paradigm | 2 | 77 | < 0.05 | 0.994 |
|  | Volume*Material-Type*Process | 2 | 77 | 0.95 | 0.392 |
|  | Volume*Paradigm*Process | 1 | 77 | 1.13 | 0.291 |
|  | Material-Type*Paradigm*Process | 2 | 77 | 0.92 | 0.402 |
|  | Volume*Material-Type*Paradigm*Process | 2 | 77 | 1.86 | 0.162 |

Results from fitting a series of linear mixed-effects models, for recollection and familiarity estimates, across all patients (n=9). One model was fit for each ROI: HPC / ERC / PRC / PHC. Each model involved the following predictors: ROI volume (averaged across hemispheres); Paradigm (ROC vs. RDP); Process (Recollection vs. Familiarity); Material Type (Faces vs. Scenes vs. Words). **Key: ROI:** region of interest; **PHC:** parahippocampal cortex; **PRC:** perirhinal cortex; **HPC:** hippocampus; **ERC:** entorhinal cortex; shaded cells: significant (p < 0.05) main effects and interactions of interest.

## Supplementary table 15a: Continuous Analyses : Separate models for HPC / PHC x Recollection / Familiarity (including Words)

| **Model** | **Effect/Interaction** | **Df1** | **Df2** | **F** | **p** |
| --- | --- | --- | --- | --- | --- |
| ROI: HPC  **Predictors:**  ROI volume  Material-Type (Faces vs. Scenes vs. Words)  Paradigm (ROC vs. RDP)  **Outcome variable:**  Recollection estimates | Volume | 1 | 42 | 19.96 | < 0.001 |
|  | Material-Type | 2 | 42 | 0.41 | 0.665 |
|  | Paradigm | 1 | 42 | 4.31 | 0.044 |
|  | Volume *Material-Type | 2 | 42 | 0.48 | 0.622 |
|  | Volume *Paradigm | 1 | 42 | 1.23 | 0.273 |
|  | Material-Type*Paradigm | 2 | 42 | 0.77 | 0.472 |
|  | Volume *Material-Type*Paradigm | 2 | 42 | 0.33 | 0.724 |
| ROI: HPC  **Predictors:**  ROI volume  Material-Type (Faces vs. Scenes vs. Words)  Paradigm (ROC vs. RDP)  **Outcome variable:**  Familiarity Estimates | Volume | 1 | 7 | 0.07 | 0.799 |
|  | Material-Type | 2 | 35 | 0.18 | 0.834 |
|  | Paradigm | 1 | 35 | 0.19 | 0.666 |
|  | Volume *Material-Type | 2 | 35 | 1.02 | 0.370 |
|  | Volume *Paradigm | 1 | 35 | 2.10 | 0.156 |
|  | Material-Type*Paradigm | 2 | 35 | 0.31 | 0.738 |
|  | Volume *Material-Type*Paradigm | 2 | 35 | 0.56 | 0.574 |
| ROI: PHC  **Predictors:**  ROI volume  Material-Type (Faces vs. Scenes vs. Words)  Paradigm (ROC vs. RDP)  **Outcome variable:**  Recollection Estimates | Volume | 1 | 7 | < 0.05 | 0.848 |
|  | Material-Type | 2 | 35 | 2.41 | 0.104 |
|  | Paradigm | 1 | 35 | 0.15 | 0.698 |
|  | Volume *Material-Type | 2 | 35 | 1.96 | 0.156 |
|  | Volume *Paradigm | 1 | 35 | 3.36 | 0.075 |
|  | Material-Type*Paradigm | 2 | 35 | 0.24 | 0.786 |
|  | Volume *Material-Type*Paradigm | 2 | 35 | 1.12 | 0.337 |
| ROI: PHC  **Predictors:**  ROI volume  Material-Type (Faces vs. Scenes vs. Words)  Paradigm (ROC vs. RDP)  **Outcome variable:**  Familiarity Estimates | Volume | 1 | 42 | 21.40 | < 0.001 |
|  | Material-Type | 2 | 42 | 3.09 | 0.056 |
|  | Paradigm | 1 | 42 | 2.29 | 0.138 |
|  | Volume *Material-Type | 2 | 42 | 0.30 | 0.740 |
|  | Volume *Paradigm | 1 | 42 | < 0.05 | 0.947 |
|  | Material-Type*Paradigm | 2 | 42 | 0.84 | 0.440 |
|  | Volume*Material-Type*Paradigm | 2 | 42 | 0.91 | 0.410 |

Results from fitting a series of linear mixed-effects models, across all patients (n=9). One model was fit for each of the two ROIs of interest (HPC, PHC), separately for Recollection and Familiarity estimates. Each model involved the following predictors: ROI volume (averaged across hemispheres); Paradigm (ROC vs. RDP); Material Type (Faces vs. Scenes vs. Words). **Key: ROI:** region of interest; **PHC:** parahippocampal cortex; **HPC:** hippocampus; shaded cells: significant (p < 0.05) main effects and interactions of interest.

## Supplementary table 15b: Continuous Analyses : Separate models for HPC / PHC x Recollection / Familiarity (+ Age) (including Words)

| **Model** | **Effect/Interaction** | **Df1** | **Df2** | **F** | **p** |
| --- | --- | --- | --- | --- | --- |
| ROI: HPC  **Predictors:**  ROI volume  Material-Type (Faces vs. Scenes vs. Words)  Paradigm (ROC vs. RDP)  Age  **Outcome variable:**  Recollection estimates | Volume | 1 | 41 | 14.26 | 0.001 |
|  | Material-Type | 2 | 41 | 0.40 | 0.670 |
|  | Paradigm | 1 | 41 | 4.23 | 0.046 |
|  | Age | 1 | 41 | 0.27 | 0.609 |
|  | Volume*Material-Type | 2 | 41 | 0.47 | 0.627 |
|  | Volume*Paradigm | 1 | 41 | 1.21 | 0.278 |
|  | Material-Type*Paradigm | 2 | 41 | 0.75 | 0.478 |
|  | Volume*Material-Type*Paradigm | 2 | 41 | 0.32 | 0.728 |
| ROI: HPC  **Predictors:**  ROI volume  Material-Type (Faces vs. Scenes vs. Words)  Paradigm (ROC vs. RDP)  Age  **Outcome variable:**  Familiarity Estimates | Volume | 1 | 6 | 0.54 | 0.492 |
|  | Material-Type | 2 | 35 | 0.18 | 0.834 |
|  | Paradigm | 1 | 35 | 0.19 | 0.666 |
|  | Age | 1 | 6 | 1.29 | 0.300 |
|  | Volume*Material-Type | 2 | 35 | 1.02 | 0.370 |
|  | Volume*Paradigm | 1 | 35 | 2.10 | 0.156 |
|  | Material-Type*Paradigm | 2 | 35 | 0.31 | 0.738 |
|  | Volume*Material-Type*Paradigm | 2 | 35 | 0.56 | 0.574 |
| ROI: PHC  **Predictors:**  ROI volume  Material-Type (Faces vs. Scenes vs. Words)  Paradigm (ROC vs. RDP)  Age  **Outcome variable:**  Recollection Estimates | Volume | 1 | 6 | < 0.05 | 0.843 |
|  | Material-Type | 2 | 35 | 2.41 | 0.104 |
|  | Paradigm | 1 | 35 | 0.15 | 0.698 |
|  | Age | 1 | 6 | 1.91 | 0.216 |
|  | Volume*Material-Type | 2 | 35 | 1.96 | 0.156 |
|  | Volume*Paradigm | 1 | 35 | 3.36 | 0.075 |
|  | Material-Type*Paradigm | 2 | 35 | 0.24 | 0.786 |
|  | Volume*Material-Type*Paradigm | 2 | 35 | 1.12 | 0.337 |
| ROI: PHC  **Predictors:**  ROI volume  Material-Type (Faces vs. Scenes vs. Words)  Paradigm (ROC vs. RDP)  Age  **Outcome variable:**  Familiarity Estimates | Volume | 1 | 41 | 18.31 | < 0.001 |
|  | Material-Type | 2 | 41 | 3.02 | 0.060 |
|  | Paradigm | 1 | 41 | 2.24 | 0.142 |
|  | Age | 1 | 41 | 0.10 | 0.756 |
|  | Volume*Material-Type | 2 | 41 | 0.30 | 0.745 |
|  | Volume*Paradigm | 1 | 41 | < 0.05 | 0.948 |
|  | Material-Type*Paradigm | 2 | 41 | 0.82 | 0.448 |
|  | Volume*Material-Type*Paradigm | 2 | 41 | 0.89 | 0.418 |

Results from fitting a series of linear mixed-effects models, across all patients (n=9). One model was fit for each of the two ROIs of interest (HPC, PHC), separately for Recollection and Familiarity estimates. Each model involved the following predictors: ROI volume (averaged across hemispheres); Paradigm (ROC vs. RDP); Material Type (Faces vs. Scenes vs. Words), and Age. **Key: ROI:** region of interest; **PHC:** parahippocampal cortex; **HPC:** hippocampus; shaded cells: significant (p < 0.05) main effects and interactions of interest.

## Supplementary table 16: Continuous Analyses: Linear regression of Average HPC / PRC / ERC / PHC volume against Average Recollection and Familiarity estimates (including Words)

| **Average**  **volume**  **(z)** | **Estimate (z)** | | | | | |
| --- | --- | --- | --- | --- | --- | --- |
|  | **Recollection** | | | **Familiarity** | | |
|  | **R^2^** | **p** | **p-perm** | **R^2^** | **p** | **p-perm** |
| **HPC** | 0.84 | < 0.001 | < 0.001 | < 0.05 | 0.799 | 0.810 |
| **PRC** | < 0.05 | 0.883 | 0.876 | 0.06 | 0.514 | 0.502 |
| **ERC** | 0.11 | 0.373 | 0.359 | 0.13 | 0.340 | 0.331 |
| **PHC** | < 0.05 | 0.848 | 0.846 | 0.86 | < 0.001 | < 0.001 |

Double dissociation in brain-behavior relationships between HPC volume – Recollection and PHC volume – Familiarity across patients; **key: ERC:** entorhinal cortex; **HPC:** hippocampus; **PHC:** parahippocampal cortex; **PRC:** perirhinal cortex; **Z:** volumes are expressed as Z-scores, based on the mean and standard deviation of the volumes of the 48 CTRs whose MTL structures were manually delineated (see Argyropoulos et al. (2019) for details); Familiarity and Recollection estimates are expressed as Z-scores, based on the mean and standard deviation of the CTRs that completed the two tasks; p-perm: p-values were calculated from 5000 permutations using the “lmperm” function in R; shaded cells: significant (p < 0.05) main effects and interactions of interest.

## Supplementary table 17: Continuous Analyses: HPC and PHC volumes in a single model (including Words)

| **Model** | **Effect/Interaction** | **Df1** | **Df2** | **F** | **p** |
| --- | --- | --- | --- | --- | --- |
| **‘ROI model’**  **Predictors:**  ROI average volume (HPC vs. PHC)  Process (Recollection vs. Familiarity)  Material-Type (Faces vs. Scenes vs. Words)  Paradigm (ROC vs. RDP);  **Outcome variable:** (Recollection and Familiarity) estimates | HPC | 1 | 60 | 1.56 | 0.216 |
|  | PHC | 1 | 60 | 1.48 | 0.229 |
|  | Process | 1 | 60 | 0.69 | 0.409 |
|  | Material-Type | 2 | 60 | 1.41 | 0.253 |
|  | Paradigm | 1 | 60 | 2.83 | 0.098 |
|  | HPC*PHC | 1 | 60 | < 0.05 | 0.933 |
|  | HPC*Process | 1 | 60 | 1.21 | 0.276 |
|  | PHC*Process | 1 | 60 | 0.39 | 0.534 |
|  | HPC*Material-Type | 2 | 60 | 0.60 | 0.552 |
|  | PHC*Material-Type | 2 | 60 | 1.83 | 0.169 |
|  | Process*Material-Type | 2 | 60 | 2.14 | 0.127 |
|  | HPC*Paradigm | 1 | 60 | 3.70 | 0.059 |
|  | PHC*Paradigm | 1 | 60 | 1.28 | 0.263 |
|  | Process*Paradigm | 1 | 60 | 2.28 | 0.137 |
|  | Material-Type*Paradigm | 2 | 60 | 1.34 | 0.271 |
|  | HPC*PHC*Process | 1 | 60 | < 0.05 | 0.892 |
|  | HPC*PHC*Material-Type | 2 | 60 | 1.51 | 0.229 |
|  | HPC*Process*Material-Type | 2 | 60 | 2.23 | 0.116 |
|  | PHC*Process*Material-Type | 2 | 60 | 2.36 | 0.103 |
|  | HPC*PHC*Paradigm | 1 | 60 | 1.98 | 0.164 |
|  | HPC*Process*Paradigm | 1 | 60 | 3.45 | 0.068 |
|  | PHC*Process*Paradigm | 1 | 60 | 3.99 | 0.050 |
|  | HPC*Material-Type*Paradigm | 2 | 60 | 1.17 | 0.317 |
|  | PHC*Material-Type*Paradigm | 2 | 60 | 0.92 | 0.403 |
|  | Process*Material-Type*Paradigm | 2 | 60 | 1.04 | 0.360 |
|  | HPC*PHC*Process*Material-Type | 2 | 60 | 2.36 | 0.103 |
|  | HPC*PHC*Process*Paradigm | 1 | 60 | 2.94 | 0.092 |
|  | HPC*PHC*Material-Type*Paradigm | 2 | 60 | 0.90 | 0.412 |
|  | HPC*Process*Material-Type*Paradigm | 2 | 60 | 0.72 | 0.491 |
|  | PHC*Process*Material-Type*Paradigm | 2 | 60 | 1.31 | 0.277 |
|  | HPC*PHC*Process*Material-Type*Paradigm | 2 | 60 | 0.58 | 0.563 |

Results from a linear mixed-effects model, where the volumes of the 2 ROIs of interest (HPC, PHC), averaged across hemispheres, are entered as predictors. **Key: ROI:** region of interest; **PHC:** parahippocampal cortex; **HPC:** hippocampus; shaded cells: significant (p < 0.05) main effects and interactions of interest.

## Supplementary table 18: Continuous Analyses: ROI vs MTL model (including Words)

We compared a combined model with HPC, PHC, ERC, and PRC volumes (‘ROI model’) with a single model (‘MTL model’) that includes only total MTL volume (sum of the four ROIs). The model fit index of AIC prefers the ROI model (AIC=180.13) over the MTL model (AIC=222.56; χ^2^ = 106.43, p < 0.001), while the two models have similar BICs (ROI model: BIC = 293.56; MTL model: BIC = 263.54).

| **Model** | **Effect/Interaction** | **Df1** | **Df2** | **F** | **p** |
| --- | --- | --- | --- | --- | --- |
| **‘ROI model’**  **Predictors:**  ROI average volume (HPC vs. PHC vs. ERC vs. PRC)  Process (Recollection vs. Familiarity)  Material-Type (Faces vs. Scenes vs. Words)  Paradigm (ROC vs. RDP);  **Outcome variable:** (Recollection and Familiarity) estimates | HPC | 1 | 3 | 1.30 | 0.337 |
|  | PHC | 1 | 3 | 0.99 | 0.394 |
|  | ERC | 1 | 3 | < 0.05 | 0.990 |
|  | PRC | 1 | 3 | 1.63 | 0.292 |
|  | Process | 1 | 33 | 0.05 | 0.824 |
|  | Material-Type | 2 | 33 | 0.44 | 0.651 |
|  | Paradigm | 1 | 33 | 0.46 | 0.501 |
|  | HPC*PHC | 1 | 3 | < 0.05 | 0.959 |
|  | HPC*Process | 1 | 33 | 0.35 | 0.560 |
|  | PHC*Process | 1 | 33 | 0.86 | 0.361 |
|  | ERC*Process | 1 | 33 | 0.44 | 0.514 |
|  | PRC*Process | 1 | 33 | < 0.05 | 0.943 |
|  | HPC*Material-Type | 2 | 33 | 0.34 | 0.716 |
|  | PHC*Material-Type | 2 | 33 | 0.77 | 0.469 |
|  | ERC*Material-Type | 2 | 33 | 1.03 | 0.368 |
|  | PRC*Material-Type | 2 | 33 | 3.00 | 0.063 |
|  | Process*Material-Type | 2 | 33 | 5.44 | 0.009 |
|  | HPC*Paradigm | 1 | 33 | 1.31 | 0.261 |
|  | PHC*Paradigm | 1 | 33 | 0.13 | 0.723 |
|  | ERC*Paradigm | 1 | 33 | 0.88 | 0.355 |
|  | PRC*Paradigm | 1 | 33 | 0.19 | 0.664 |
|  | Process*Paradigm | 1 | 33 | 5.42 | 0.026 |
|  | Material-Type*Paradigm | 2 | 33 | 0.41 | 0.669 |
|  | HPC*PHC*Process | 1 | 33 | 0.12 | 0.726 |
|  | HPC*PHC*Material-Type | 2 | 33 | 0.44 | 0.645 |
|  | HPC*Process*Material-Type | 2 | 33 | 5.81 | 0.007 |
|  | PHC*Process*Material-Type | 2 | 33 | 6.43 | 0.004 |
|  | ERC*Process*Material-Type | 2 | 33 | 4.09 | 0.026 |
|  | PRC*Process*Material-Type | 2 | 33 | 2.59 | 0.090 |
|  | HPC*PHC*Paradigm | 1 | 33 | 0.18 | 0.672 |
|  | HPC*Process*Paradigm | 1 | 33 | 7.27 | 0.011 |
|  | PHC*Process*Paradigm | 1 | 33 | 8.33 | 0.007 |
|  | ERC*Process*Paradigm | 1 | 33 | 3.85 | 0.058 |
|  | PRC*Process*Paradigm | 1 | 33 | 2.63 | 0.115 |
|  | HPC*Material-Type*Paradigm | 2 | 33 | 0.39 | 0.678 |
|  | PHC*Material-Type*Paradigm | 2 | 33 | 0.16 | 0.857 |
|  | ERC*Material-Type*Paradigm | 2 | 33 | 0.40 | 0.673 |
|  | PRC*Material-Type*Paradigm | 2 | 33 | 0.60 | 0.555 |
|  | Process*Material-Type*Paradigm | 2 | 33 | 0.91 | 0.414 |
|  | HPC*PHC*Process*Material-Type | 2 | 33 | 6.61 | 0.004 |
|  | HPC*PHC*Process*Paradigm | 1 | 33 | 7.13 | 0.012 |
|  | HPC*PHC*Material-Type*Paradigm | 2 | 33 | 0.09 | 0.918 |
|  | HPC*Process*Material-Type*Paradigm | 2 | 33 | 0.85 | 0.437 |
|  | PHC*Process*Material-Type*Paradigm | 2 | 33 | 1.45 | 0.250 |
|  | ERC*Process*Material-Type*Paradigm | 2 | 33 | 0.29 | 0.754 |
|  | PRC*Process*Material-Type*Paradigm | 2 | 33 | 1.94 | 0.160 |
|  | HPC*PHC*Process*Material-Type*Paradigm | 2 | 33 | 0.77 | 0.470 |
| **‘MTL model’**  **Predictors:**  MTL volume (HPC + ERC + PRC + PHC volume)  Process (Recollection vs. Familiarity)  Material-Type (Faces vs. Scenes vs. Words)  Paradigm (ROC vs. RDP);  **Outcome variable:** (Recollection and Familiarity) estimates | MTL | 1 | 7 | 9.56 | 0.018 |
|  | Process | 1 | 77 | 1.09 | 0.300 |
|  | Material-Type | 2 | 77 | 0.38 | 0.688 |
|  | Paradigm | 1 | 77 | 0.05 | 0.822 |
|  | MTL*Process | 1 | 77 | 0.16 | 0.694 |
|  | MTL*Material-Type | 2 | 77 | 0.41 | 0.666 |
|  | Process*Material-Type | 2 | 77 | 0.03 | 0.975 |
|  | MTL*Paradigm | 1 | 77 | 0.05 | 0.816 |
|  | Process*Paradigm | 1 | 77 | 0.48 | 0.490 |
|  | Material-Type*Paradigm | 2 | 77 | 0.37 | 0.695 |
|  | MTL*Process*Material-Type | 2 | 77 | 0.52 | 0.595 |
|  | MTL*Process*Paradigm | 1 | 77 | 0.17 | 0.681 |
|  | MTL*Material-Type*Paradigm | 2 | 77 | 0.34 | 0.713 |
|  | Process*Material-Type*Paradigm | 2 | 77 | 0.17 | 0.848 |
|  | MTL*Process*Material-Type*Paradigm | 2 | 77 | 0.03 | 0.972 |

Results from two linear mixed-effects models: the ‘ROI model’, where the volumes of the 4 ROIs (HPC, ERC, PRC, PHC), averaged across hemispheres, are entered as predictors; the ‘MTL model’, where ‘MTL volume’ (the sum of HPC, ERC, PRC, and PHC volume) is entered as a predictor instead (along with the rest of the predictors and nuisance covariates). **Key: ROI:** region of interest; **PHC:** parahippocampal cortex; **PRC:** perirhinal cortex; **HPC:** hippocampus; **ERC:** entorhinal cortex; shaded cells: significant (p < 0.05) main effects and interactions of interest.

## Supplementary table 19: Continuous Analyses : Separate models for L / R HPC / ERC / PRC / PHC (including Words)

| **Model** | **Effect/Interaction** | **Df1** | **Df2** | **F** | **p** |
| --- | --- | --- | --- | --- | --- |
| ROI: Left HPC  **Predictors:**  ROI volume  Process (Recollection vs. Familiarity)  Material-Type (Faces vs. Scenes vs. Words)  Paradigm (ROC vs. RDP);  **Outcome variable:** (Recollection and Familiarity) estimates | Volume | 1 | 7 | 1.85 | 0.216 |
|  | Process | 1 | 77 | 11.85 | 0.001 |
|  | Material-Type | 2 | 77 | 0.36 | 0.699 |
|  | Paradigm | 1 | 77 | 1.97 | 0.164 |
|  | Volume*Process | 1 | 77 | 7.85 | 0.006 |
|  | Volume*Material-Type | 2 | 77 | 2.04 | 0.137 |
|  | Process*Material-Type | 2 | 77 | 0.37 | 0.691 |
|  | Volume*Paradigm | 1 | 77 | 2.56 | 0.114 |
|  | Process*Paradigm | 1 | 77 | 2.38 | 0.127 |
|  | Material-Type*Paradigm | 2 | 77 | 0.59 | 0.558 |
|  | Volume*Process*Material-Type | 2 | 77 | 0.47 | 0.626 |
|  | Volume*Process*Paradigm | 1 | 77 | < 0.05 | 0.996 |
|  | Volume*Material-Type*Paradigm | 2 | 77 | 0.57 | 0.570 |
|  | Process*Material-Type*Paradigm | 2 | 77 | < 0.05 | 0.995 |
|  | Volume*Process*Material-Type*Paradigm | 2 | 77 | 0.12 | 0.885 |
| ROI: Right HPC  **Predictors:**  ROI volume  Process (Recollection vs. Familiarity)  Material-Type (Faces vs. Scenes vs. Words)  Paradigm (ROC vs. RDP);  **Outcome variable:** (Recollection and Familiarity) estimates | Volume | 1 | 7 | 1.20 | 0.309 |
|  | Process | 1 | 77 | 13.18 | 0.001 |
|  | Material-Type | 2 | 77 | 0.59 | 0.560 |
|  | Paradigm | 1 | 77 | 2.45 | 0.121 |
|  | Volume*Process | 1 | 77 | 9.42 | 0.003 |
|  | Volume*Material-Type | 2 | 77 | 0.59 | 0.558 |
|  | Process*Material-Type | 2 | 77 | 0.72 | 0.489 |
|  | Volume*Paradigm | 1 | 77 | 2.91 | 0.092 |
|  | Process*Paradigm | 1 | 77 | 0.35 | 0.555 |
|  | Material-Type*Paradigm | 2 | 77 | 1.12 | 0.333 |
|  | Volume*Process*Material-Type | 2 | 77 | 0.57 | 0.567 |
|  | Volume*Process*Paradigm | 1 | 77 | 0.56 | 0.458 |
|  | Volume*Material-Type*Paradigm | 2 | 77 | 0.98 | 0.380 |
|  | Process*Material-Type*Paradigm | 2 | 77 | 0.10 | 0.908 |
|  | Volume*Process*Material-Type*Paradigm | 2 | 77 | < 0.05 | 0.975 |
| ROI: Left PRC  **Predictors:**  ROI volume  Process (Recollection vs. Familiarity)  Material-Type (Faces vs. Scenes vs. Words)  Paradigm (ROC vs. RDP);  **Outcome variable:** (Recollection and Familiarity) estimates | Volume | 1 | 7 | < 0.05 | 0.987 |
|  | Process | 1 | 77 | 4.17 | 0.045 |
|  | Material-Type | 2 | 77 | 2.79 | 0.068 |
|  | Paradigm | 1 | 77 | 0.50 | 0.483 |
|  | Volume*Process | 1 | 77 | 1.00 | 0.320 |
|  | Volume*Material-Type | 2 | 77 | 0.71 | 0.494 |
|  | Process*Material-Type | 2 | 77 | 0.65 | 0.523 |
|  | Volume*Paradigm | 1 | 77 | 0.84 | 0.363 |
|  | Process*Paradigm | 1 | 77 | 5.07 | 0.027 |
|  | Material-Type*Paradigm | 2 | 77 | 0.11 | 0.892 |
|  | Volume*Process*Material-Type | 2 | 77 | 0.14 | 0.870 |
|  | Volume*Process*Paradigm | 1 | 77 | 0.06 | 0.806 |
|  | Volume*Material-Type*Paradigm | 2 | 77 | 0.18 | 0.838 |
|  | Process*Material-Type*Paradigm | 2 | 77 | 1.47 | 0.235 |
|  | Volume*Process*Material-Type*Paradigm | 2 | 77 | 1.48 | 0.234 |
| ROI: Right PRC  **Predictors:**  ROI volume  Process (Recollection vs. Familiarity)  Material-Type (Faces vs. Scenes vs. Words)  Paradigm (ROC vs. RDP);  **Outcome variable:** (Recollection and Familiarity) estimates | Volume | 1 | 7 | 0.41 | 0.543 |
|  | Process | 1 | 77 | 0.73 | 0.394 |
|  | Material-Type | 2 | 77 | 11.11 | < 0.001 |
|  | Paradigm | 1 | 77 | 0.18 | 0.675 |
|  | Volume*Process | 1 | 77 | 1.68 | 0.199 |
|  | Volume*Material-Type | 2 | 77 | 2.80 | 0.067 |
|  | Process*Material-Type | 2 | 77 | 2.22 | 0.115 |
|  | Volume*Paradigm | 1 | 77 | 0.46 | 0.501 |
|  | Process*Paradigm | 1 | 77 | 9.46 | 0.003 |
|  | Material-Type*Paradigm | 2 | 77 | 0.72 | 0.490 |
|  | Volume*Process*Material-Type | 2 | 77 | 0.86 | 0.426 |
|  | Volume*Process*Paradigm | 1 | 77 | 0.62 | 0.434 |
|  | Volume*Material-Type*Paradigm | 2 | 77 | 0.42 | 0.661 |
|  | Process*Material-Type*Paradigm | 2 | 77 | 1.19 | 0.309 |
|  | Volume*Process*Material-Type*Paradigm | 2 | 77 | 0.74 | 0.481 |
| ROI: Left ERC  **Predictors:**  ROI volume  Process (Recollection vs. Familiarity)  Material-Type (Faces vs. Scenes vs. Words)  Paradigm (ROC vs. RDP);  **Outcome variable:** (Recollection and Familiarity) estimates | Volume | 1 | 7 | 0.28 | 0.615 |
|  | Process | 1 | 77 | 2.13 | 0.148 |
|  | Material-Type | 2 | 77 | 1.90 | 0.157 |
|  | Paradigm | 1 | 77 | 0.32 | 0.571 |
|  | Volume*Process | 1 | 77 | < 0.05 | 0.879 |
|  | Volume*Material-Type | 2 | 77 | 1.27 | 0.285 |
|  | Process*Material-Type | 2 | 77 | 1.53 | 0.223 |
|  | Volume*Paradigm | 1 | 77 | 0.60 | 0.442 |
|  | Process*Paradigm | 1 | 77 | 3.51 | 0.065 |
|  | Material-Type*Paradigm | 2 | 77 | < 0.05 | 0.988 |
|  | Volume*Process*Material-Type | 2 | 77 | 0.21 | 0.809 |
|  | Volume*Process*Paradigm | 1 | 77 | 0.17 | 0.683 |
|  | Volume*Material-Type*Paradigm | 2 | 77 | 0.42 | 0.657 |
|  | Process*Material-Type*Paradigm | 2 | 77 | 0.83 | 0.439 |
|  | Volume*Process*Material-Type*Paradigm | 2 | 77 | 0.32 | 0.729 |
| ROI: Right ERC  **Predictors:**  ROI volume  Process (Recollection vs. Familiarity)  Material-Type (Faces vs. Scenes vs. Words)  Paradigm (ROC vs. RDP);  **Outcome variable:** (Recollection and Familiarity) estimates | Volume | 1 | 7 | 4.70 | 0.067 |
|  | Process | 1 | 77 | 0.52 | 0.474 |
|  | Material-Type | 2 | 77 | 1.64 | 0.200 |
|  | Paradigm | 1 | 77 | 0.90 | 0.347 |
|  | Volume*Process | 1 | 77 | 0.57 | 0.454 |
|  | Volume*Material-Type | 2 | 77 | 0.62 | 0.543 |
|  | Process*Material-Type | 2 | 77 | 0.14 | 0.870 |
|  | Volume*Paradigm | 1 | 77 | 1.53 | 0.220 |
|  | Process*Paradigm | 1 | 77 | 3.71 | 0.058 |
|  | Material-Type*Paradigm | 2 | 77 | 0.11 | 0.892 |
|  | Volume*Process*Material-Type | 2 | 77 | 1.27 | 0.286 |
|  | Volume*Process*Paradigm | 1 | 77 | < 0.05 | 0.856 |
|  | Volume*Material-Type*Paradigm | 2 | 77 | 0.27 | 0.766 |
|  | Process*Material-Type*Paradigm | 2 | 77 | 0.13 | 0.879 |
|  | Volume*Process*Material-Type*Paradigm | 2 | 77 | 0.03 | 0.972 |
| ROI: Left PHC  **Predictors:**  ROI volume  Process (Recollection vs. Familiarity)  Material-Type (Faces vs. Scenes vs. Words)  Paradigm (ROC vs. RDP);  **Outcome variable:** (Recollection and Familiarity) estimates | Volume | 1 | 7 | 3.32 | 0.111 |
|  | Process | 1 | 77 | 0.66 | 0.417 |
|  | Material-Type | 2 | 77 | 5.10 | 0.008 |
|  | Paradigm | 1 | 77 | 1.19 | 0.279 |
|  | Volume*Process | 1 | 77 | 7.36 | 0.008 |
|  | Volume*Material-Type | 2 | 77 | 0.89 | 0.414 |
|  | Process*Material-Type | 2 | 77 | 0.54 | 0.585 |
|  | Volume*Paradigm | 1 | 77 | 1.87 | 0.175 |
|  | Process*Paradigm | 1 | 77 | 0.63 | 0.429 |
|  | Material-Type*Paradigm | 2 | 77 | 0.43 | 0.651 |
|  | Volume*Process*Material-Type | 2 | 77 | 3.04 | 0.054 |
|  | Volume*Process*Paradigm | 1 | 77 | 2.81 | 0.098 |
|  | Volume*Material-Type*Paradigm | 2 | 77 | 0.16 | 0.849 |
|  | Process*Material-Type*Paradigm | 2 | 77 | 0.34 | 0.713 |
|  | Volume*Process*Material-Type*Paradigm | 2 | 77 | 0.95 | 0.393 |
| ROI: Right PHC  **Predictors:**  ROI volume  Process (Recollection vs. Familiarity)  Material-Type (Faces vs. Scenes vs. Words)  Paradigm (ROC vs. RDP);  **Outcome variable:** (Recollection and Familiarity) estimates | Volume | 1 | 7 | 4.96 | 0.061 |
|  | Process | 1 | 77 | 0.17 | 0.679 |
|  | Material-Type | 2 | 77 | 5.70 | 0.005 |
|  | Paradigm | 1 | 77 | 0.27 | 0.606 |
|  | Volume*Process | 1 | 77 | 8.99 | 0.004 |
|  | Volume*Material-Type | 2 | 77 | 0.50 | 0.607 |
|  | Process*Material-Type | 2 | 77 | 1.25 | 0.292 |
|  | Volume*Paradigm | 1 | 77 | 0.58 | 0.449 |
|  | Process*Paradigm | 1 | 77 | 5.07 | 0.027 |
|  | Material-Type*Paradigm | 2 | 77 | 0.17 | 0.846 |
|  | Volume*Process*Material-Type | 2 | 77 | 0.51 | 0.600 |
|  | Volume*Process*Paradigm | 1 | 77 | 0.13 | 0.715 |
|  | Volume*Material-Type*Paradigm | 2 | 77 | 0.07 | 0.935 |
|  | Process*Material-Type*Paradigm | 2 | 77 | 0.95 | 0.391 |
|  | Volume*Process*Material-Type*Paradigm | 2 | 77 | 2.05 | 0.136 |

Results from fitting a series of linear mixed-effects models, for recollection and familiarity estimates, across all patients (n=9). One model was fit for each ROI: L / R HPC / ERC / PRC / PHC. Each model involved the following predictors: ROI volume; Paradigm (ROC vs. RDP); Process (Recollection vs. Familiarity); Material Type (Faces vs. Scenes vs. Words). **Key: ROI:** region of interest; **PHC:** parahippocampal cortex; **PRC:** perirhinal cortex; **HPC:** hippocampus; **ERC:** entorhinal cortex; shaded cells: significant (p < 0.05) main effects and interactions of interest.

# Supplementary References

Argyropoulos GPD, Loane C, Roca-Fernandez A, Lage-Martinez C, Gurau O, Irani SR, Butler CR. 2019. Network-wide abnormalities explain memory variability in hippocampal amnesia. *Elife* **8**. doi:10.7554/eLife.46156

Baddeley A, Emslie H, Nimmo-Smith I. 1994. Doors and people: a test of visual and verbal recall and recognition. Bury St. Edmunds, England: Thames Valley Test Co.

Bozeat S, Lambon Ralph MA, Patterson K, Garrard P, Hodges JR. 2000. Non-verbal semantic impairment in semantic dementia. *Neuropsychologia* **38**:1207–1215. doi:10.1016/S0028-3932(00)00034-8

Delis DCD, Kaplan E, Kramer JH, Delis DC, Kaplan E, Kramer JH. 2001. Delis-Kaplan executive function system (D-KEFS), Canadian Journal of School Psychology. Psychological Corporation.

Mckenna P, Warrington EK. 1980. Testing for nominal dysphasia. *J Neurol Neurosurgery, Psychiatry* **43**:781–788. doi:10.1136/jnnp.43.9.781

Nelson HE, Willison J. 1991. National Adult Reading Test (NART): Test manual.

Rey A. 1959. Manuel du test de copie d’une figure complexe de A. Rey. *Paris Les Ed du Cent Psychol Appliquée*.

Warrington E. 1984. The Recognition Memory Test. Windsor, UK: NFER-Nelson.

Warrington EK. 1996. The Camden Memory Tests. Psychology Press.

Warrington EK, James M. 1991. The Visual Object and Space Perception Battery, Thames Valley Test Company.

Wechsler D. 2011. WASI -II: Wechsler abbreviated scale of intelligence - second edition, WASI. Psychological Corporation.

Wechsler D. 1997. Wechsler Memory Scale- (Third Ed.). *Psychol Corp*.
